# Dr.Nod: computational framework for discovery of regulatory non-coding drivers in tissue-matched distal regulatory elements

**DOI:** 10.1093/nar/gkac1251

**Published:** 2023-01-10

**Authors:** Marketa Tomkova, Jakub Tomek, Julie Chow, John D McPherson, David J Segal, Fereydoun Hormozdiari

**Affiliations:** Department of Biochemistry and Molecular Medicine, University of California, Davis, CA 95616, USA; Ludwig Cancer Research, University of Oxford, Oxford, OX3 7DQ, UK; UC Davis Genome Center, University of California, Davis, CA 95616, USA; Department of Pharmacology, University of California, Davis, CA 95616, USA; Department of Biochemistry and Molecular Medicine, University of California, Davis, CA 95616, USA; Department of Biochemistry and Molecular Medicine, University of California, Davis, CA 95616, USA; Department of Biochemistry and Molecular Medicine, University of California, Davis, CA 95616, USA; UC Davis Genome Center, University of California, Davis, CA 95616, USA; UC Davis MIND Institute, University of California, Davis, CA 95616, USA; Department of Biochemistry and Molecular Medicine, University of California, Davis, CA 95616, USA; UC Davis Genome Center, University of California, Davis, CA 95616, USA; UC Davis MIND Institute, University of California, Davis, CA 95616, USA

## Abstract

The discovery of cancer driver mutations is a fundamental goal in cancer research. While many cancer driver mutations have been discovered in the protein-coding genome, research into potential cancer drivers in the non-coding regions showed limited success so far. Here, we present a novel comprehensive framework Dr.Nod for detection of non-coding *cis*-regulatory candidate driver mutations that are associated with dysregulated gene expression using tissue-matched enhancer-gene annotations. Applying the framework to data from over 1500 tumours across eight tissues revealed a 4.4-fold enrichment of candidate driver mutations in regulatory regions of known cancer driver genes. An overarching conclusion that emerges is that the non-coding driver mutations contribute to cancer by significantly altering transcription factor binding sites, leading to upregulation of tissue-matched oncogenes and down-regulation of tumour-suppressor genes. Interestingly, more than half of the detected cancer-promoting non-coding regulatory driver mutations are over 20 kb distant from the cancer-associated genes they regulate. Our results show the importance of tissue-matched enhancer-gene maps, functional impact of mutations, and complex background mutagenesis model for the prediction of non-coding regulatory drivers. In conclusion, our study demonstrates that non-coding mutations in enhancers play a previously underappreciated role in cancer and dysregulation of clinically relevant target genes.

## INTRODUCTION

One of the main goals of cancer research is the discovery of cancer driver mutations and their use in the development of targeted cancer therapies (1). Most of these efforts have focused on protein-coding mutations, given the availability of tumour whole-exome sequencing data and the direct functional impact. With the increasing number of available tumour whole-genome sequencing (WGS) data, there is a growing interest in understanding the role of non-coding somatic mutations in cancer. Discovery of non-coding drivers is important for our understanding of tumour biology, identification of novel biomarkers and potential drug targets. However, the search for non-coding drivers has proved surprisingly difficult and resulted in only a small number of proposed and validated non-coding drivers, with the promoter of *TERT* gene being the key credible example (2,3). In a recent review article, Elliott and Larsson listed several potential reasons for the relative paucity of credible non-coding drivers so far (2). Here, we address some of these key challenges, as summarised below, and comprehensively study putative non-coding drivers in tissue-matched regulatory elements, focusing on mutations predicted to regulate gene expression.

The first challenge lies in the vast size of the non-coding genome and the currently limited statistical power for unbiased search of all the potential non-coding driver elements. To reduce the searched space, we focus only on *cis*-regulatory elements (enhancers and some promoters) of genes expressed in given tissue, as they remain relatively understudied but showed promising potential (4), and we predicted that mutations in *cis*-regulatory elements may contribute to cancer by altering expression of cancer-relevant genes.

Second, the driver mutations regulating one gene may be spread over larger genomic distances due to tissue-specific *cis*-regulatory long-range interactions (2,4). To our knowledge, annotations of tissue-specific enhancer-gene interactions have not yet been used for detection of non-coding regulatory drivers in a pan-cancer study. To address this challenge, we made use of tissue-specific enhancer-gene maps from a recent successful Activity-by-Contact (ABC) method (5,6). The ABC method was previously validated using CRISPRi-FlowFISH perturbation experiments (5) and was shown to successfully predict causal disease variants from genome-wide association studies and link them to their target genes (6,7). Here, we search for non-coding *cis*-regulatory cancer driver mutations using the tissue-specific enhancer-gene maps predicted by the ABC method.

The third major challenge lies in modelling the background mutation rate and predicting the functional effect of the non-coding mutations. Traditionally, cancer driver elements (regions under positive selection) are identified as regions mutated more frequently than expected by chance. However, the expected mutation rate (‘background mutagenesis’) is highly variable across the genome and between cancers (1,2,8). Background mutagenesis is affected by many genomic features, such as DNA accessibility (9), sequence context (10), histone and DNA modifications (11–14), replication timing (15,16), localised mutagenesis (17) and other features (8). A background mutagenesis model can be built and used to predict the driver regions mutated with frequency above expectation. However, it is assumed that the current knowledge of the background mutagenesis is still rather incomplete and may not be on its own sufficient to distinguish between the true driver vs passenger mutations (1,2). Therefore, we define our putative drivers based on two additional requirements on functional impact of the mutations to enrich for driver mutations: (i) high Combined Annotation Dependent Depletion (CADD) score of pathogenicity (18), and (ii) predicted regulatory impact of the mutations on gene expression.

We applied our newly developed methodology on over 1500 WGS cancers from across 8 tissues from the Pan-Cancer Analysis of Whole Genomes (PCAWG) dataset (19) and tissue-matched enhancer-gene maps for the eight tissues. We observed a strong enrichment of known cancer driver genes (CDGs) within the target genes of the candidate regulatory drivers. Our candidate regulatory driver mutations are predicted to significantly alter transcription factor binding sites (TFBS). Moreover, the candidate driver mutations are predicted to contribute to upregulation of oncogenes and cancer-essential genes and downregulation of tumour-suppressor genes in the matched tissues. Our findings show the importance of tissue-specific epigenomic annotations and requirements on functional impact of mutations in predicting non-coding *cis*-regulatory drivers. More generally, the results identify a previously underappreciated role of the non-coding genome in cancer in a tissue-specific manner.

## MATERIALS AND METHODS

### Cancer samples

The analysis was performed on 1575 donors from the PCAWG project (19). Only donors that pass all the stringent PCAWG quality control criteria were included and only one sample was used per donor. The somatic mutations, gene expression, copy number variation, and structural variant calls were obtained from the ICGC Data Portal (https://dcc.icgc.org/releases/PCAWG/). To avoid confounding the signal by known sources of hypermutation, samples with a contribution of POLE-MUT (SBS10a, SBS10b, DBS3) or MSI signatures (SBS6, SBS14, SBS15, SBS20, SBS21, SBS44, DBS7, DBS10, ID7) over 20% were excluded from the analysis. The SigProfiler calls of mutational signatures from the ICGC Data Portal were used for this purpose. The criteria for tissue inclusion were the existence of over 40 PCAWG donors with RNA-seq data and availability of an ABC enhancers map from a matched tissue. In total, eight tissues passed the criteria and were included in the analysis: blood (197 donors, of them 173 with RNA: 197/173), brain (287/46), breast (211/91), colorectal (42/41), liver (332/116), lung (84/84), ovary (110/89), and pancreas (312/75).

### Regulatory regions

The ABC enhancer-gene maps were obtained from Nasser *et al.* (6) (Supplementary Table S1). All coding regions were excluded (based on the hg19 GENCODE reference) and each enhancer region was extended by 250 bp both upstream and downstream. For every gene, we then defined the ABC regulatory space of that gene by pooling together all the non-coding enhancer regions that are predicted to regulate that gene based on the tissue-matched ABC enhancer-gene map. The hg19 reference genome was used for the entire project.

### CADD score filtering

The CADD score annotations (18) of all SNVs were obtained from https://cadd.gs.washington.edu/download. As potential non-coding driver candidate mutations, only non-coding SNVs with CADD PHRED score of at least 10 (termed *high-CADD SNVs*) were considered. The effect of this cut-off on overall results was explored in Figure 2D. Since some regions may have a generally higher chance of having SNVs with a high-CADD score and this could confound the results, we accounted for this in the background mutagenesis model. To this end, we computed the number of *theoretical high-CADD mutations* with a CADD PHRED score of at least 10 in the given non-coding region using BEDTools (20). Each genomic position can generate up to three theoretical high-CADD mutations (in case when all three alternative alleles at a locus lead to a high-CADD SNV score; for example, a position with base C can generate mutations C > A, C > G, and C > T). The high-CADD mutation frequency score was then defined as the fraction of the number of high-CADD SNVs over the number of theoretical high-CADD mutations.

### Non-coding regulatory driver candidates

For every expressed gene with ABC enhancers in the given tissue, we computed *score_M_* and *score_E_* to represent the likelihood of the ABC regulatory space of the gene being a regulatory driver candidate based on observed somatic non-coding high-CADD SNV mutations and alteration of gene expression, respectively. The *score_M_* is computed as −log_10_(*p_M_*), where the *P*-value *p_M_* measures whether the observed non-coding high-CADD SNVs in the gene's regulatory space exceed the expected value predicted by a background mutagenesis model (details below). The *score_E_* is computed as −log_10_(*p_E_*), where the *P*-value *p_E_* measures the differential expression of the target gene between samples with and without mutations in the regulatory space of the gene (details below). We then combined *p_M_* and *p_E_* with the Brown's method (21) (in the same way as it has been used by Rheinbay *et al.* (3)) and used the Benjamini-Hochberg method (MATLAB function mafdr(pCombined, ‘BHFDR’, true)) to obtain a combined *q*-value. The *non-coding regulatory drivercandidate* is then defined as having *p_M_* <0.05 and *p_E_* <0.05 and combined *q*-value <0.15 (false-discovery rate < 15%). In this way, we ensure that all the candidates have high values of both the *score_M_* and *score_E_*, as well as being statistically significant when both underlying *P*-values are combined. Each driver candidate represents merged discontinuous non-coding parts of regions regulating the given gene and each regulatory region can potentially contribute to more than a single gene.

#### Background mutagenesis model and the *P*-value p_M_

We modelled the background mutagenesis based on Poisson Generalised Linear Model (GLM) regression, similar as in the ActiveDriverWGS (4) and DriverPower (22) methods. The model was fitted using the MATLAB function fitglm(data, ‘linear’, ‘Distribution’, ‘poisson’, ‘DispersionFlag’, true) for each tissue independently, with one data point per each gene expressed in the tissue. The response variable was the high-CADD mutation frequency in the non-coding regulatory space of the gene, computed as $\frac{n}{{s\ k\ }}$, where *n* is the number of observed high-CADD mutated samples, *s* is the number of theoretical high-CADD mutations in the given non-coding regulatory space, and *k* is the number of samples in the tissue. For each gene, the maximum of 1 mutation per sample was considered, to reduce the effect of local hypermutation. The explanatory variables (previously identified as important predictors of mutational variation (8)) were: the frequency of each of the 32 trinucleotides (with C or T as the ref allele), the number of positions, the average GC content, the average replication timing (data as in Tomkova *et al.* (15), from Haradhvala *et al.* (16)), the tissue-specific ABC ‘base activity’ representing the DNase-seq and H3K27ac signal of the given ABC enhancer-gene map, and the local mutation frequency. The local mutation frequency was computed as the average mutation frequency in the ±50 kb flanking regions of each of the enhancers, similarly as in the ActiveDriverWGS method (4). The coding regions and ABC enhancer regions were excluded from the local mutation frequency computation to best capture the underlying local background mutagenesis as opposed to positive selection. For regulatory space consisting of multiple disjoint segments, the (non-coding non-enhancer) flanking regions of each of these segments were included and pooled together. To reduce the risk of overfitting, only highly predictive explanatory variables (*P*-value < 0.001 in an univariable model) were included in the multivariable model. The model was computed for each tissue independently, to better capture the different mutational processes operating in each of the tissues. The model was then used to predict *f*, which for each gene represents the expected mutation frequency per theoretical high-CADD mutation per sample in the regulatory space of the gene. The *P*-value *p_M_* was then computed as the right-sided binomial test comparing the observed and expected high-CADD mutations as BinomTest(*n*, *k*, *p*, ‘one’), where *n* is the number of samples with a high-CADD mutation in the regulatory space, *k* is the number of samples in the tissue, and *p* represents the probability of a sample having at least one high-CADD mutation in the regulatory space (1 − the probability that none of the theoretical high-CADD mutations have a high-CADD mutations in a given sample). The value of *p* is computed as $p\ = \ 1 - {( {1 - f} )}^s$, where *f* is the expected mutation frequency per theoretical high-CADD mutation per sample (computed using the background mutagenesis model) and *s* is the number of theoretical high-CADD mutations. Of note, after initial exploration of the effect of indels, only (high-CADD) SNVs were included in the analysis.

#### Gene expression model and the *P*-value p_E_

To compare gene expression between the donors with vs without high-CADD mutations in the regulatory space of the gene, we modelled the gene expression using Poisson Generalised Linear Model (GLM) regression as FPKM-UQ ∼ MUT + CNV, using MATLAB function fitglm(data, ‘linear’, ‘Distribution’, ‘poisson’, ‘DispersionFlag’, true). The response variable FPKM-UQ was the upper quartile normalised gene expression (FPKM-UQ) downloaded from the ICGC Data Portal of the PCAWG dataset. The binary explanatory variables MUT is 1 for donors with a high-CADD mutation in the non-coding regulatory space of the gene and 0 for all other donors in the tissue. The explanatory variable CNV is the gene-level somatic copy number variation. The *P*-value of the t-statistic of the MUT variable (mdl.Coefficients.pValue (2)) is then used as the *P*-value *p_E_* and the Estimate (mdl.Coefficients.Estimate (2)) is used as the size effect of the MUT variable (positive for upregulation in the mutated donors, negative for downregulation in the mutated donors). To prevent spurious signal driven by a single mutation, *NaN* was assigned to *p_E_* in genes with fewer than 2 SNVs in the regulatory space in samples with RNA-seq data.

#### Non-coding regulatory driver candidates

All the expressed genes with predicted ABC enhancers and PCAWG expression data were included in the analysis. The gene mapping was performed based on the symbol name and location of the TSS. For every gene (its regulatory space), the *p_M_* and *p_E_ P*-values were computed and then those with *p*_M_ <0.05 and *p_E_ <*0.05 and combined *q*-value <0.15 were defined as the (non-coding) *regulatory driver candidates*. As a *candidate regulatory driver mutation*, we count all high-CADD SNVs (CADD PHRED ≥ 10) in the regulatory driver candidate regions. It is possible that some of the low-CADD SNVs in these regions may also act as drivers. Therefore, in the TFBS analysis, we explore both high-CADD only and all-CADD SNVs in the regulatory driver candidate regions. At the same time, we expect that majority but not all of these mutations will act as *bona fide* drivers, and therefore we use the term ‘candidate’ driver.

Candidate driver-upregulated and driver-downregulated genes. We annotated the target genes of the regulatory driver candidates as candidate driver-upregulated genes (upregulated in enhancer-mutated samples), or candidate driver-downregulated genes (downregulated in enhancer-mutated samples) based on the sign of the Estimate value in the gene-expression model.

#### Pan-cancer analysis

The analysis above was performed independently for every tissue. For the pan-cancer analysis, we used all the genes that are expressed and have predicted ABC enhancers in at least one of the tissues. The pan-cancer regulatory driver candidates were defined as being a regulatory driver candidate in any of the tissue-level analyses.

### Evaluation of background mutagenesis model

To evaluate the background mutagenesis model, we performed a 6-fold cross-validation. First, we split the genome into 6-fold of approximately similar size by chromosomes (chromosomes in folds: 1–2, 3–5, 6–8, 9–11, 12–16, 17–22). For each fold, the larger part was used for model training, while the smaller part for model evaluation. The performance was quantified using explained variance, calculated as the square of the Pearson correlation coefficient between the predicted and observed SNV counts, as used previously (23–25). Similarly, as in these studies, sex chromosomes and elements in the top 99th percentile of mutation count have been excluded from the training and test sets in the model evaluation analysis. In our evaluation, only non-coding regulatory regions were used.

### Cancer driver genes, prognostic genes, and cancer essential genes

#### Cancer driver gene (CDG) annotation

The CDGs were defined as union of CDGs defined by Cancer Gene Census (CGC) downloaded from the COSMIC Data Portal (https://cancer.sanger.ac.uk/census) and of CDGs defined by the PCAWG analysis (Supplementary Tables S1 and S3 in https://dcc.icgc.org/releases/PCAWG/driver_mutations, only coding drivers were taken into account).

#### CDG enrichment

The log_2_ fold enrichment of CDGs in the target genes of the regulatory driver candidates was computed as $log_{2}({^{O}}/{_{E}})$, where *O* is the number of observed CDGs in the driver candidate target genes and *E* is the number of expected CDGs in driver candidate target genes by chance, computed as $E = ^{({R \times D})}/_{G_{D}} $ where *R* is the number of regulatory driver candidates, *D* is the number of CDGs, and *G* is the number of all genes. When computing the *O*, *E*, *R*, *D* and *G* values, we include only expressed genes with ABC enhancers in the given tissue. The *P*-value of the enrichment was computed using a two-tailed Fisher's exact test.

#### Pan-cancer oncogenes and tumour-suppressor genes (TSGs)

We used the pan-cancer CGC annotation of oncogenes and TSGs (26). We then used two-tailed Fisher's exact test to evaluate the enrichment of oncogenes in driver-upregulated genes (by comparing driver-upregulated genes vs genes not regulated by regulatory driver candidates, and oncogenes versus non-CDGs) and TSGs in driver-downregulated genes (by comparing driver-downregulated genes versus genes not regulated by regulatory driver candidates, and TSGs versus non-CDGs).

#### Tissue-specific oncogenes and TSGs

We systematically searched the existing literature for any evidence for a potential tissue-specific role as oncogenes or TSGs in the target genes of the detected regulatory driver candidates in solid cancers. For the search, we used the combination of the gene symbol (also checking for alternative/previous gene or protein names), and the cancer type (e.g. lung cancer, lung adenocarcinoma, brain cancer, medulloblastoma, etc.) on PubMed.gov, MalaCards.org (27), and Google Scholar. The following observations were considered strong evidence of being an oncogene or a TSG: experimental studies directly showing a promoting or protecting effect on tumour formation, proliferation, apoptosis, metastases etc. or use of the gene as a drug target in ongoing or published clinical studies. The following observations were considered weak evidence: studies claiming the oncogenic/TSG role based on combined indirect evidence, such as increased/decreased expression compared to neighbouring normal tissue, prognostic effect and computational/in silico studies, usually supported also by experimental evidence in other tissues. The evidence considered for each individual gene is listed in Supplementary Table S3.

#### Prognostic genes

For an unbiased comparison of the predictive value of high versus low expression of the candidate genes for survival, we downloaded the tissue-specific prognostic predictions from The Human Protein Atlas (28). The tissue-specific *P*-values are listed in Supplementary Table S3.

#### Cancer essential genes in DepMap (Figure 4E, Supplementary Figure S1)

Finally, as an orthogonal unbiased way to evaluate the importance of the identified driver-upregulated genes in cancer, we utilised data from the Dependency Map (DepMap) Achilles project (29). In this project, the effect of gene knockout on the proliferation and survival of cancer cells is measured across hundreds of cell lines using CRISPR/Cas9 screens. As a result, a dependency score is estimated for every gene and every cell line (0 represents low dependency/essentiality, 1 represents high dependency/essentiality). We downloaded the Achilles dependency scores (Achilles_gene_dependency.csv) and the expression values (CCLE_expression.csv) of the genes in the same cell lines from the DepMap portal (https://depmap.org/portal/) version 22Q2. For every gene, we computed the average dependency score (a) across all cell lines and (b) across cell lines of a given tissue, considering only genes that have expression value above 1 transcript per million (TPM) in the given cell line. Then we compared the (a) average dependency scores and (b) percentage of dependent cell lines (dependency score > 0.5) between the driver-upregulated genes and non-candidate genes and used a two-tailed Wilcoxon rank-sum test to compare the two groups. This analysis was performed pan-cancer in the solid cancers, as well as independently in each tissue (taking only genes expressed in the tissue and cell lines of that tissue). Next, the percentage of genes with at least one tissue-matched dependent cell line (cell line, where the gene is essential, defined as having dependency score > 0.5) was compared for the driver-upregulated genes vs all genes not regulated by the regulatory driver candidates and evaluated using two-tailed Fisher's exact test. To investigate whether these genes are more frequently essential in the matched tissue compared to other tissues, we repeated the analysis with cell lines from unmatched tissue in the following way. For every tissue, we selected a subset of *k* tissue-unmatched cell lines, where *k* is number of tissue-matched cell lines, and computed the fold-change enrichment, all over 10 000 iterations. Then we computed a *P*-value of the tissue-matched versus unmatched results as the proportion of the distribution being more extreme than the tissue-matched value (computed as 2× of the lower one-tailed *P*-values).

### Robustness analyses

#### Cross-tissue analysis (Figure 2C)

For the cross-tissue analysis, we compared the CDG enrichment computed based on the ABC enhancer-gene maps for the matched tissue, versus all the seven other non-matched tissues. Apart from the input data used for regulatory region definition, the exact same analysis pipeline, computations and other types of input data were used.

#### CADD cut-off analysis (Figure 2D, Supplementary Figure S2)

For the CADD cut-off analysis, we explored the effect of the minimal allowed CADD PHRED score, evaluating values of 0, 2, …, 22. All the input data, analysis pipeline, and computations were identical to the main analysis. Outside this section, the CADD PHRED score cut-off value of 10 was used.

#### Effect of the pE (Figure 2B)

In this analysis, we compared the importance of *p*_E_ <0.05 condition in the definition of regulatory driver candidates. Using the same pipeline and data, we compared the CDG enrichment results when defining regulatory driver candidates as *p*_M_ <0.05 and *p*_E_ <0.05 and combined *q*-value < 0.15 versus (b) *p*_M_ <0.05.

#### *P*-value cut-off analysis (Supplementary Figure S3)

Here, we compared the CDG enrichment results for a range of *p_M_*, *p_E_*, and combined q-value cut-off values.

#### Mutational signatures (Figure 2E)

Here, we asked whether most of the predicted driver mutations in a given tissue are due to a single mutational process. Such a scenario could suggest that these mutations are not *bona fide* cancer drivers, but instead they represent false positive hits driven by a localised mutational process. To do so, we pooled the list of all high-CADD SNVs in the regulatory driver candidates in each tissue, calling them candidate driver mutations. We next annotated the mutations by their 5’ and 3’ sequence context, ref, and alt alleles, in the same way as when computing the mutational signatures (30,31). Then we computed the cosine similarity of these candidate driver *mutational profiles* with each of the COSMIC (31) v3.2 mutational signatures (https://cancer.sanger.ac.uk/signatures/). The cosine similarity gives values between 0 and 1, where 0 represents completely different signatures, 1 represents identical signatures, and values above ca 0.8 (sometimes 0.9) are usually considered sufficiently similar. Both the mutation counts and signatures were normalised for the trinucleotide frequency in the relevant regions.

### Confidence analyses (post-hoc identification of potential false positives)

#### Local underestimation by background mutagenesis model (Supplementary Figures S4–S5)

When the observed mutation counts significantly exceed the mutation counts predicted by the model, the gene gets a high *scoreM* and it is assumed that the reason for the high mutation count is due to positive selection. However, if many genes in the region have high observed/expected mutation count in their regulatory space (and this is not driven by the same mutations in shared regulatory regions), then an alternative explanation is that the model wrongly underestimates the mutation frequency in that region. In general, such situations should not happen too frequently, thanks to the ‘flanking mutation frequency’ predictor (that is based on non-coding and non-regulatory regions in the ±50 kb). Nevertheless, it could happen that this is not sufficient to correctly predict the regional mutation frequency (or that the mutation frequency is increased only in regulatory elements in that region), and the goal of this analysis was to identify such situations and check whether any of these involve the regulatory driver candidates. To do so, for every detected regulatory driver target, we computed the observed/expected ratio in the neighbouring genes (in distance up to 100 kb). Then regulatory driver candidates with median value above 2× are considered as potential false positives, as the background mutagenesis model may have falsely underestimated the mutation frequency in this region. The value of 2× was selected based on the minimal fold-change in the 52 regulatory driver candidates in solid cancers, which is 2.6×.

#### Shared regulatory regions

Another source of potential false discoveries could result from highly hypermutated regulatory elements that are shared across regulatory spaces of several target genes. Then all the targets that show a significant difference in expression between the mutated and wild-type samples are called as candidate driver targets. However, such significant difference in expression could happen by chance. Therefore, for every pair (or set) of driver target genes that share a mutated regulatory region(s), we annotated the gene with the highest *scoreE* as a likely true positive call, and the other gene(s) as potential false positives.

#### Low expression size effect

Finally, the third group of potential false positives consists of genes with the absolute value of expression size effect below 0.4326, which corresponds to 33^rd^ quantile in targets without any tissue-matched cancer evidence in the literature.

### Transcription factor binding site (TFBS) analysis (Figure 6)

We used the FunSeq2 (32) tool to predict the effect of SNVs on TFBS. We downloaded the annotated hg19 genome version 2.1.6 from http://funseq2.gersteinlab.org/downloads and used BEDTools (20) intersect to annotate all PCAWG mutations with the predicted effect on TFBS. Next, we compared the number of motif-breaking and motif-gaining events in the SNV mutations in the regulatory regions of the 48 driver-upregulated genes and 4 driver-downregulated genes and compared these values to the expected numbers based on all SNV mutations (a) in any ABC enhancer across all tissues, and (b) genome-wide. We used two-tailed Fisher's exact test to evaluate the enrichment. The TFBS motifs in Figure 6D and E were visualised using a custom script in MATLAB.

### Distance between regulatory driver mutations and their target genes (Figure 7, Supplementary Figure S6)

Here we evaluated the mutation-TSS distance for all M–G pairs of M = non-coding regulatory driver high-CADD SNV mutation and G = its differentially expressed target gene. For each M–G pair, we (a) computed and plotted the distance between the mutation and the TSS of the gene, (b) asked whether G is the closest gene to M (measured by the distance to the TSS of all genes), (c) as (b), but measuring distance to protein-coding genes only. Then we evaluated: (i) the percentage of M-G pairs with distance ≤ 250 bp (i.e. in the promoter of G), (ii) > 20 kb (i.e. in distal cis-regulatory element of G), (iii) where G is the closest gene (or protein coding gene).

### Candidate AID-generated regulatory cancer driver mutations in blood

#### Samples and genes

Only Diffuse Large B-Cell Lymphoma (DLBCL) patients were included in this analysis: PCAWG cohorts MALY-DE (100 donors, of them 98 with RNA: 100/98), which comprises only Germinal-centre B-cell-like (GCB) DLBCL, and DLBC-US (7/7). The main analysis was re-run on DLBCL subset of blood samples with identical parameters, but excluding mutations that occurred in up to 10 kb distance from an immunoglobulin gene (based on GENCODE gene type annotations). These genes are the direct targets of AID/SHM, and could therefore confound the analysis. Five candidate regulatory driver targets were excluded by this criterium (*CRIP1*, *MTA1*, *PPM1F*, *PRAMENP*, *TOP3B*).

#### List of ‘top hit’ mutations (the most promising regulatory cancer driver mutations)

The ‘top hit’ mutations were required to (a) disrupt TFBS of a TF annotated as a negative regulator of transcription or create *de novo* TFBS of a TF annotated as a positive regulator of transcription based on QuickGO annotations, (b) be high-CADD, (c) occur recurrently (in ≥2 patients for TFBS gain, in ≥3 patients for TFBS break) and (d) be associated with at least 3-fold increase or decrease of expression (FPKM-UQ) of their target gene. Only target genes of the regulatory driver candidates have been considered in this analysis (i.e. if a gene shows increased/decreased expression only for a subset of mutations, we did not include it in this analysis). The hits were defined by their hg19 genomic position, potentially grouping more alternative alleles together when both alleles created a TFBS event.

#### Annotation of TFs

Gene ontology (GO) from QuickGO (33) was used to annotate TFs as ‘positive regulation of transcription, DNA-templated’ (GO term 0045893) and ‘negative regulation of transcription, DNA-templated’ (GO term 0045892). Some TFs are annotated as both.

### Boxplots

On each box, the central mark indicates the median, and the bottom and top edges of the box indicate the 25th and 75th percentiles, respectively. The whiskers extend to the most extreme data points not considered outliers. An outlier is a value that is >1.5 times the interquartile range away from the bottom or top of the box. The individual data points are plotted on top of the boxplots.

## RESULTS

### Candidate non-coding regulatory drivers in PCAWG

We first built a tissue-specific background model of mutagenesis in regulatory regions (enhancers and some promoters), predicted by the tissue-specific ABC model based on H3K27ac, DNase-seq, and HiC data (6). In the background mutagenesis model, we accounted for replication timing, GC content, trinucleotide composition, length, tissue-matched DNase-seq and H3K27ac in the regulatory regions, and the local mutation frequency in the ±50 kb flanking regions (see Methods). We modelled the frequency of ‘high-CADD’ mutations (18), defined as single nucleotide variants (SNVs) with CADD PHRED ≥10, as those are predicted to be more likely pathogenic, and we accounted in the model for the regional CADD score differences across the regulatory regions. We performed a 6-fold cross-validation to evaluate how well the background mutagenesis model predicts mutations across the genome. The explained variance in unseen regions ranged between 20% and 60%, which is generally comparable to performance of previously published models (23) (Supplementary Figure S7). The explained variance increased with growing size of the tested regions (Supplementary Figure S8), as reported previously (13,23,25). Feature selection was stable across the 6 folds (Supplementary Figures S9 and S10) and between all-CADD and high-CADD model (Supplementary Figures S11 and S12).

**Figure 1.**
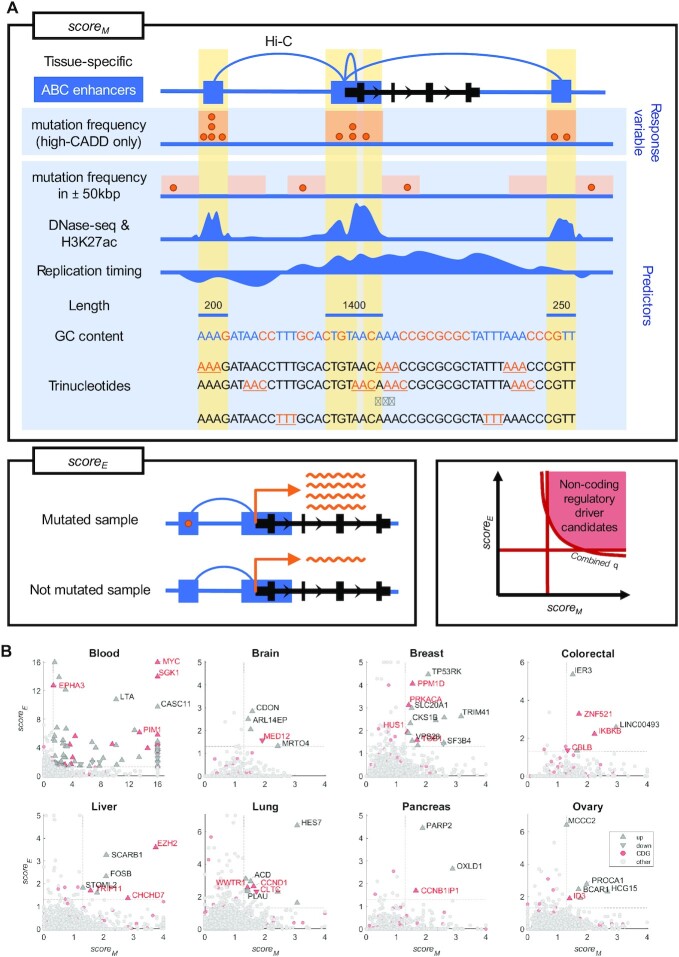
Overview of the method for detection of non-coding regulatory drivers. (**A**) For every gene, *score_M_* (top) and *score_E_* (bottom left) are computed based on the non-coding parts of the regulatory regions (ABC enhancers, shown as blue rectangles) of the gene. The *score_M_* represents the extent to which the regulatory regions are mutated above expectation, using a background mutagenesis model with high-CADD mutation frequency as the response variable and the other depicted features as predictors. The *score_E_* represents the extent to which mutations in the regulatory regions predict expression of the gene (mutated samples have either increased or decreased expression compared to the not mutated samples) in the given tissue. Target genes of non-coding regulatory driver candidates are then defined based on *score_M_*, *score_E_*, and combined q-value (bottom right). Each driver candidate represents merged discontinuous non-coding parts of regions regulating the given gene and each regulatory region can potentially contribute to more than a single gene. In order to increase readability, both axes are limited to the maximum of 16 (corresponding to *P*-value of 10^−16^) and the maximal value in candidate driver genes. (**B**) Target genes of non-coding regulatory driver candidates in PCAWG in the eight tissues. Gene upregulated/downregulated in the mutated samples are shown as upward/downward-pointing triangles, respectively. Known cancer driver genes (CDGs) are shown in red. Non-candidate genes are shown as light-grey circles.

The background mutagenesis model was used to search for regions under positive selection (more mutations than expected by the model). To increase the statistical power (and reduce the number of statistical tests), the non-coding regulatory driver candidates were defined on the level of genes. For every gene, we computed *score_M_*, representing whether the high-CADD mutations in the regulatory regions of the gene occur with frequency above expectation, and *score_E_*, representing whether the regulatory high-CADD mutations predict expression of the gene, after accounting for gene-level copy-number variation (Figure 1). Finally, we defined the *non-coding regulatory driver candidates* based on *score_M_*, *score_E_*, and combined *q*-value (see Materials and Methods).

**Figure 2.**
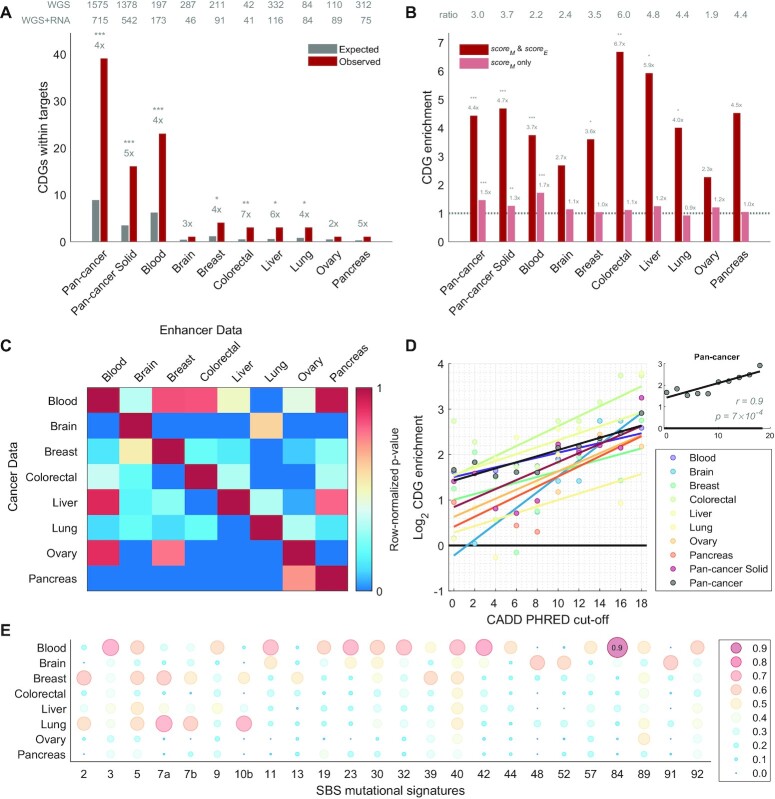
Cancer driver genes are enriched in tissue-specific targets of non-coding regulatory driver candidates. (**A**) The observed number of cancer driver genes (CDGs) within the target genes of non-coding regulatory driver candidates are shown in dark red. The expected numbers (in grey) are based on the CDG frequency in other genes. The grey numbers above each pair of bars denote the fold-change enrichment of observed vs. expected values and the stars represent the significance level based on the two-tailed Fisher's exact test (***P < 0.001; **P < 0.01; *P < 0.05). The two rows of numbers on top of the figure represent the number of WGS samples (top row) and WGS samples with RNA-seq available (bottom row) for each tissue. Pan-cancer represents all tissues together. Pan-cancer Solid represents all tissues except blood. (**B**) The CDG fold-change enrichment in target genes of non-coding regulatory driver candidates with the presented method (dark red) vs. if only *score_M_* (but not *score_E_*) was used (light red). The row of numbers on top of the figure represents the ratio between the two. (**C**) The heatmap shows the importance of tissue-specificity of the enhancer-gene maps. The colour represents the row-normalized *P*-value of the CDG enrichment (0 = least-significant *P*-value in the row, 1 = most-significant *P*-value in the row). The rows represent individual tissues of the cancer data (mutations and expression) in the PCAWG dataset, while the columns represent the tissue of the regulatory regions and the ABC enhancer-gene maps. (**D**) The log_2_ fold-change CDG enrichment (y-axis) when different cut-off values of the CADD PHRED score (x-axis) are used. A line was fitted through the data points in each tissue. The inset shows the pan-cancer results with the *r* and *P*-values of the Pearson correlation shown. (**E**) The cosine similarity of the mutational profiles of the candidate driver mutations with the COSMIC mutational signatures (columns), stratified by tissue (rows). Only signatures with cosine similarity >0.5 in at least one tissue are shown. Values >0.75 are shown also as text.

Utilizing the PCAWG dataset, we detected a median of 6.5 non-coding regulatory driver candidates per tissue (range 3–86) with no recurrent candidates between tissues (Figures 1B, 2A, Supplementary Figures S13 and S14). In total, we detected 138 regulatory driver candidates, from which 52 were in solid cancers (5 in brain, 15 in breast, 6 in colorectal cancer, 7 in liver, 10 in lung, 6 in ovary, and 3 in pancreas) and 86 in blood cancers (Supplementary Tables S1 and S2). The target genes of the candidate drivers were more commonly upregulated (over 80% per tissue) than downregulated, and apart from blood, no recurrent mutations were detected (Figure 1B, Supplementary Tables S1 and S2).

### Target genes of non-coding regulatory driver candidates are enriched for known CDGs

Next, we sought to investigate a hypothesis that non-coding mutations may act as regulatory drivers by altering gene expression of known CDGs. Indeed, we observed a strong enrichment of CDGs within the target genes of the candidate regulatory drivers (pan-cancer: *P* = 1 × 10^−15^, fold-change 4.4×; pan-cancer solid: *P* = 1 × 10^−7^, fold-change 4.7×; Figure 2A). Interestingly, the CDG enrichment was observed across the wide range of cancer types, with the fold-change ranging between 2–7× above expectation (5/8 tissues with *P* < 0.05; Figure 2A), supporting the interpretation that positive selection rather than a specific mutational process underlies the recurrent mutations in these regions. In conclusion, the comprehensive analysis of the PCAWG dataset supports the hypothesis that regulatory regions of known cancer driver genes are enriched for non-coding driver mutations.

### Tissue specificity is critical for unravelling non-coding regulatory drivers

We next explored the impact of tissue specificity in our analysis. We compared the CDG enrichment for tissue-matched ABC maps (as in the analysis above) and tissue-unmatched ABC maps. Strikingly, in all cancer types, the strongest CDG enrichment was achieved when the tissues of the cancer and ABC enhancer data were matched (Figure 2C). These results support the importance of tissue specificity when predicting the non-coding regulatory drivers in individual cancer types.

### The importance of SNV functional impact in the regulatory driver definition

#### Impact on gene expression

We observed that some genes had a high *score_M_*, but not a high *score_E_*. Their regulatory regions may have been called non-coding drivers in the previous studies that do not consider correlation of non-coding mutations with gene expression. Interestingly, the target genes of these regions were only mildly enriched for CDGs, while the enrichment increased by 2–6-fold when both high *score_M_* and *score_E_* were required (Figure 2B). Finally, we validated that the results are not highly sensitive to the specific cut-off choice for *score_M_* and *score_E_* (Supplementary Figure S3).

#### CADD score of pathogenicity

We next explored the importance of using only SNVs with CADD (18) PHRED ≥ 10. When repeating the same analysis with different CADD cut-off values, we observed an increasing trend between the CADD cut-off and the log_2_ fold-enrichment of CDGs in the targets of the non-coding regulatory driver candidates (Pearson correlation *r* = 0.9, *P* = 6 × 10^−4^; Figure 2D, Supplementary Figures S2 and S15). While the enrichment was positive also for CADD PHRED cut-off value of 0 (i.e. all SNVs included), the strongest enrichment was observed for the highest CADD cut-offs (PHRED ≥ 18), i.e. when including only the SNVs that are predicted to be the most pathogenic ones based on the CADD score. In the following sections of the paper, we used the CADD PHRED cut-off value of 10, as a trade-off between the CDG enrichment and a sufficient statistical power (Supplementary Figure S15).

In conclusion, these results suggest that including the conditions on the functional impact of the non-coding mutations may help to distinguish between the true regulatory drivers and those that are highly mutated for other reasons, such as increased background mutagenesis not captured by the background mutagenesis model.

### A wide range of mutational processes contribute to the non-coding regulatory candidate driver mutations in solid cancers

We next compared the mutational spectra of the non-coding regulatory candidate driver mutations to COSMIC mutational signatures (30). We did not observe a strong resemblance to any single mutational signature in the solid-cancer tissues (Figure 2E), indicating that a wide range of mutational processes contribute to the observed variants, and supporting that they are under true positive selection.

**Figure 3.**
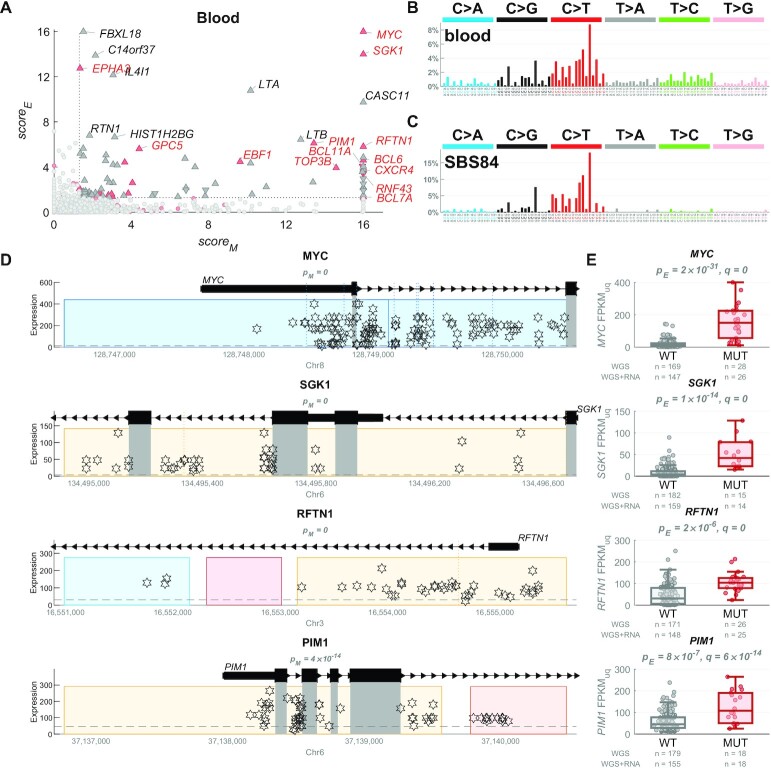
Upregulation of oncogenes in blood cancer samples with hypermutated regulatory regions by the AID-linked mutational process. (**A**) In blood (mostly lymphoma) cancer, 86 genes (triangles) have been identified as target genes of non-coding regulatory driver candidates, from which 23 genes are known CDGs (in red), with many showing extremely high *score_M_* and *score_E_* values (values above 16 are shown as 16). (**B**) The mutational profile of the non-coding regulatory candidate driver mutations in blood cancer. (**C**) The mutational profile of the COSMIC mutational signature SBS84, caused by the activation-induced cytidine deaminase (AID) activity and linked to the somatic hypermutation (SHM) in B-cells. (**D**) Examples of 4 non-coding regulatory driver candidates in blood cancers, depicting the most hypermutated regulatory regions of the given genes (all these are nearby the transcription start sites). Each star represents a mutation in one sample, with respect to its genomic position (x-axis) and expression of the target gene (y-axis). In these examples, many samples contain multiple mutations in these regions (all of these have identical values on the y-axis), in line with the AID-linked kataegis. The grey rectangles represent coding regions which are excluded from the analysis (in the entire manuscript) and any potential mutations in them are not shown here. The coloured rectangles represent the regulatory regions of the depicted gene. The *p_M_* values measure whether the regulatory space is more hypermutated than expected (*score_M_* = -log_10_(*p_M_*)). (**E**) The boxplots show the distribution of expression of the example genes in samples with mutations in the regulatory regions (in dark red) vs. the other ‘wild-type’ (WT) samples (in grey). The numbers of samples in both groups are shown in grey below the boxplots. The *p_E_* values represent the *P*-value of differential expression between the two groups, after accounting for copy number variation (*score_E_* = −log_10_(*p_E_*)) and *q* represents the Benjamini–Hochberg corrected *P*-value after combining *p_M_* and *p_E_* using the Brown's method.

In contrast, in blood, the mutational profile of the candidate regulatory driver mutations exhibited a strong resemblance to the signature SBS84 (cosine similarity 0.95, Figures 2E and 3). SBS84 is caused by activation-induced cytidine deaminase (AID) and is linked to the process of somatic hypermutation (SHM) in the immunoglobulin gene and off-target loci in B-cells (34). Many of the regulatory driver candidates in blood in our analysis exhibited remarkable upregulation of the target genes in the mutated samples, including lymphoma oncogenes, such as *MYC*, *SGK1*, *PIM1*, *BCL6*, *HIF1A* and *CD74* (Figure 3). It is possible that some of the non-coding mutations created by AID contributed to the upregulation of these oncogenes and thus represent true cancer driver non-coding mutations. Alternatively, it is possible that the genes were upregulated first, and then the active transcription may have attracted/facilitated the AID mutagenesis (34). Finally, a combination of both scenarios is also possible. The potential driver or passenger role of these mutations cannot be concluded without further computational and experimental research. In the following sections, we thus mostly focus on solid cancers only.

### Upregulation of oncogenes and downregulation of tumour-suppressor target genes of candidate non-coding regulatory drivers in solid cancers

To obtain insight into the mechanisms of the role that the non-coding driver candidates may play in solid cancers, we annotated the CDGs as known oncogenes, tumour-suppressor genes (TSGs), and other CDGs, using the Cancer Gene Census (CGC) annotations (26). We also annotated the target genes of the regulatory driver candidates as *candidate driver-upregulated genes* (upregulated in mutated samples) and *candidate driver-downregulated genes* (downregulated in mutated samples).

**Figure 4.**
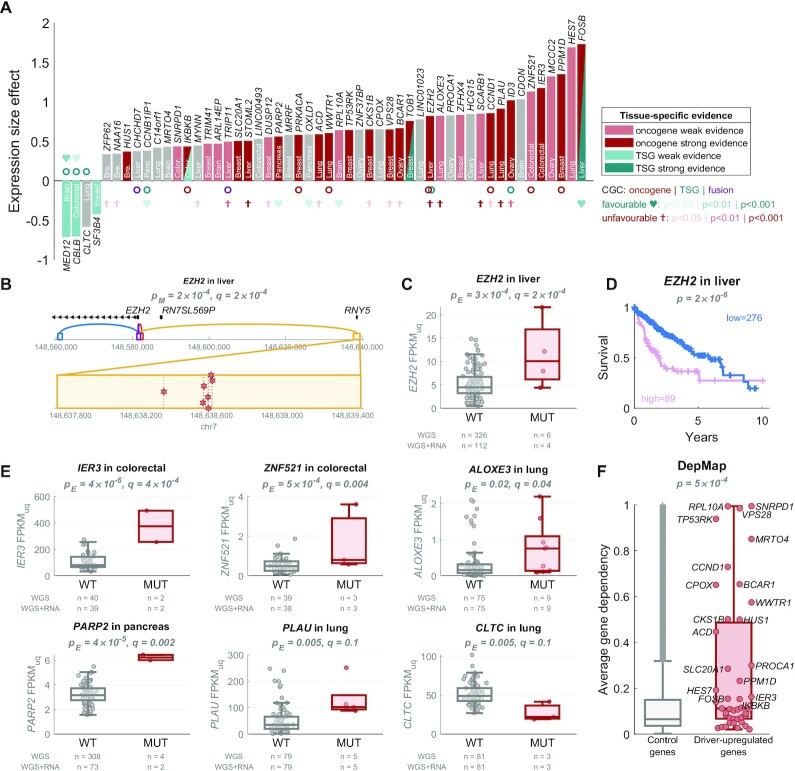
In solid cancers, oncogenes are enriched in driver-upregulated genes and TSGs are enriched in driver-downregulated genes. (**A**) The 52 target genes of the regulatory driver candidates, sorted by the size effect of their differential expression between the mutated and not mutated samples: 48 genes are driver-upregulated (positive y-axis), and 4 genes are driver-downregulated (negative y-axis). The colour of the bars represents the evidence in the literature for tissue-matched role of the genes as oncogenes (red colour) or tumour-suppressor genes (TSGs) (teal colour), with dark colour representing strong evidence (level 3 or 4) and light colour representing weak evidence (level 1 or 2; see Methods). Three genes had tissue-matched evidence in both directions (depending on phosphorylation, isoform, cellular localisation, disease stage etc.), which is denoted as two red-teal triangles. The circles below/above the bars represent the pan-cancer Cancer Gene Census (CGC) annotations: oncogenes (red), TSG (teal) or fusion (purple). The hearts and crosses represent the tissue-matched survival prognostic results based on The Human Protein Atlas (28): high expression predictive of favourable prognosis (teal hearts) or unfavourable prognosis (red crosses). The level of significance is denoted by the brightness of the colour. The y-axis (expression size effect) corresponds to the beta value of the MUT predictor in the Poisson GLM. In other words, it represents the log(FPKM-UQ) expression increase (or decrease if negative) in mutated samples, after accounting for copy-number variation. (**B**) Genomic view of an example gene *EZH2*. Four regulatory regions (ABC enhancers) are shown, each in a different colour: purple in the *EZH2* promoter, dark orange nearby the promoter, blue in intron of *EZH2*, and a yellow enhancer 59 kb upstream of *EZH2*. The yellow enhancer has seven mutations, shown as red stars in the inset below (two are in one sample). (**C**) The *EZH2* expression in samples with (red) and without (grey) mutations in the regulatory regions of *EZH2*. (**D**) The Kaplan–Meier plot of survival probability in samples with high (pink) vs. low (blue) *EZH2* expression, with the log-rank test *P*-value shown on top, data from The Human Protein Atlas (28). (**E**) Examples of expression in five driver-upregulated genes and one driver-downregulated gene. (**F**) The average dependency score across 939 cancer cell lines from the DepMap Achilles project (29) in the driver-upregulated genes in solid cancers (red) and the control genes (all genes not regulated by the regulatory driver candidates), with two-tailed Wilcoxon rank-sum test *P*-value shown on top. The dependency score represents how dependent the cell line is on the gene, i.e. how essential the gene is for viability of the cell line based on CRISRP/Cas9 screen.

We observed a significant enrichment of CGC oncogenes in the candidate driver-upregulated genes (6 observed versus 0.6 expected; Fisher's exact test *P* = 4 × 10^−5^, comparing driver-upregulated genes and exclusive oncogenes) and a significant enrichment of CGC TSGs in the candidate driver-downregulated genes (3 observed versus 0.05 expected; Fisher's exact test *P* = 1 × 10^−5^) in the solid cancers (Figure 4A). These results would be in line with non-coding candidate driver mutations leading to upregulation of oncogenes and downregulation of TSGs.

We note that the oncogene/TSG annotations from CGC are pan-cancer (not tissue-specific) and may not include the most recent literature. Thus, we have also performed an unbiased literature search for tissue-specific oncogenic or tumour-suppressive roles of each of the 48 driver-upregulated and 4 driver-downregulated genes (see Methods). Interestingly, 67% driver-upregulated genes showed at least weak evidence of oncogenic role in the matched tissue (Figure 4a, Supplementary Table S3). Strong evidence was observed in 20/48 (42%) genes, including 8/14 genes in breast (*CKS1B*, *HUS1*, *PPM1D*, *PRKACA*, *SLC20A1*, *TOB1*, *TP53RK*, *VPS28*), 2/5 genes in colon/rectum (*IER3*, *IKBKB*), 3/7 genes in liver (*EZH2*, *STOML2, FOSB*), 4/9 genes in lung (*ACD*, *CCND1*, *PLAU*, *WWTR1*), 2/6 genes in ovary (*BCAR1*, *ID3*), and 1 in pancreas (*PARP2*). Moreover, 3/4 driver-downregulated genes showed at least weak evidence of TSG role in the matched tissue (*MED12* in brain, *SF3B4* in breast, and *CBLB* in colorectal cancer), with the remaining 1 gene being classified as CGC TSG (*CLTC*).

Finally, we observed a strong enrichment of the driver-upregulated genes in cancer-essential genes in CRISPR screens of the DepMap Achilles project (29), exhibiting increased average dependency score (*P* = 5 × 10^–^^4^; Figure 4f, Supplementary Figure S1a) and increased percentage of cancer cell lines dependent on the given gene (*P* = 2 × 10^−4^; Supplementary Figure S1b), including cell lines of the matched tissue (Supplementary Figure S1a, b). In total, 24/43 (55.8%) protein-coding driver-upregulated genes were essential in at least one tissue-matched cell line (32.3% expected in median). The highest enrichment was present in the breast cancer (11/14 genes, *P* = 0.0005), where the driver-upregulated genes were more frequently essential in breast cancer cell lines compared to cell lines from other tissues (*P* = 0.006, Supplementary Figure S1c–e).

In summary, our candidate non-coding driver mutations are associated with upregulation of oncogenes and cancer-essential genes, and downregulation of TSGs.

### Post-hoc filtering of candidate regulatory drivers

We employed three strategies to indicate potential false positive candidate regulatory drivers in solid cancers based on post-hoc filtering. In the first post-hoc assessment of the candidate drivers (Supplementary Figures S4 and S5), we evaluated whether the background mutagenesis model potentially underestimates the mutation rate in enhancers in the surrounding regions. In this assessment, we identified three potential false positive hits: *HCG15* (ovary), *CPOX* (lung) and *CLTC* (lung) (see Methods for details). Interestingly, none of these three genes showed tissue-matched oncogenic/TSG evidence (Figure 4, Supplementary Table S3), in line with the possibility that these do not represent true drivers.

Second, we identified three pairs of candidate genes that share a mutated regulatory region: *TRIM41* & *ZFP62* (breast), *PARP2* & *CCNB1IP1* (pancreas), and *HES7* & *ALOXE3* (lung). The smaller *scoreE* and expression size effect within these pairs is in: *ZFP62*, *CCNB1IP1* and *ALOXE3*, and it is possible that these three genes represent false positives. Neither of the first two genes shows tissue-matched oncogenic/TSG evidence and *ALOXE3* only predicts poor prognosis in lung cancer (Figure 4, Supplementary Table S3).

Finally, we noticed that that targets with stronger cancer evidence in the literature have larger absolute expression size effect compared to targets without tissue-matched cancer evidence in the literature (*P* = 0.02, Supplementary Figure S16, Figure 4A). It is possible that targets with low absolute expression size effect represent false positive hits due to spurious signal. Using a cut-off of 1/3 quantile in targets without evidence (expression size effect 0.4326), we identified additional 8 targets as potential false positives: *MRTO4* (brain), *NAA16* (breast), *ZFP62* (breast), *SF3B4* (breast), *HUS1* (breast), *CHCHD7* (liver), *C14orf1* (lung), and *CCNB1IP1* (pancreas) (Figure 4A, Supplementary Table S2).

**Figure 5.**
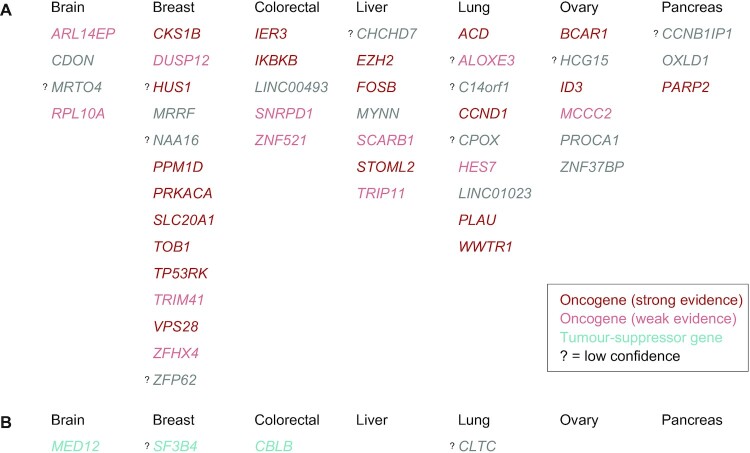
The list of 48 candidate driver-upregulated genes (**A**) and 4 candidate driver-downregulated genes (**B**) in solid cancers. Genes are grouped by the tissue and sorted alphabetically. Colour codes for the tissue-matched cancer evidence in the literature, as in Figure 4: oncogenes in red, TSGs in teal, no evidence in grey. In the case of dual evidence, only one is shown in this figure (both are indicated in Figure 4 and Supplementary Table S2). Question mark denotes target genes with low confidence, based on the three-layer post-hoc identification of potential false discoveries.

With the three post-hoc filtering strategies together, the percentage of driver-upregulated targets that have tissue-matched oncogenic literature evidence increased from 67% (32/48) to 79% (30/38) in solid cancers, as summarised in Figure 5 and Supplementary Table S2. Of note, applying the same three strategies on blood cancers identified 38/86 potential false positives (Supplementary Table S2). After their removal, the percentage of target genes in blood that are annotated as CGC CDGs increased from 27% (23/85) to 35% (17/48).

### Alteration of transcription factor binding sites by the candidate regulatory driver mutations in solid cancers

**Figure 6.**
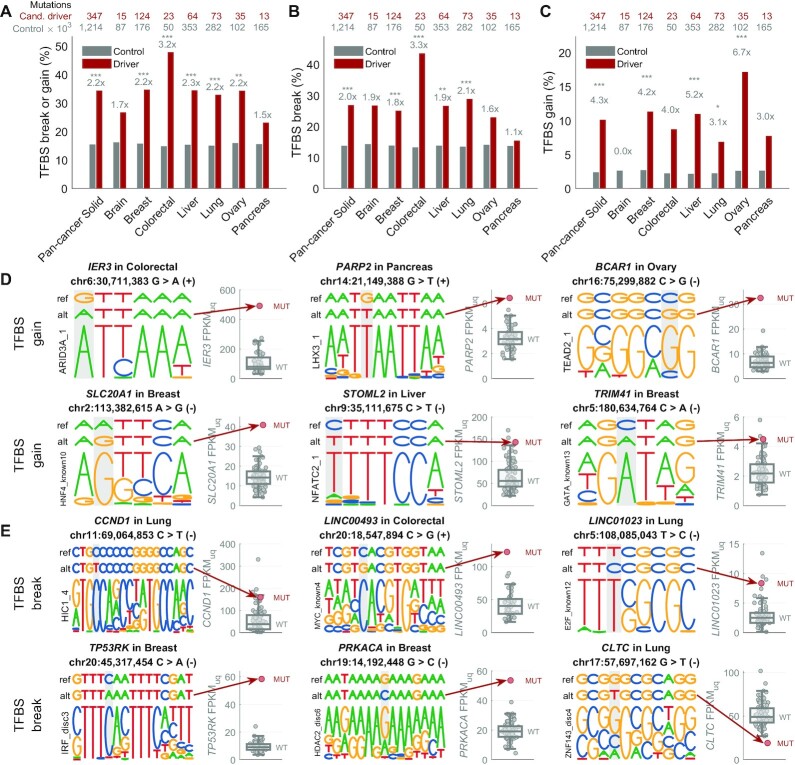
Alteration of transcription factor binding sites by the regulatory driver candidate mutations in solid cancers. (**A**) Percentage of mutations predicted to create a transcription factor binding site (TFBS) break (disruption) or gain (*de novo* TFBS) by the FunSeq2 (32) tool, shown for the non-coding regulatory candidate driver mutations (all mutations in the regulatory space of driver-upregulated and driver-downregulated genes; dark red) and the control mutations (all other mutations in the ABC enhancers; grey). The numbers above each pair of bars represent the ratio between the two bars, and the stars represent the significance level based on the two-tailed Fisher's exact test (***P < 0.001; **P < 0.01; *P < 0.05). The two rows of numbers of top of the plot represent the number of all non-coding regulatory candidate driver mutations (first row, dark red) and all control mutations (second row, grey). (**B**) As in (A), but only for TFBS breaks. (**C**) As in (A), but only for TFBS gains. (**D**) Examples of six candidate non-coding regulatory driver mutations associated with upregulation of the target gene and predicted to create a *de novo* TFBS (gain). In each example, the top row represents the reference sequence, the second row represents the mutated sequence (with the alt-allele base), and the bottom row represents the motif that is created by the mutation (the motifs are from ENCODE-motifs). The mutated position is highlighted by grey background. The boxplot on the right-hand side of each example shows the distribution of the target gene expression in the wild-type samples, while the red circle represents the expression level in the sample with the depicted mutation. (**E**) As in (D), but showing TFBS break examples in regulatory regions of driver-upregulated genes (five examples) and driver-downregulated genes (one example).

We observed a significant alteration of transcription factor binding sites (TFBS) by the candidate regulatory driver mutations in comparison with other mutations inside regulatory regions, as predicted by FunSeq2 (32) (Figure 6 including all-CADD mutations, Supplementary Figure S17 comparing low-CADD and high-CADD mutations). Over 34% of candidate driver mutations in the solid cancers were predicted to create a TFBS motif change (2.2-fold enrichment compared to the control mutations, *P* = 3 × 10^−18^, Figure 6A), with 27% predicted to disrupt a TFBS motif (2-fold enrichment, *P* = 1 × 10^−10^, Figure 6B–E) and 10% predicted to create a *de novo* TFBS motif (4.3-fold enrichment; *P* = 1 × 10^−12^, Figure 6C, D).

Our data are in line with two modes of how non-coding regulatory driver mutations can lead to higher expression of the target genes: (a) breaking a TFBS of a transcriptional repressor, and (b) creating a TFBS of a transcriptional activator. For example, in the first mode, a driver-upregulating mutation in the regulatory region of *CCND1* (Cyclin D1) is predicted to break binding of HIC1, a transcriptional repressor TF and a candidate tumour suppressor gene (Figure 6E). In fact, it has been shown HIC1 is a direct transcriptional repressor of Cyclin D1 (35,36). Our data are in line with a scenario where a non-coding regulatory driver mutation in the HIC1 binding site in a *CCND1* regulatory region disrupts the HIC1 binding, leading to overexpression of *CCND1*.

In the second mode, driver-upregulating mutations are predicted to create novel TFBS of transcriptional activators, such as novel ARID3A TFBS leading to upregulation of *IER3* in colorectal cancer, LHX3 TFBS leading to upregulation of *PARP2* in pancreas, TAED2 TFBS leading to upregulation of *BCAR1* in ovary, and TCF12 TFBS leading to upregulation of *PPM1D* in breast cancer (Figure 6D).

### Importance of utilizing 3D interactions as opposed to the closest-gene approach

**Figure 7.**
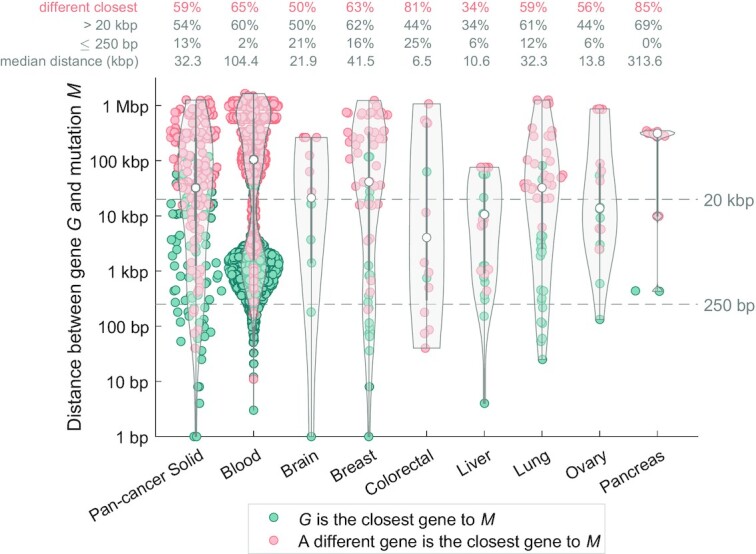
Long-range interactions are involved in the majority of non-coding regulatory driver candidates. (**A**) Each dot represents one mutation-gene pair (mutation *M* and a gene *G*), for all the non-coding regulatory driver candidates (52 genes in solid cancers and 86 genes in blood cancer). The violin plots show the distribution of distance from the mutation *M* to the transcription start site (TSS) of the gene *G*. Only high-CADD mutations in the regulatory space of gene *G* are considered. One mutation can be present in more than one pair. The pairs are colour-coded according to whether the gene *G* in the pair is the closest gene to the mutation *M* (teal colour), or if a different protein-coding gene is closer to the mutation *M* (salmon colour), using the distance to the TSS of the gene. The four rows of numbers on top of the plot represent the average values across the group and the median *M–G* distance (kb).

In solid cancers, only 13% of the regulatory driver mutations were within 250 bp of the transcription start site (TSS) of their differentially expressed target gene and 54% were >20 kb distant (median distance 32.3 kb; Figure 7, Supplementary Table S4). Strikingly, for 59% regulatory driver mutations-gene pairs, the differentially expressed target gene would have been missed if the closest protein-coding-TSS assignment was used (67% mutations for the closest TSS of any gene; Supplementary Figure S6), showing the importance of 3D interactions in determining non-coding regulatory drivers.

### Candidate AID-generated regulatory cancer driver mutations in blood

As discussed above, the candidate driver mutations in blood are largely due to AID-mediated mutational process responsible for mutational signature SBS84. It is thus possible that many of those mutations are not *bona fide* cancer drivers. On the other hand, the AID-mediated mutagenesis creates a large number of mutations in regulatory regions of important lymphoma oncogenes, increasing the chance that some of these mutations may confer selective advantage to those cells, e.g. by altering TFBS in the regulatory regions, leading to upregulation of the oncogenes. To investigate this possibility, we explored TFBS alterations in recurrent candidate regulatory driver mutations in blood. To prevent potential artefactual results due to expression differences between different blood cancer types, only Diffuse Large B-Cell Lymphoma (DLBCL) patients were included in this analysis. Re-running the entire analysis on DLBCL samples resulted in 51 candidate target genes, including 13 known cancer driver genes (enrichment 3.5×, *P* = 5 × 10^−5^, Supplementary Figures S18 and S19). The vast majority of the genes (48/51) were driver-upregulated, including CGC oncogenes *MYC*, *BCL2*, *PIM1*, *SGK1*, *HIF1A* and *ETV6*.

**Figure 8.**
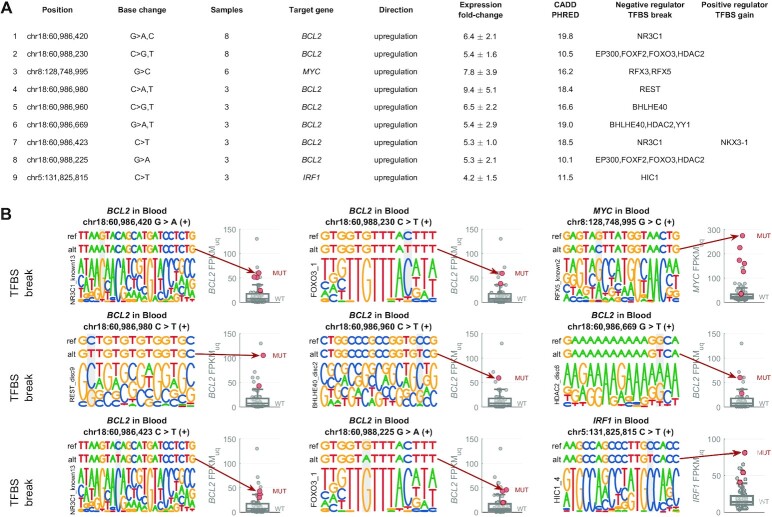
List of top 9 mutated positions with potential to act as non-coding regulatory drivers in DLBCL via disruption of transcriptional repressor TFBS. (**A**) The list of the 9 mutated positions (hg19), the base change (alternative allele), number of DLBCL samples with this mutation, the target gene they are predicted to regulate, the direction of the effect (all upregulated in mutated samples here), average and standard deviation of the expression fold-change between the mutated samples and median expression of the wild-type samples (FPKM-UQ), average CADD PHRED value, negative regulator TFs predicted to have binding disrupted by the mutation, and positive regulator TFs predicted to have binding created by the mutation. (**B**) Genomic visualisation of the 9 top hits. In each example, the top row represents the reference sequence, the second row represents the mutated sequence (with the alt-allele base), and the bottom row represents the motif that is disrupted by the mutation (the motifs are from ENCODE-motifs). The mutated position is highlighted by grey background. The boxplot on the right-hand side of each example shows the distribution of the target gene expression in the wild-type samples, while the red circles represent the expression level in the samples with the depicted mutation.

Next, we identified a list of ‘top hits’ representing regulatory mutations that are most likely to serve as the potential cancer driver mutations (see Methods for details). Using this approach, 9 top hits were predicted to disrupt TFBS of a negative regulator of transcription and associated with upregulation of their target gene (Figure 8). These hits occurred in regulatory regions of *BCL2* (7×: disrupting motifs of NR3C1, FOXO3, REST, BHLHE40, HDAC2 and other TFs), *MYC* (1×: disrupting RFX3/5 motif), and *IRF1* (1×: disrupting HIC1 motif). Moreover, additional mutations were predicted to disrupt other positions of the same TFBSs (Supplementary Figure S19 and S20), explaining a large proportion of the upregulated mutated samples in the respective target genes (21/38 in *BCL2*, 9/19 in *MYC*, 2/2 in *IRF*).

Finally, we identified 9 top hits predicted to create *de novo* TFBS of a positive regulator of transcription and associated with upregulation of their target genes *BCL2, HIST1H3J*, and *SGK1* (Supplementary Figure S21). Additional non-recurrent examples (concerning *MYC*, *PIM1*, *EBF1*, *SGK1*, *IRF1* and *BCL2*) are shown in Supplementary Figure S22).

In conclusion, many of the recurrent regulatory mutations in DLBCL alter TFBS and may serve as regulatory cancer drivers. However, any conclusions about the regulatory cancer drivers in DLBCL cannot be drawn without experimental validation.

## DISCUSSION

Since the discovery of the non-coding driver mutations in the *TERT* promoter (37,38), the field of cancer genomics has shown a great interest in searching for ‘the other TERTs’. While many of the focused analyses brought intriguing and promising findings (2,4,39–42), a key detailed collaborative pan-cancer analysis by the PCAWG Consortium yielded unexpectedly few non-coding drivers and raised doubts about some of the previously identified drivers (3).

Here, we designed a novel approach to comprehensively identify candidate non-coding driver mutations in *cis*-regulatory elements acting by altering the expression of the genes they regulate. We employed a strategy with several key differences compared to the previous studies, focusing only on tissue-specific *cis*-regulatory elements, employing a gene-level analysis based on 3D interactome data, and searching only for drivers predicted to have high impact by the CADD score and to regulate expression of the given gene. Moreover, we utilised a tissue-specific model of background mutagenesis in the regulatory regions, implementing the critical features identified by Elliot and Larsson (2): accounting for key covariates, local mutation rate, trinucleotide composition, functional impact, and to some extent localised phenomena. When applied on solid cancer types from seven tissues, the method identified candidate driver mutations predicted to cause upregulation in 48 genes and downregulation in four genes. We observed a strong enrichment of known CDGs, cancer-essential genes and other genes previously implicated in cancer, within the target genes of the candidate regulatory drivers. In particular, the candidate driver mutations were predicted to lead to alteration of TFBS and contribute to upregulation of oncogenes and cancer-essential genes and to downregulation of tumour-suppressor in a tissue-specific manner.

For instance, we observed upregulation of *EZH2* in liver cancer samples with a mutation in the predicted distal enhancers of the *EZH2* gene, which is known to promote hepatocellular carcinoma (HCC) progression and metastasis (43,44) and its inhibitors are currently being tested in clinical trials (45). Other examples of genes upregulated in the mutated samples include: (i) *STOML2* (liver), which promotes colony formation, migration and invasion of HCC cells (46) and its downregulation leads to increased sensitivity to lenvatinib (47), (ii) *PLAU* (lung), which is a key component of the long-known oncogenic plasminogen activator system important in lung tumour progression, proliferation and metastasis (48), is being tested as a drug target (49), and is overexpressed in progressive lung cancer (50), (iii) *CCND1* (lung), a druggable gene (51) known to promote lung cancer growth and metastasis (52), (iv) *IER1* (colorectal cancer), the deficiency of which protects against colorectal cancer in mice (53), (v) *IKBKB* (colorectal cancer), which promotes carcinogenesis via activation of Wnt signalling and production of pro-inflammatory intestinal microenvironment (54), (vi) *PARP2* (pancreas), an important therapeutic target (55), (vii) *BCAR1* (p130Cas; in ovary) a well-known oncogene in ovarian cancer (56), which confers resistance to anti-angiogenesis therapy in ovarian tumours (57), (viii) *PRKACA* (breast), an anti-apoptotic oncogene mediating resistance to HER2-targeted breast-cancer therapy (58), (ix) *CKS1B* (breast), anti-apoptotic pro-invasive oncogene in breast cancer (59) and a drug resistance-inducing gene (60), and many others (Supplementary Table S3).

While the majority of the target genes of the non-coding regulatory drivers predicted by our analysis are protein-coding genes, two of the high-confidence solid-cancer hits are lncRNAs: *LINC01023* (lung cancer) and *LINC00493* (colorectal cancer). The candidate regulatory driver mutations are predicted to alter TF binding in all the mutated samples and cause upregulation of the lncRNA genes. Interestingly, oncogenic role of LINC01023 has been described in liver and brain cancers (61,62) (Supplementary Table S3). Our results predict that oncogenicity of LINC01023 extends also to lung cancer, expanding the previously described role of driver mutations acting through lncRNAs and other ncRNAs (63). More work on the role of non-coding RNA in cancer is needed to better understand the contribution of these important biological elements to cancer.

In line with previous studies, our results show that enhancer mutagenesis dramatically differs between the blood and solid cancers. This difference is largely driven by an intrinsic biological mutational process linked to somatic hypermutation (SHM) and activation-induced cytidine deaminase (AID). This process occurs in antigen-activated germinal centre B cells and provides the molecular basis for affinity maturation of antibodies (64). At the same time, off-target AID-mediated SHM can produce mutations driving lymphoid cancer malignancies (65). Some of these target and off-target regions carry a ‘storm of mutations’ termed *kataegis*, comprising many mutations in one sample within a small region. Importantly, off-target AID-mediated SHM is known to be enriched in enhancers, super-enhancers, near transcription start sites, and generally linked to active transcription (34). The reasons for this enrichment are not fully understood but may be linked to higher accessibility of single-stranded DNA during active transcription and the fact that AID deaminates cytosines in single-stranded DNA (34). Our results expand the previous literature, highlighting the scale of AID-mediated enhancer mutagenesis in blood (including enhancers of 23 CGC cancer driver genes) associated with transcription (positive association in 84%).

The fact that AID mutagenesis targets specifically regulatory regions and seems to be mechanistically linked to active transcription limits our ability to distinguish between true driver vs. passenger mutations in these regulatory regions. By looking at TFBS alterations, mutation recurrence, and other features, we have constructed a list of mutations that could act as regulatory cancer drivers. Since the first submission of our manuscript, a study by Bal *et al.* published in *Nature* showed that some of the AID-generated mutations can indeed lead to oncogene downregulation via disruption of transcriptional repressors (66). Strikingly, one of the mutations experimentally validated by Bal *et al.* is the top position in our list (Figure 8). In our analysis, the position 60 986 420 in chromosome 18 (hg19 coordinates) was the most recurrent high-CADD regulatory driver mutation (affecting 8 samples), associated with an average 6-fold upregulation of the target gene *BCL2*, and predicted to disrupt binding of transcriptional repressor NR3C1, leading to upregulation of *BCL2*. Bal *et al.* showed that CRISPR–Cas9-mediated correction of this mutation in LY10 DLBCL cell line leads to decreased *BCL2* mRNA expression and dramatically decreased cell growth and survival, revealing oncogenic addiction of the cell-line to this mutation. On the other hand, introduction of this mutation into *BCL2*-negative cell lines was sufficient to reactivate *BCL2* expression. Using ChIP–qPCR, Bal et al. showed that NR3C1 binds the predicted motif, and this binding is largely disrupted in the mutated alleles of the LY10 cell line. These experimental results thus confirm that this mutation prevents the transcriptional repression of *BCL2* by NR3C1. Our results predict that *BCL2* upregulation can result also from other mutations in the NR3C1 binding site, as well as five binding sites of other transcriptional repressors. Moreover, our analysis suggests that also other oncogenes, such as *MYC*, may be upregulated by non-coding regulatory drivers in DLBCL.

Cancer results from selection of cells with mutations that lead to increased survival, proliferation, tissue invasion, and spread to other organs (67). Detecting positive selection is therefore one of the key steps in deciphering which mutations are responsible for cancer initiation and progression. Regions under positive selection carry recurrent mutations across cancer patients, with observed mutation density exceeding the expected density of neutral mutations in that region. However, modelling the expected density is hard due to non-random distribution of the background damage and subsequent repair. Regions frequently exposed to damage or difficult to repair may get frequently mutated without providing any selective advantage to the cell (8). Our study shows several methodological elements that help to detect regulatory regions under positive selection in the non-coding genome: (a) searching for mutations that are associated with upregulation or downregulation of the gene they regulate, (b) searching for regulatory mutations that may be distant in the linear genomic distance but close in the 3D space, (c) tissue-matched enhancer-gene maps using the Activity-by-Contact model, (d) filtering by mutation pathogenicity predictions by the CADD method, and (e) using a complex tissue-specific model of background mutagenesis.

In conclusion, our results have implications for the importance of tissue-matched long-range chromatin interactions and of the functional readout requirements in the search for non-coding cancer drivers. Our findings demonstrate that the signal of non-coding drivers is spread over multiple regions and potentially large genomic distances close in the 3D space and that there is little recurrence in individual positions. The statistical power seems to be currently the major limiting factor, and we expect that more matched WGS and RNA-seq samples will lead to increased confidence in the identified regulatory drivers and identification of many more novel regulatory drivers.

The reported candidate cancer drivers found using our novel approach are supported by multiple independent lines of clinical, experimental, statistical, and bioinformatics evidence: (a) recurrent occurrence of mutations in the predicted drivers in cancer patients, in line with positive selection (*score_M_* and *p_M_* in the Methods and Figures 1, 3, 4; based on datasets from (6,18,19)), (b) functional impact of mutations on gene expression (*score_E_* and *p_E_* in the Methods and Figures 1, 3, 4; data from (6,18,19)), (c) enrichment of cancer driver genes in the targets of the non-coding driver candidates (Figures 1B, 2A–D, 3A, 4A; data from (26)), (d) tissue-specificity of this enrichment (Figure 2C; data from (6,26)), (e) tissue-matched experimental evidence that the driver-upregulated genes act as oncogenes and driver-downregulated genes act as TSGs in the respective tissues (Figures 4A and 5; based on data summarised in Supplementary Table S3 and (28)), (f) enrichment of cancer-essential genes in the driver-upregulated genes (Figure 4F, Supplementary Figure S1, data from (29)), (g) significant alteration of TFBS by the predicted driver variants (Figure 6, Supplementary Figures S16 and S17, 20–22, data from (32,33)), (h) many independent validations of the different parts of the framework (Figure 2B–E, Supplementary Figures S2-S14) and (i) experimental validation of the top hit prediction (i.e. the driver mutation in promoter of BCL2) (Figure 8, Supplementary Figures S19 and S20; data from (32,33,66)). We expect that future wet-lab experiments will help to functionally characterise the role of the other individual predicted non-coding regulatory drivers in cancer development.

More broadly, this study advances understanding of the role of non-coding genome in carcinogenesis, suggests mechanisms why some mutations may be pathogenic/carcinogenic when present in one tissue but not another, and provides methodology to detect candidate driver mutations that can be further investigated experimentally and ultimately for clinical translation into precision medicine.

## DATA AVAILABILITY

All data used in this study were obtained from publicly available sources and are described in detail in Supplementary Table S5. The code is available at https://github.com/tomkovam/Dr.NOD and https://doi.org/10.6084/m9.figshare.21728294 (Dr.Nod stands for Discovery of Regulatory NOn-coding Drivers). The used toolboxes and libraries are listed in Supplementary Note S1.

## Supplementary Material

gkac1251_Supplemental_Files
