# Supplementary material for: Dr.Nod: computational framework for discovery of regulatory non-coding drivers in tissue-matched distal regulatory elements
Source: Nucleic Acids Res. 2023 Jan 10;51(4):e23. doi: 10.1093/nar/gkac1251 (PMC9976879; doi:10.1093/nar/gkac1251)
Supplement: gkac1251_Supplemental_Files [file gkac1251_supplemental_files.zip › SupplementaryMaterials_revised2.pdf]

Supplementary Materials for

Dr.Nod: computational framework for Discovery of Regulatory NOn-coding Drivers in tissue-matched distal regulatory elements

Marketa Tomkova[1,2]*, Jakub Tomek[3], Julie Chow[1], John D. McPherson[1], David J. Segal[1], Fereydoun Hormozdiari[1,4,5]*

[1] Department of Biochemistry and Molecular Medicine, University of California, Davis, CA 95616, USA

[2] Ludwig Cancer Research, University of Oxford, Oxford, OX3 7DQ, UK

[3] Department of Pharmacology, University of California, Davis, CA 95616, USA

[4] UC Davis Genome Center, University of California, Davis, CA 95616, USA

[5] UC Davis MIND Institute, University of California, Davis, CA 95616, USA

* Co-corresponding authors:

- Fereydoun Hormozdiari
  fhormozd@ucdavis.edu
- Marketa Tomkova
  mtomkova@ucdavis.edu

  Department of Biochemistry and Molecular Medicine
  UC Davis Genome Center
  University of California, Davis
  CA 95616, USA

Content

1. Supplementary Figures
2. Supplementary Table Legends
3. Supplementary Note 1: Code Dependencies

Supplementary Figures

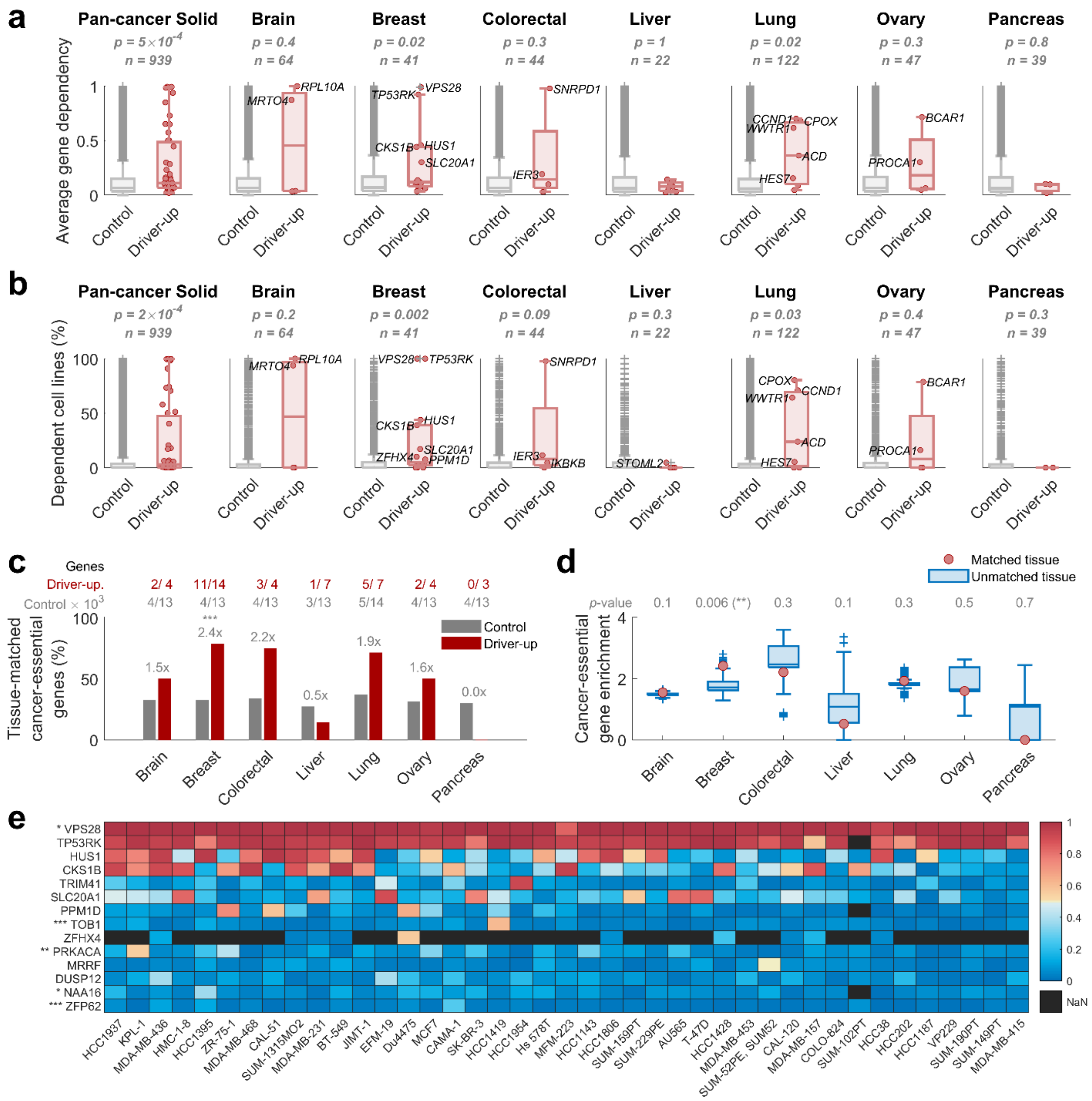

Supplementary Fig. 1 | Cancer-essential genes from the DepMap Achilles project are enriched in the driver-upregulated genes. a, The average dependency score in cancer cell lines from the DepMap Achilles project in the driver-upregulated genes (red) and the control genes (all genes not regulated by the regulatory driver candidates), with two-tailed Wilcoxon rank-sum test p-value and *n* = the number of used cell lines shown on top. The dependency score represents how dependent the cell line is on the gene, i.e., how essential the gene is for viability of the cell line based on CRISRP/Cas9 screen. The first plot represents pan-cancer analysis for all 48 driver-upregulated genes in solid cancers and the y-axis value is computed as the average across all the 939 cancer cell lines. The following seven plots show the tissue-specific results, using only cell lines and driver-upregulated genes of the given tissue. **b,** As (**a**), but the y-axis showing the percentage of cell lines dependent on the given gene (i.e., in which the gene is essential) defined as cell lines with dependency score > 0.5. **c,** The percentage of genes with at least one tissue-matched dependent cell line (cell line, where the gene is essential, defined as having dependency score > 0.5). The red bars represent driver-upregulated genes. The grey bars represent the control genes (all genes not regulated by the regulatory driver candidates in any tissue). The numbers above each pair of

bars represent the ratio between the two bars, and the stars represent the significance level based on the two-tailed Fisher's exact test (***p<0.001; **p< 0.01; *p< 0.05). The results show that in 5/7 tissues, the driver-upregulated genes have a positive enrichment for tissue-matched cancer essential genes (significant enrichment in breast cancer). **d,** Analysis of tissue-specificity of the results in (**c**). For every tissue, we selected a subset of k tissue-unmatched cell lines, where k is number of tissue-matched cell lines (used in **c**), and computed the fold-change enrichment, all over 10,000 iterations. The distributions of the resulting tissue-unmatched enrichment values are shown in the blue boxplots. The red circle represents the enrichment when the tissue-matched cell lines are used (as in (**c**)). The p-value at the top of the plot represents the proportion of the distribution being more extreme than the tissue-matched value (computed as 2x of the lower one-tailed p-values). The figure shows that the driver-upregulated breast cancer genes are enriched in cancer-essential genes and this enrichment is significantly higher when using tissue-matched vs. tissue-unmatched genes. The same trend (but not significant) is observed in brain and lung. In colorectal cancer and ovary, the enrichment is high in both tissue-matched and tissue-unmatched cell lines, while in liver and pancreas the tissue-matched enrichment is lower than expected (not significant difference). **e,** The heatmap shows the dependency score values for the 14 driver-upregulated genes in breast cancer (rows) and the 41 breast cancer cell lines (columns). The black colour represents cell lines with ≤ 1 transcript per million (TPM) for the given gene and cell line. Genes with increased dependency score in breast cancer cell lines compared to other cell lines (measured by two-tailed Wilcoxon rank-sum test) are denoted by stars next to the name (***p<0.001; **p< 0.01; *p< 0.05).

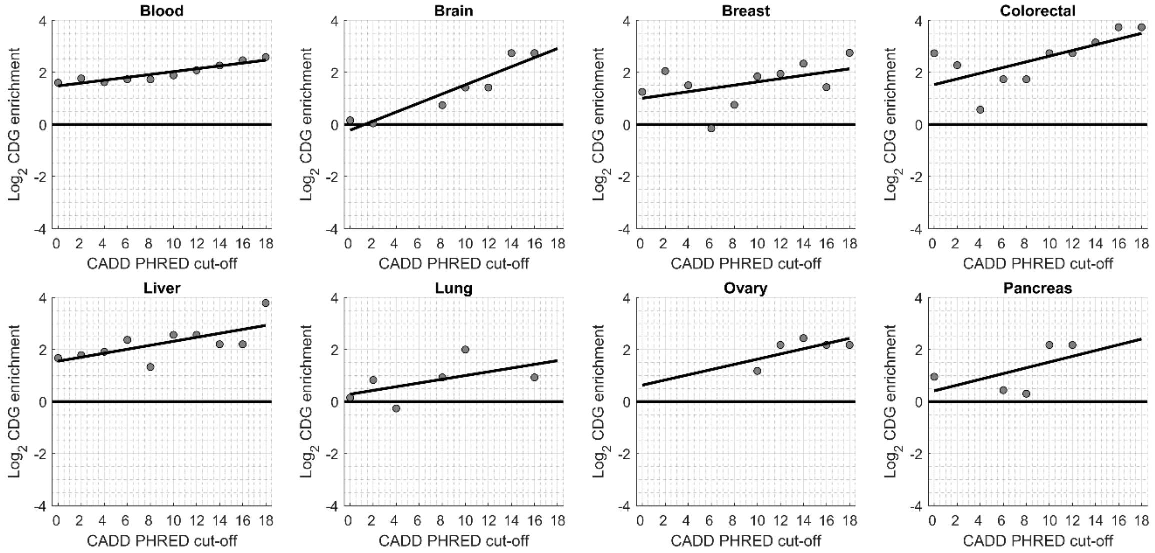

Supplementary Fig. 2 | CDG enrichment in non-coding regulatory driver targets increases with pathogenicity of the regulatory SNVs as defined by CADD score. The $\log_2$ fold-change CDG enrichment (y-axis) when different cut-off values of the CADD PHRED score (x-axis) are used. A line was fitted through the data points in each tissue.

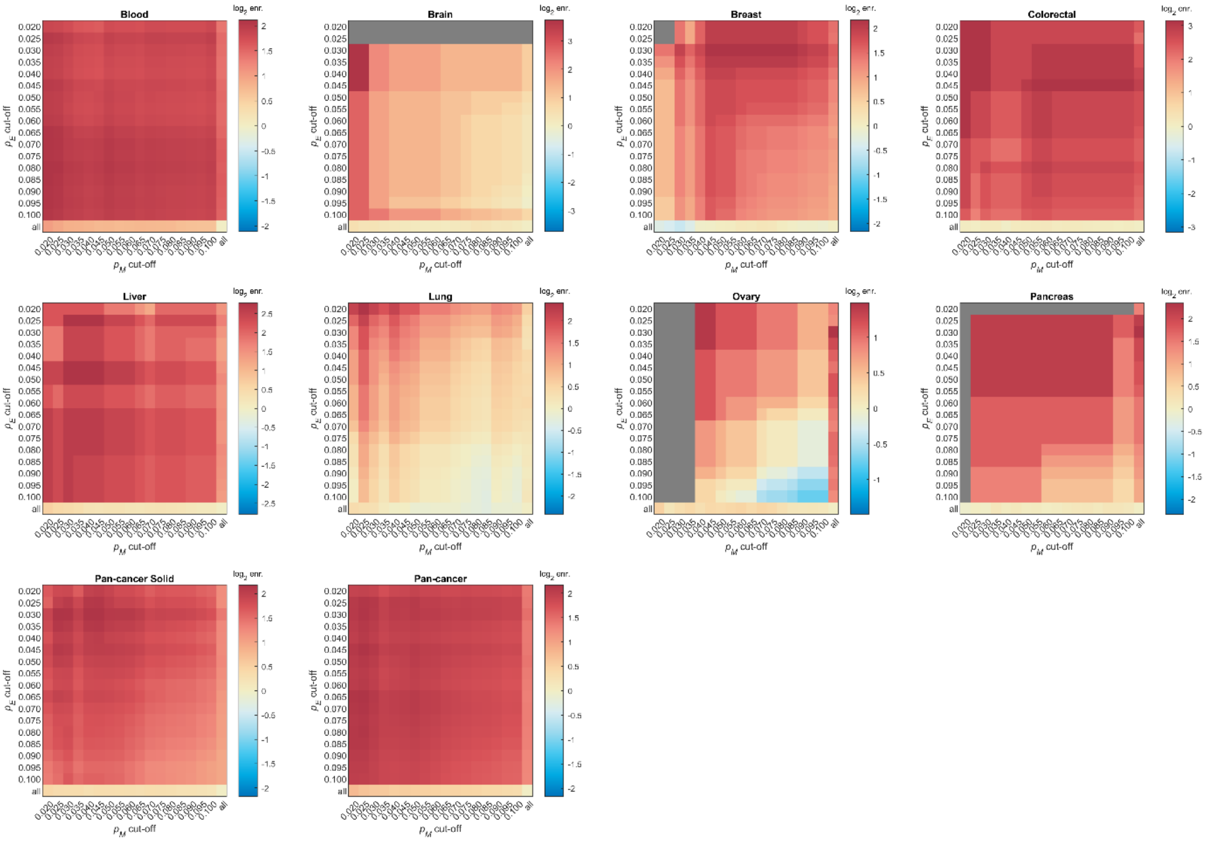

Supplementary Fig. 3 | Robustness analysis of the cut-off values. a, The heatmaps show the $\log_2$ fold-change CDG enrichment when the regulatory driver candidates are defined as $p_M < p_M$ cut-off & $p_E < p_E$ cut-off for a range of the cut-off values. In this analysis, the condition on combined q-value was not used (as it would complicate interpretation of the results). This analysis shows that the results are not sensitive to the choice of the cut-off values. In fact, the CDG enrichment generally increases with the stringency of both cut-offs, in line with the importance of both conditions in the definition. At the same time, more stringent cut-off result in lower statistical power. In the rest of the study, the cut-offs of 0.05 were used as a trade-off between the CDG enrichment and statistical power.

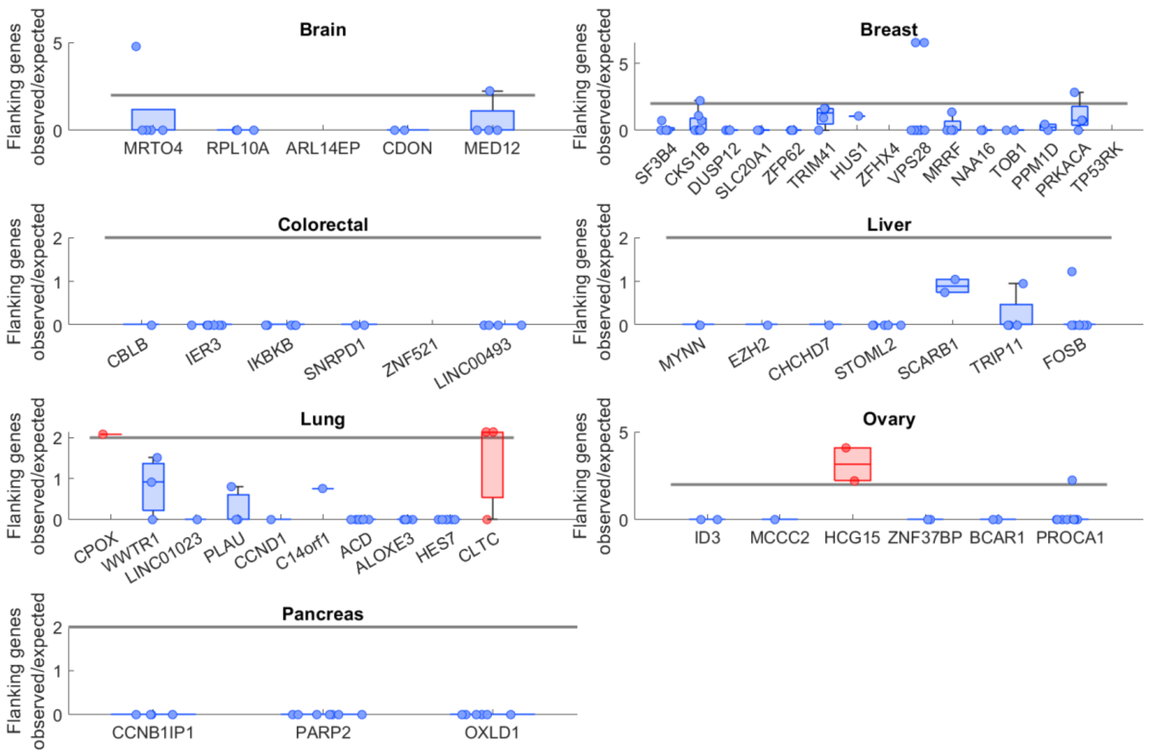

Supplementary Fig. 4 | Post-hoc analysis of potential false positive candidate drivers in solid cancers. Here, we evaluated whether the background mutagenesis model potentially underestimates the mutation rate in enhancers in the surrounding regions. The x-axis represents the 52 regulatory driver candidates in solid cancers. Each point represents one of the neighbouring genes (in distance up to 100 kb), and the y-axis value represents the observed/expected mutations in its regulatory space, excluding mutations shared with the evaluated regulatory driver candidate. Then regulatory driver candidates with median value above 2x are considered as potential false positives (shown in red), as the background mutagenesis model may have falsely underestimated the mutation frequency in this region.

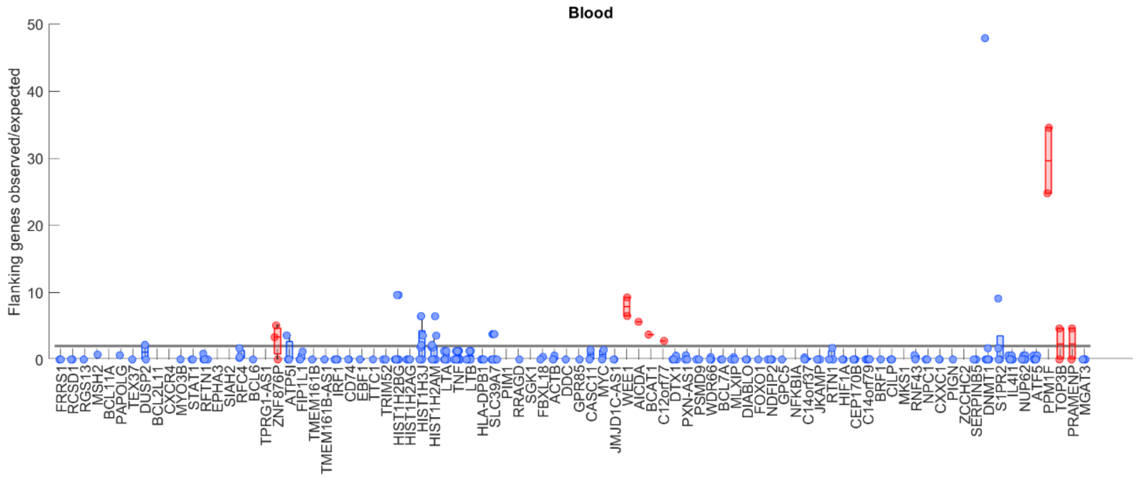

Supplementary Fig. 5 | Post-hoc analysis of potential false positive candidate drivers in blood cancers. The x-axis represents the 52 regulatory driver candidates in solid cancers. Each point represents one of the neighbouring genes (in distance up to 100 kb), and the y-axis value represents the observed/expected

mutations in its regulatory space, excluding mutations shared with the evaluated regulatory driver candidate. Then regulatory driver candidates with median value above 2x are considered as potential false positives (shown in red), as the background mutagenesis model may have falsely underestimated the mutation frequency in this region.

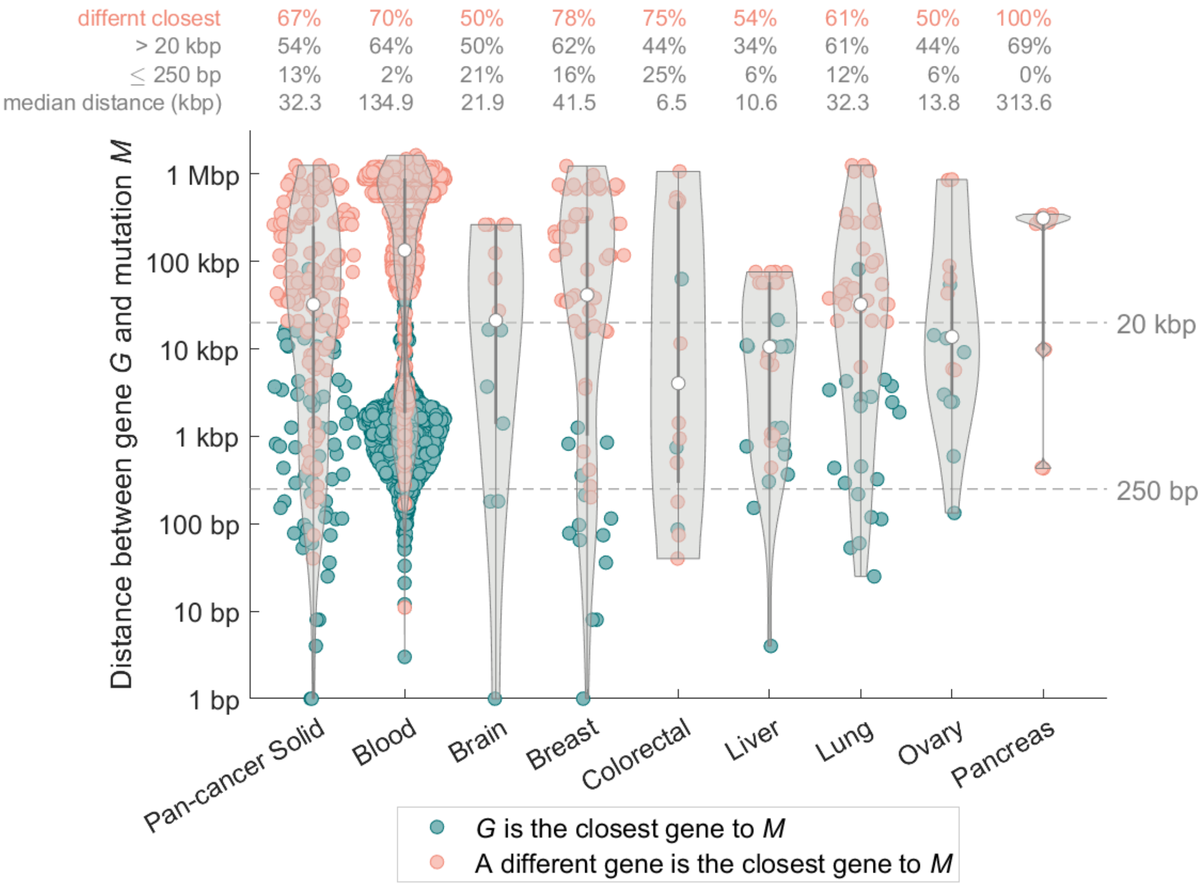

Supplementary Fig. 6 | Long-range interactions are involved in the majority of non-coding regulatory driver candidates. As Fig. 7 but taking all genes into account when searching for the closest gene. Each dot represents one mutation-gene pair (mutation *M* and a gene *G*), for all the non-coding regulatory driver candidates (52 genes in solid cancers and 86 genes in blood cancer). The violin plots show the distribution of distance from the mutation *M* to the transcription start site (TSS) of the gene *G*. Only high-CADD mutations in the regulatory space of gene *G* are considered. One mutation can be present in more than one pair. The pairs are colour-coded according to whether the gene *G* in the pair is the closest gene to the mutation *M* (teal colour), or if a different gene is closer to the mutation *M* (salmon colour), using the distance to the TSS of the gene. Both protein-coding and other genes were considered here. The four rows of numbers on top of the plot represent the average values across the group and the median *M*-*G* distance (kb).

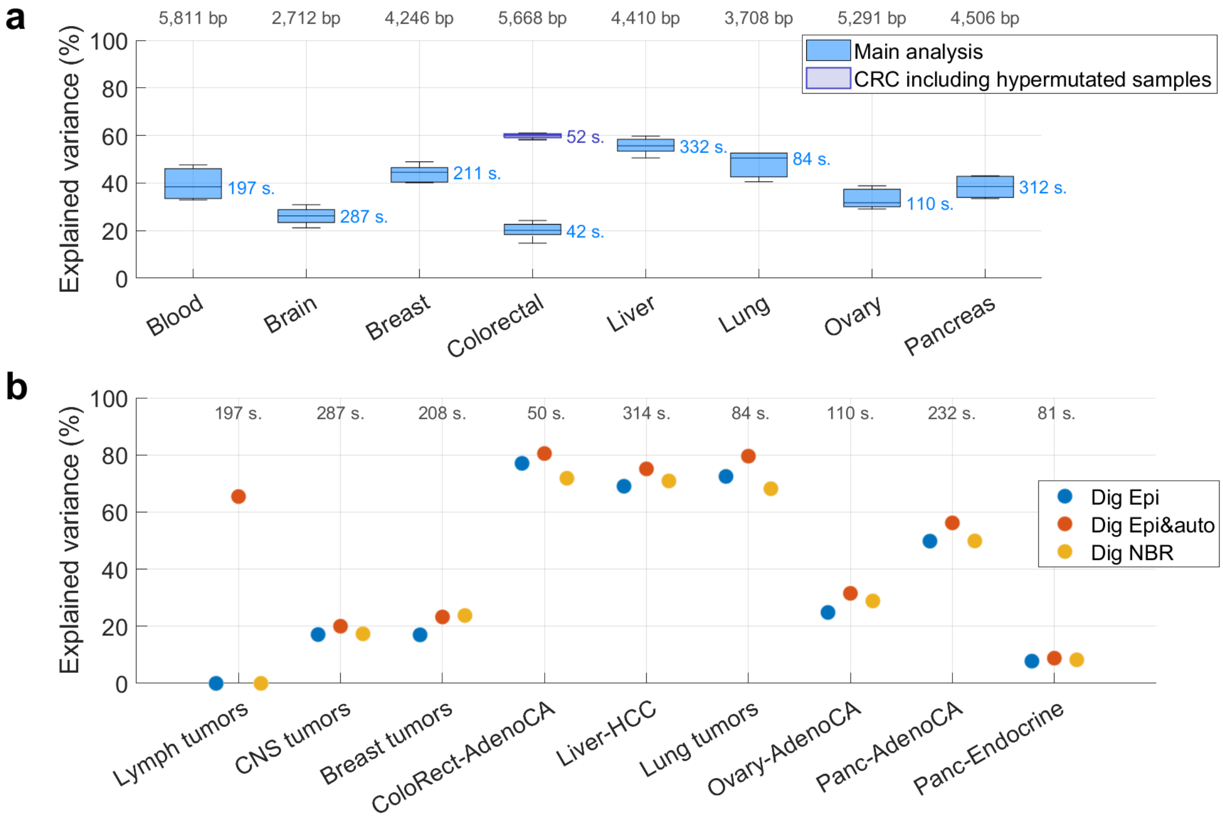

Supplementary Fig. 7 | Cross-validation evaluation of the background mutagenesis model (gene-level, all-CADD). a, The boxplots show distribution of the explained variance across the 6 folds of the cross-validation. In every fold, the model was trained on $5/6^{th}$ of the data and the remaining $1/6^{th}$ was used for evaluation. The explained variance represents the square of the Pearson correlation coefficient of the predicted and observed mutation counts in the non-coding regulatory space of that gene. The median size of the regulatory space is shown in grey in the top row. The number of used samples in each tissue are shown in blue on the right-hand side of each box. In colorectal cancer (CRC), a version with all 52 samples included (i.e., not excluding the hypermutated samples) is shown in violet, in order to enable better comparison with the previously published models shown in (b). All SNVs were used to train and evaluate the models in this figure (to enable comparison with b). **b,** Performance of a recently published model called Dig (Sherman et al., 2022), with values from Supplementary Table 4 plotted here (a comparison of three versions of the model). The explained variance represents the square of the Pearson correlation coefficient of the predicted and observed mutation counts in 10-kb windows (which is slightly larger than the median size in our analysis in (a), which ranges between 2,712 bp to 5,811 bp). Note, that the considering larger window size indeed will improve the explained variance of any approach. Similarly, as in (a), the WGS PCAWG samples were used to train and evaluate the model. The actual regions/windows are different, and therefore the comparison is only approximate.

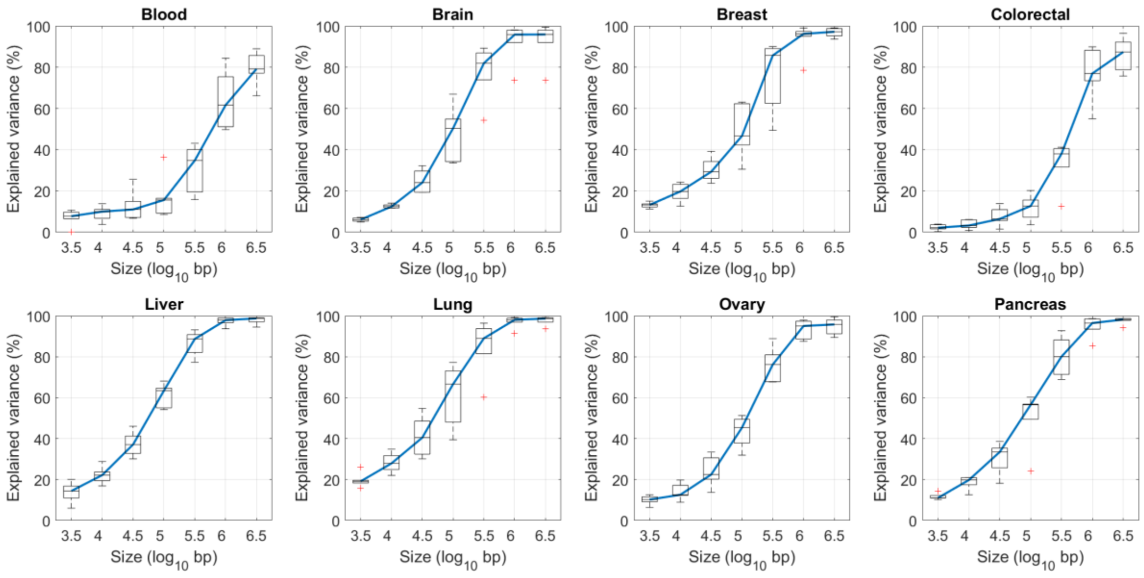

Supplementary Fig. 8 | Cross-validation evaluation of the background mutagenesis model (window-level, all-CADD). The boxplots show distribution of the explained variance across the 6 folds of the cross-validation. In every fold, a gene-level model was trained on 5/6th of the data and the remaining 1/6th was used for evaluation. The y-axis (explained variance) represents the square of the Pearson correlation coefficient of the predicted and observed mutation counts in sliding windows (neighbouring non-coding regulatory regions grouped together by size). The total size of each window is shown on the x-axis on a log_{10}-scale. The predictors in each window were computed as average of the predictors across all the included regulatory regions. All SNVs were used to train and evaluate the models in this figure.

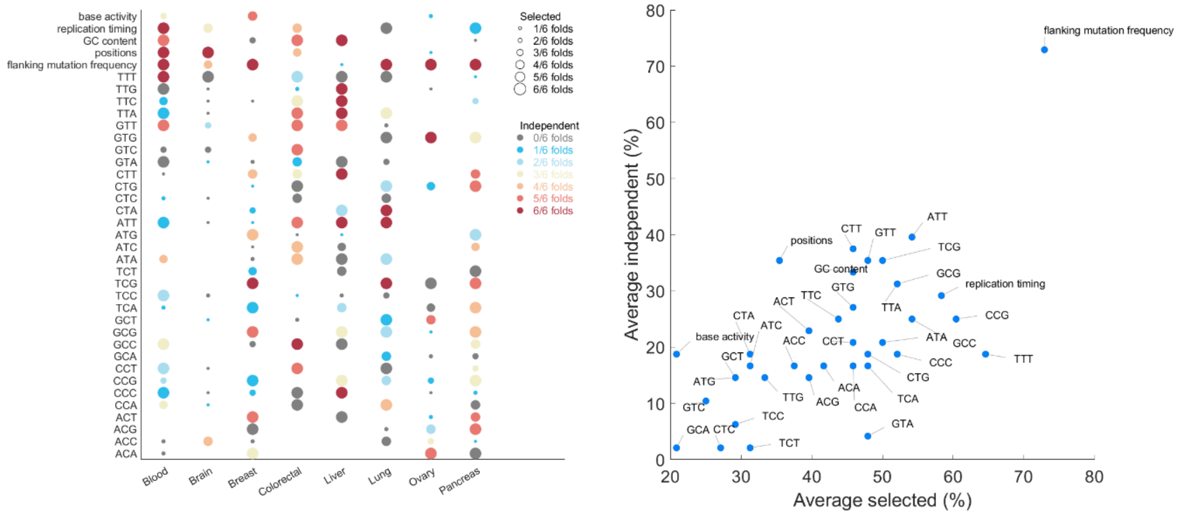

Supplementary Fig. 9 | Description of the covariate space (gene-level, all-CADD). a, Dot-plot representing the selected and independent covariates in the background mutagenesis model (all SNVs included). The rows represent covariates (features, predictors), while the columns represent tissues. The size of the dots represents the number of folds in which the features got selected into the multivariable model (based on performance in the multivariable model). The colour represents the number of folds, in which the feature had p-value < 0.05 in the multivariable model (i.e., acts as an independent predictor). **b,** The same data (averaged across tissues), shown as a scatterplot. The most consistently important feature is the flanking mutation frequency.

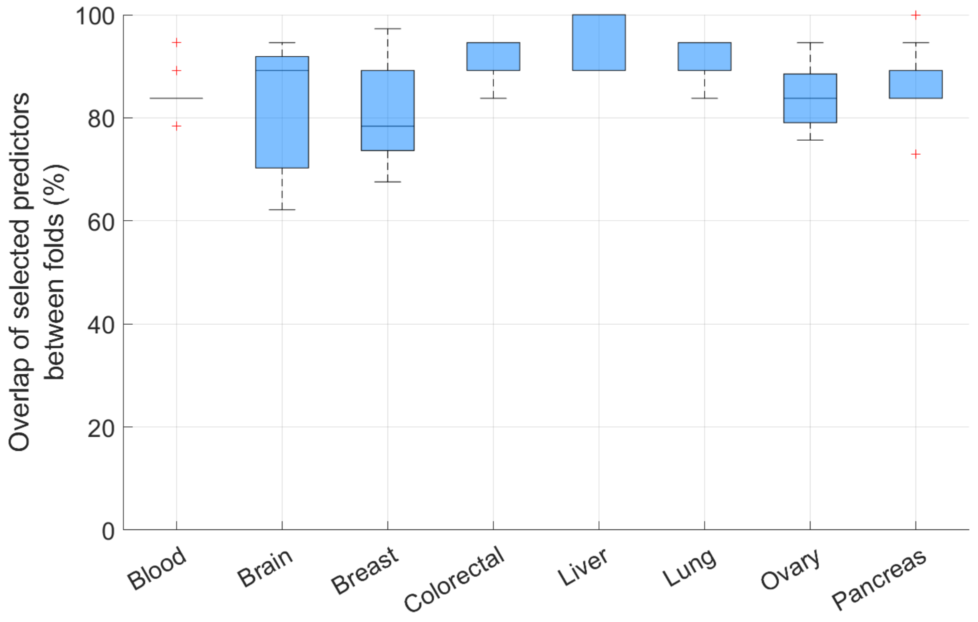

Supplementary Fig. 10 | Description of the covariate space (gene-level, all-CADD). Each datapoint in the boxplot represents a pair of folds, and the y-axis values shows the overlap of selected predictors in the two folds (selected into the multivariable model; computed as 100*mean(isUsed_fold1 == isUsed_fold2)). It shows that very similar predictors get selected across the folds.

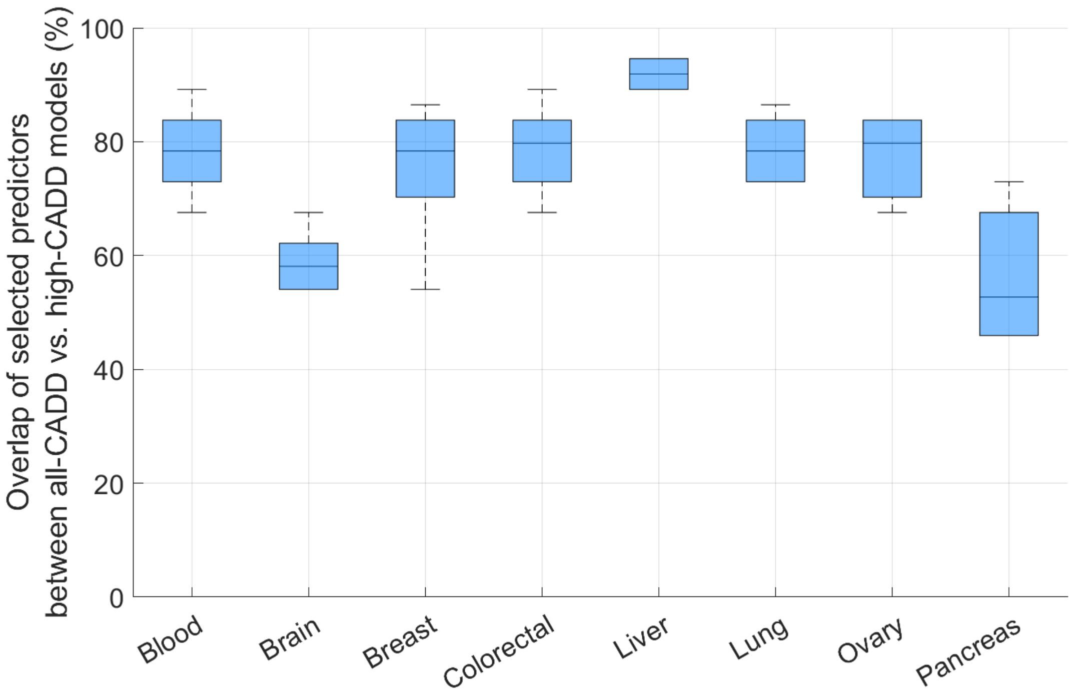

Supplementary Fig. 11 | Overlap of predictors selected in the all-CADD and high-CADD models. Each datapoint in the boxplot represents one of the 6 folds, and the y-axis values shows the overlap of selected predictors in the all-CADD vs. high-CADD model in the given fold (selected into the multivariable model; computed as 100*mean(isUsed_allCADD == isUsed_ highCADD)). It shows that to a large extend same predictors get selected in the all-CADD and high-CADD models.

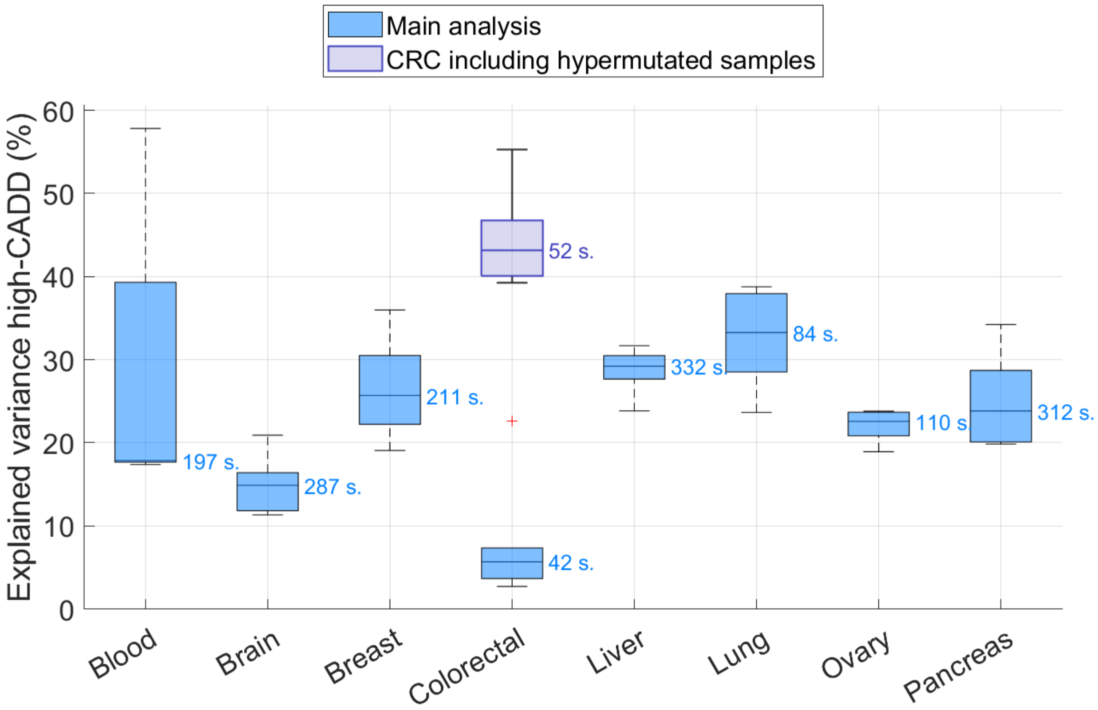

Supplementary Fig. 12 | Cross-validation evaluation of the background mutagenesis model (gene-level, high-CADD). As Supplementary Fig. 8, but only with high-CADD SNVs (CADD PHRED ≥ 10). The explained variance is slightly lower than in all-CADD case, as expected given the lower number of mutations. The boxplots show distribution of the explained variance across the 6 folds of the cross-validation. In every fold, the model was trained on 5/6th of the data and the remaining 1/6th was used for evaluation. The explained variance represents the square of the Pearson correlation coefficient of the predicted and observed mutation counts in the non-coding regulatory space of that gene. The number of used samples in each tissue are shown in blue on the right-hand side of each box. In colorectal cancer (CRC), a version with all 52 samples included (i.e., not excluding the hypermutated samples) is shown in violet.

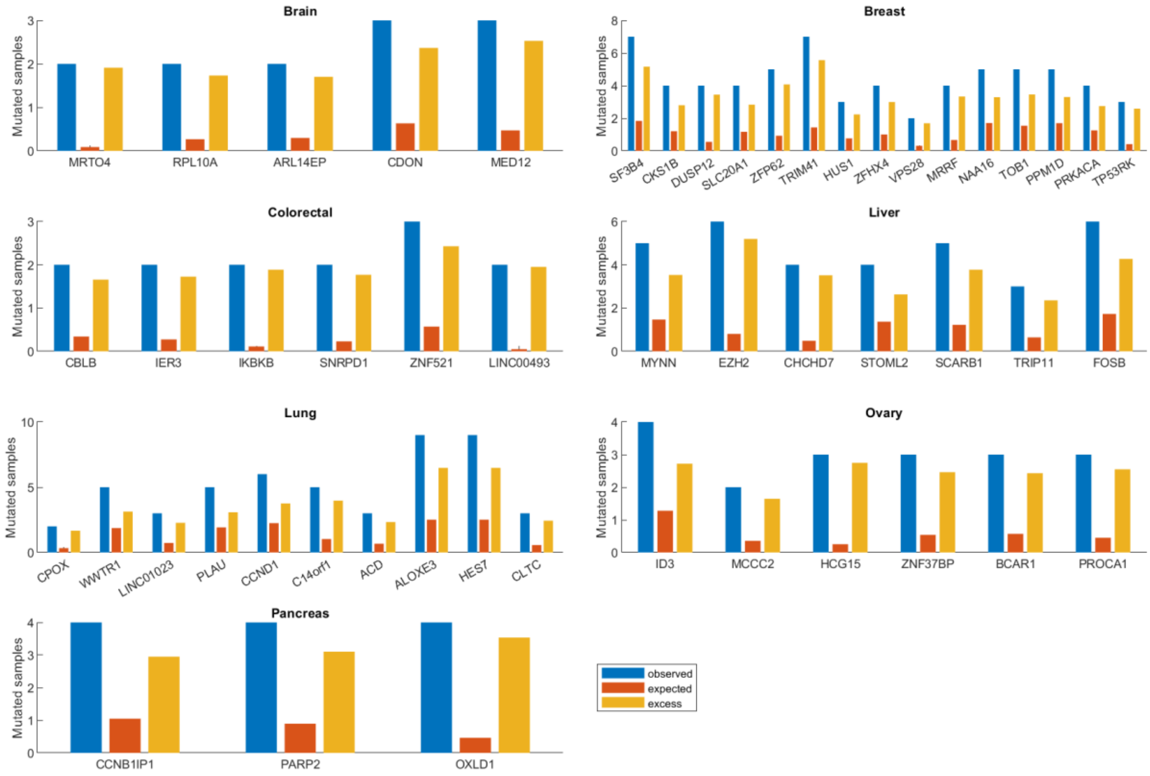

Supplementary Fig. 13 | Number of mutated samples candidate regulatory drivers in solid cancers, grouped by the target genes. The observed values are shown in blue, the expected values (based on the background mutagenesis model) are shown in orange, and the excess (observed – expected) values shown in yellow.

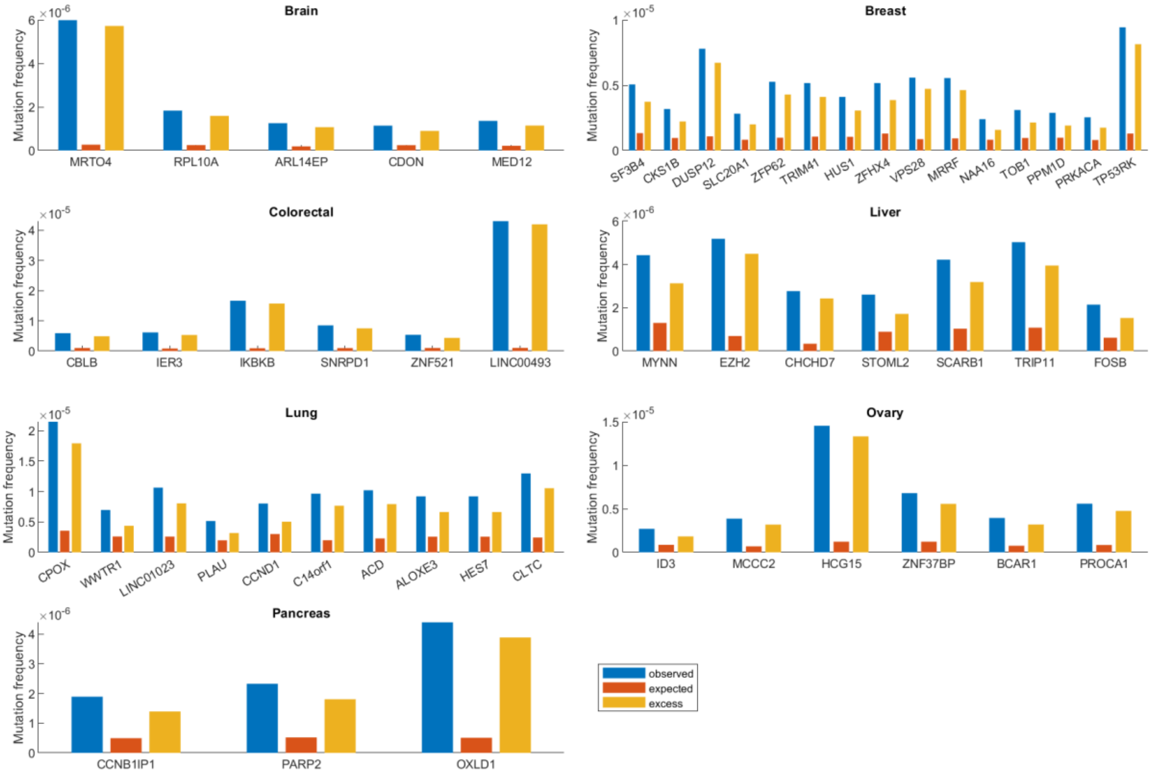

Supplementary Fig. 14 | Mutation frequency in candidate regulatory drivers in solid cancers, grouped by the target genes. The observed values are shown in blue, the expected values (based on the background mutagenesis model) are shown in orange, and the excess (observed – expected) values shown in yellow.

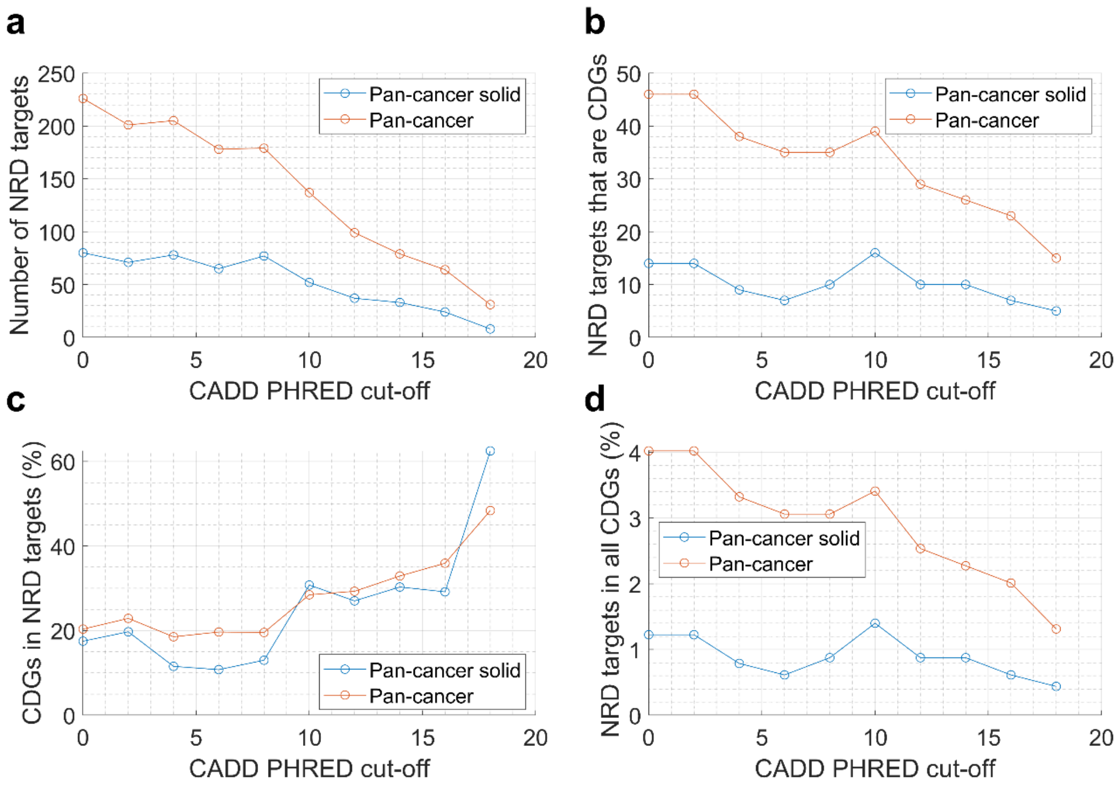

Supplementary Fig. 15 | Cancer driver genes (CDGs) in non-coding regulatory driver (NRD) targets with respect to CADD PHRED cut-off. a, The y-axis shows number of predicted NRD target "hits". The plot shows that with growing CADD PHRED cut-off, the number of discovery hits decreases, and therefore the statistical power of the Fisher test (and any downstream analyses) decreases. **b,** The y-axis shows the number of predicted NRD targets that are CDGs. **c,** The y-axis shows percentage of CDG within the predicted NRD target "hits", i.e., (CDG&hit)/hits. **d,** The y-axis shows percentage of predicted NRD target "hits" within all CDGs, i.e., (CDG&hit)/CDGs.

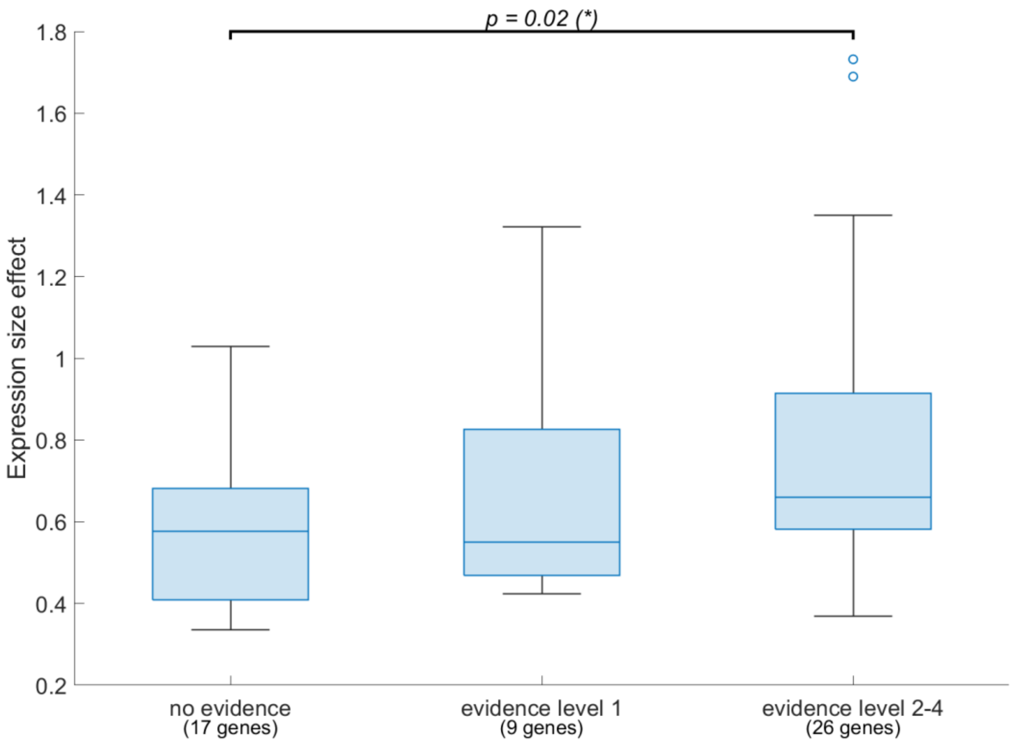

Supplementary Fig. 16 | Cancer evidence in the literature is associated with larger absolute expression size effect. The y-axis shows expression size effect in three groups of solid cancer hits, grouped by the level of tissue-matched cancer evidence in the literature (see methods for definition of the levels). Rank-sum test was used to compare the values between the first and third group.

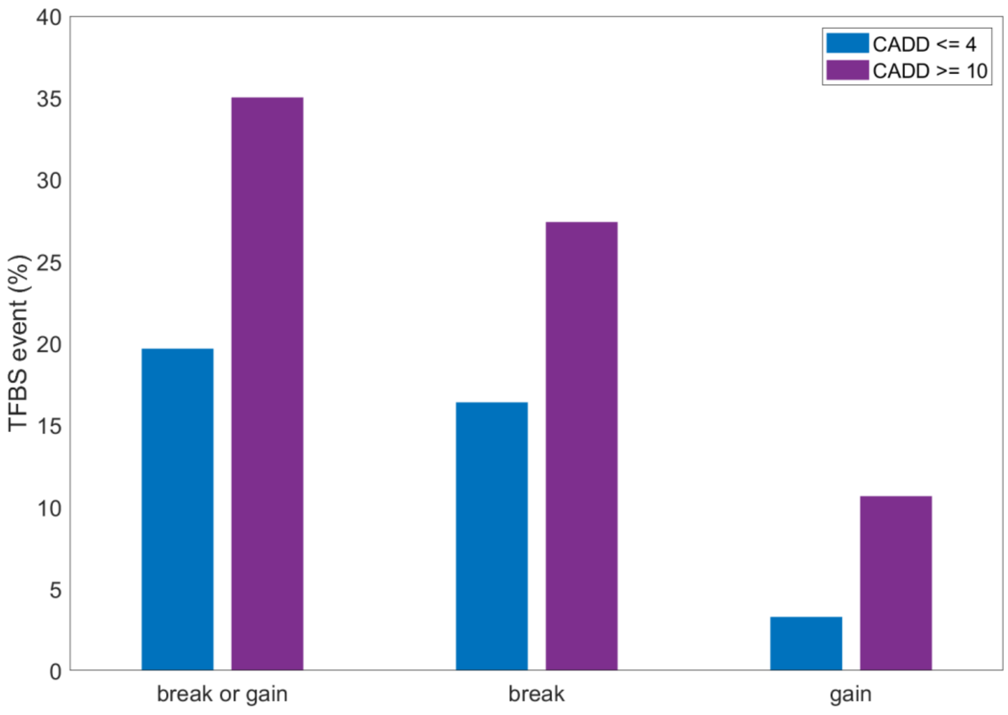

Supplementary Fig. 17 | Transcription factor binding site (TFBS) alterations are more frequent in the high-CADD non-coding regulatory driver mutations compared to the low-CADD ones (pan-cancer solid).

Comparison of low-impact (CADD PHRED score ≤ 4; blue) vs. high-impact (CADD PHRED score ≥ 10; purple) regulatory driver mutations in the terms of their percentage predicted to create a transcription factor binding site (TFBS) break (disruption) or gain (*de novo* TFBS) by the FunSeq2 tool.

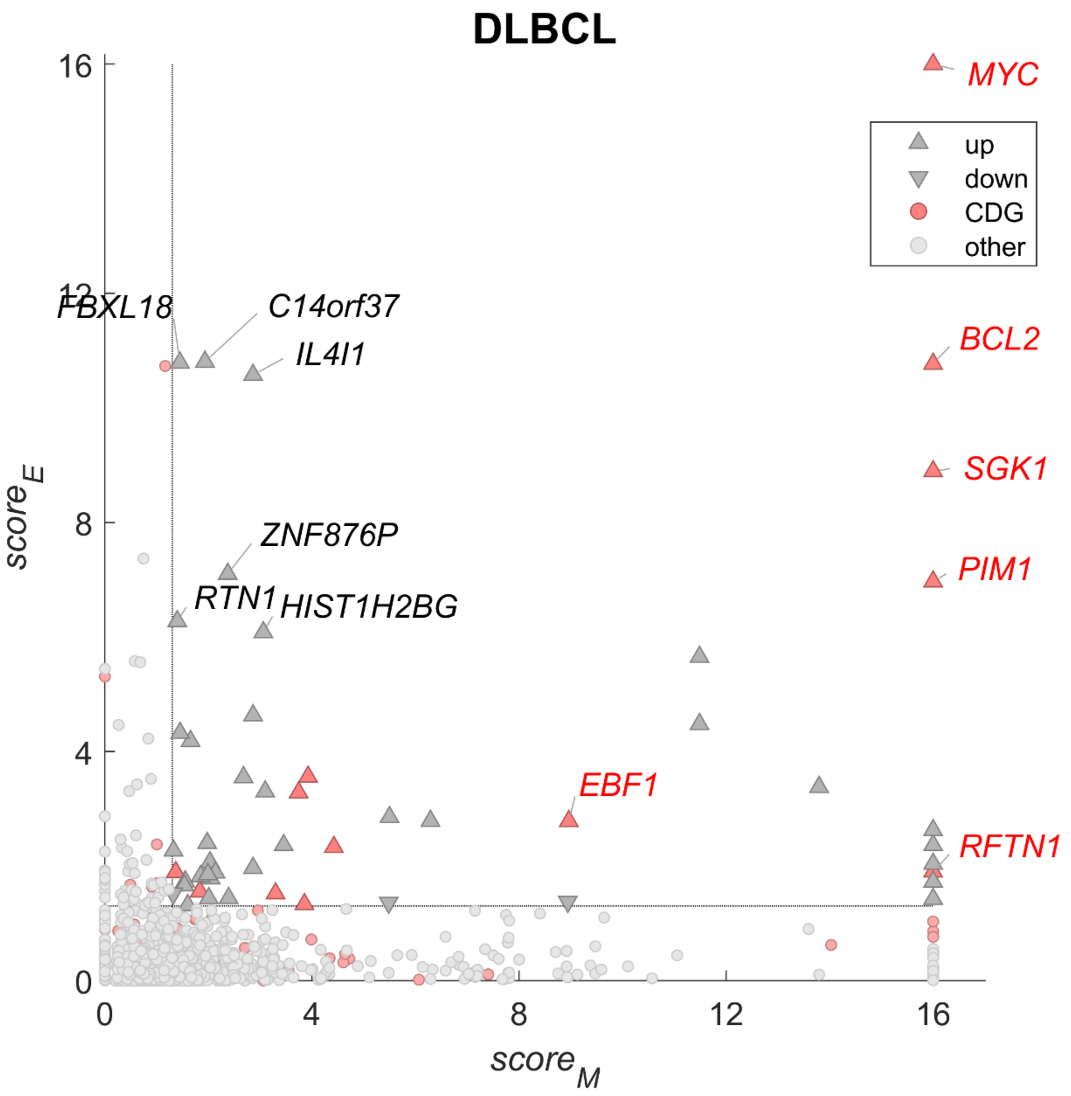

Supplementary Fig. 18 |48 driver-upregulated and 3 driver-downregulated genes (targets of non-coding regulatory driver candidates) in Diffuse Large B-Cell Lymphoma (DLBCL). Same as in Fig. 3a, but only in DLBCL subtype of blood cancers.

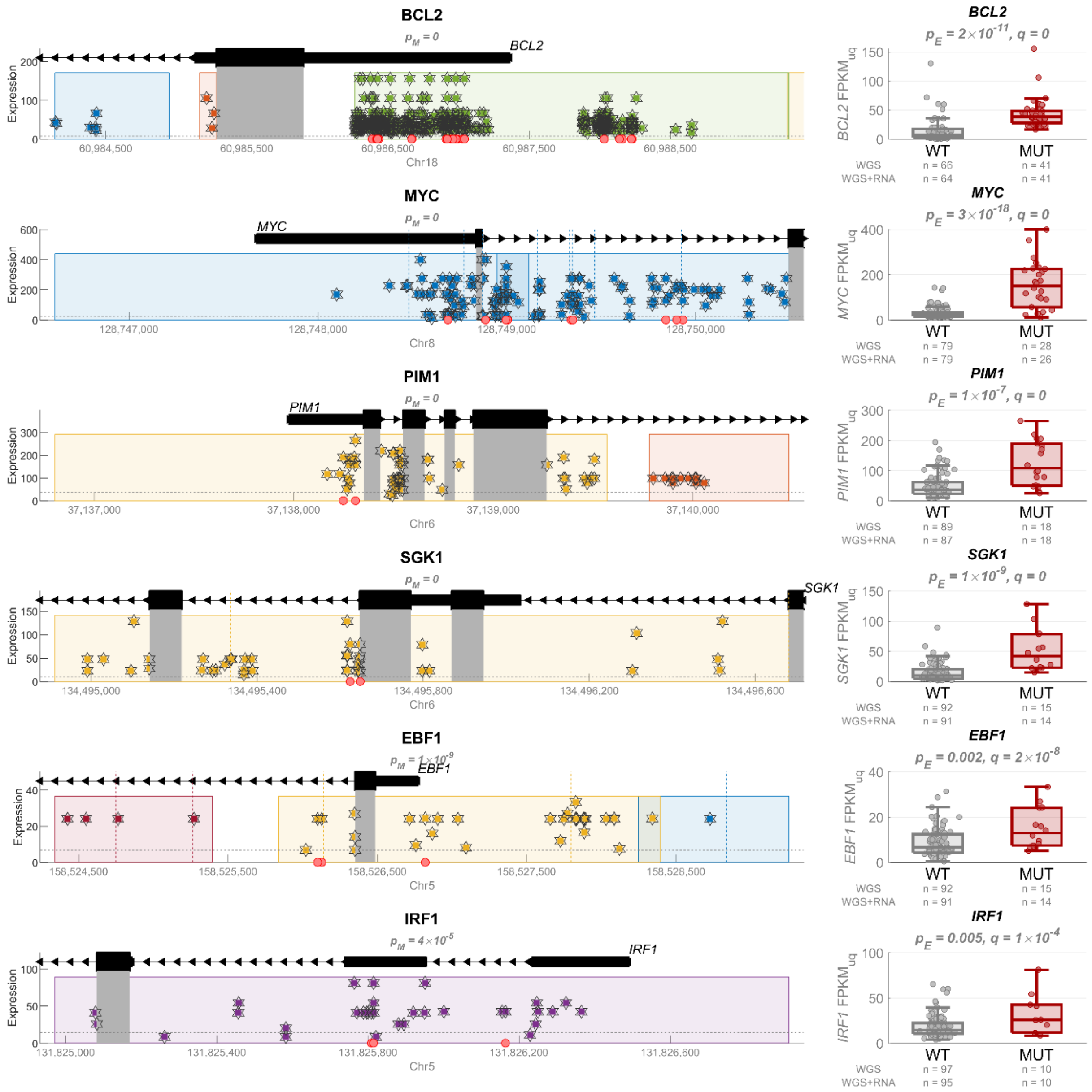

Supplementary Fig. 19 | Examples of 6 driver-upregulated genes in DLBCL. Same as in Fig. 3d-e, but only in DLBCL subtype of blood cancers. The red circles represent positions shown in Supplementary Fig. 21–23, predicted to disrupt TFBS of transcriptional repressors or create TFBS of transcriptional activators.

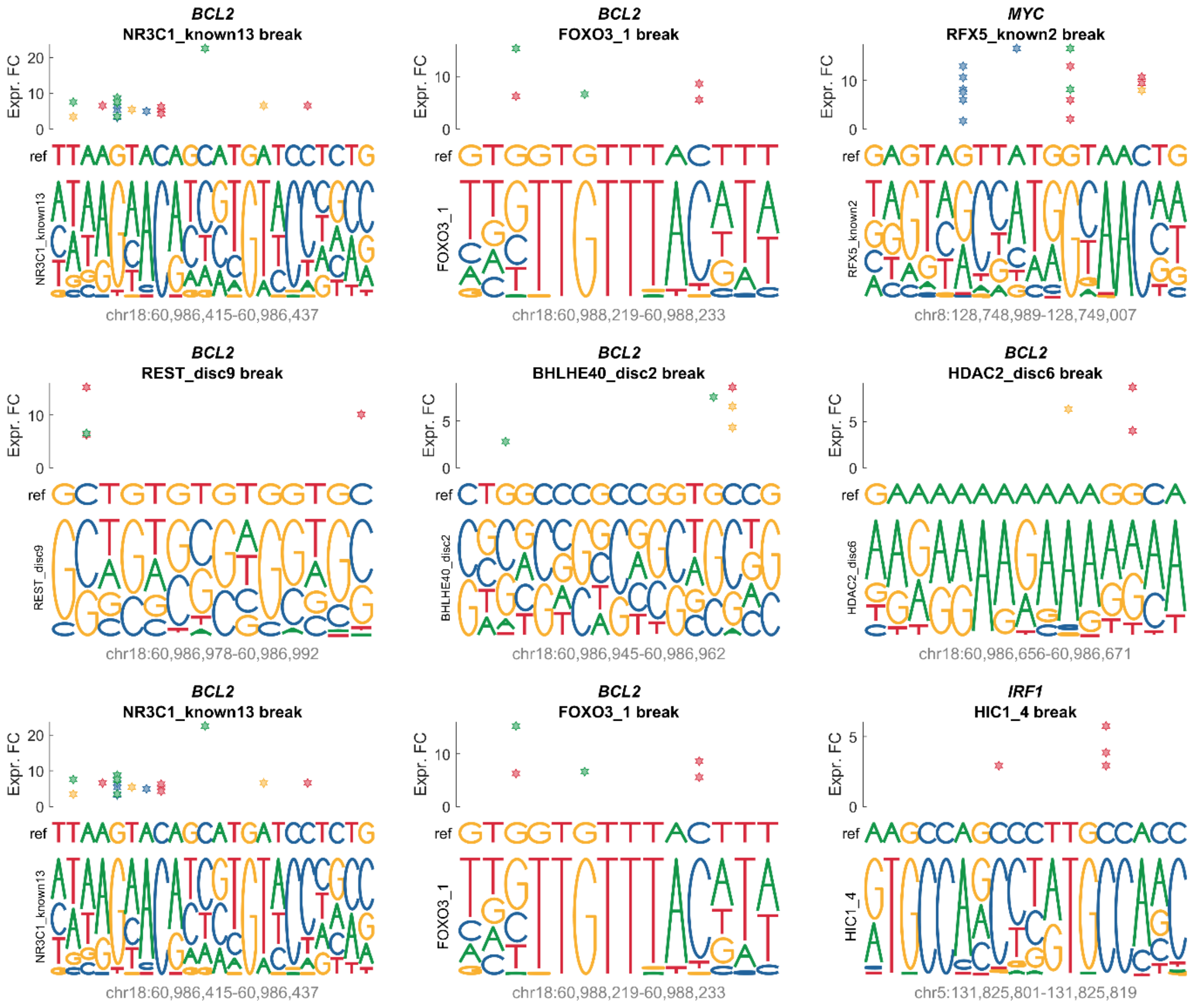

Supplementary Fig. 20 | Mutations in the binding sites of the top 9 mutated positions with potential to act as non-coding regulatory drivers in DLBCL via disruption of transcriptional repressor TFBS. For each of the 9 mutations shown in Fig. 8, this figure shows all the other mutations in that region that are high-CADD and predicted to disrupt the binding. The mutations are shown as stars, with colour coding the alt base change (A = green, C = blue, G = yellow, T = red). The y-axis represents the FPKM-UQ expression fold-change of the mutated sample vs. median of the wild-type samples. Note that two regions in the bottom row overlap with the regions in the first row, but were plotted like this, so that the order matches the 9 top hits shown in Fig. 8.

a

	Position	Base change	Samples	Target gene	Direction	Expression fold-change	CADD PHRED	Negative regulator TFBS break	Positive regulator TFBS gain
1	chr18:60,986,423	C>T	3	*BCL2*	upregulation	5.3 ± 1.0	18.5	NR3C1	NKX3-1
2	chr6:27,861,622	G>A	2	*HIST1H3J*	upregulation	13.0 ± 18.1	15.6		RXRA
3	chr18:60,986,386	T>C	2	*BCL2*	upregulation	10.6 ± 6.7	17.2		ETV4,SPI1
4	chr6:134,495,648	C>T	2	*SGK1*	upregulation	7.1 ± 1.9	35.0		SOX17
5	chr18:60,986,917	G>A	2	*BCL2*	upregulation	5.5 ± 4.4	16.5	EGR1	SP100
6	chr18:60,986,987	T>A	2	*BCL2*	upregulation	5.4 ± 1.6	18.0	GLI2	TCF12
7	chr18:60,988,160	C>T	2	*BCL2*	upregulation	4.9 ± 2.9	11.8		REST
8	chr18:60,988,139	A>C	2	*BCL2*	upregulation	4.6 ± 0.7	10.2	BHLHE40,REST	OVOL2
9	chr18:60,988,031	C>G	2	*BCL2*	upregulation	4.2 ± 1.1	12.4		SOX10

b

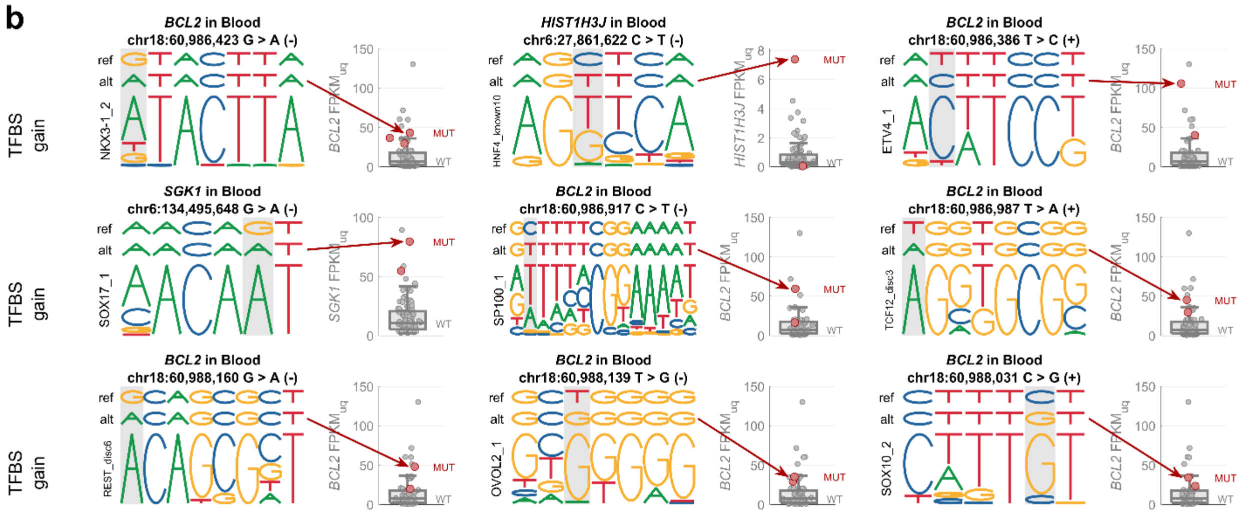

Supplementary Fig. 21 | List of top 9 mutated positions with potential to act as non-coding regulatory drivers in DLBCL via creation of transcriptional activator TFBS. a, The list of the 9 mutated positions (hg19), the base change (alternative allele), number of DLBCL samples with this mutation, the target gene they are predicted to regulate, the direction of the effect (all upregulated in mutated samples here), average and standard deviation of the expression fold-change between the mutated samples and median expression of the wild-type samples (FPKM-UQ), average CADD PHRED value, negative regulator TFs predicted to have binding disrupted by the mutation, and positive regulator TFs predicted to have binding created by the mutation. **b,** Genomic visualisation of the 9 top hits. In each example, the top row represents the reference sequence, the second row represents the mutated sequence (with the alt-allele base), and the bottom row represents the motif that is created by the mutation (the motifs are from ENCODE-motifs). The mutated position is highlighted by grey background. The boxplot on the right-hand side of each example shows the distribution of the target gene expression in the wild-type samples, while the red circles represent the expression level in the samples with the depicted mutation.

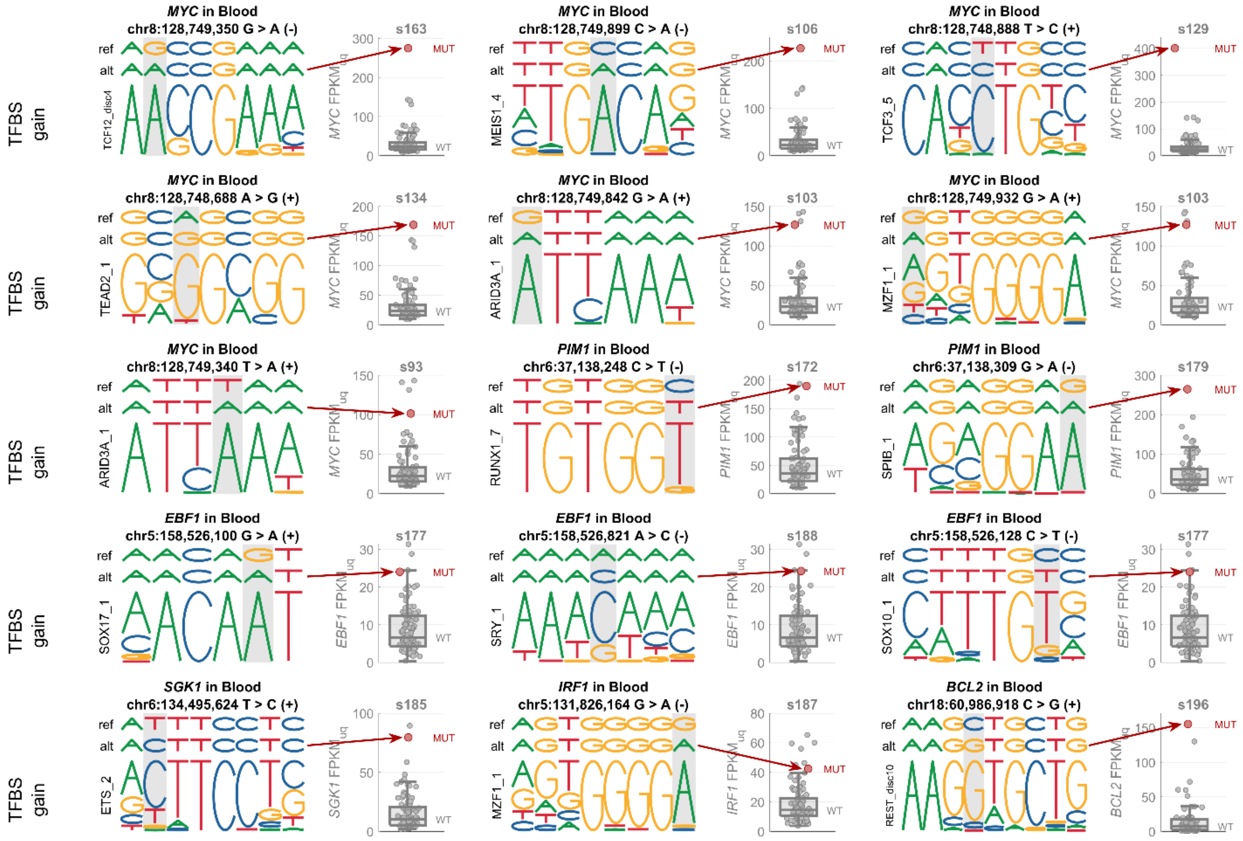

Supplementary Fig. 22 | Examples of non-recurrent mutations in DLBCL predicted to create transcription activator TFBS. Genomic visualisation of the 9 top hits. In each example, the top row represents the reference sequence, the second row represents the mutated sequence (with the alt-allele base), and the bottom row represents the motif that is created by the mutation (the motifs are from ENCODE-motifs). The mutated position is highlighted by grey background. The boxplot on the right-hand side of each example shows the distribution of the target gene expression in the wild-type samples, while the red circles represent the expression level in the samples with the depicted mutation.

Supplementary Table Legends

Supplementary Table 1. | Used tissues. For each of the used tissues: the ABC map name, The Human Protein Atlas tissue name, the DepMap tissue name, the number of included WGS samples, WGS samples with RNA-seq, the list of PCAWG projects, unique enhancers, enhancer-gene pairs, the number of expressed genes with ABC enhancers, the number of driver-upregulated genes, the number of driver-downregulated genes, the number of observed CDG genes within the targets of regulatory driver candidates, the number of expected CDG genes within the targets of regulatory driver candidates, the CDG enrichment, the CDG enrichment p-value (two-tailed Fisher test).

Supplementary Table 2. | Target genes of non-coding regulatory driver candidates in solid cancers. For each gene in the list: chromosome, start position, end position, gene symbol, ENCODE/ENSEMBL gene id, strand, tissue of the regulatory driver candidate, is driver-upregulated, pM, pE, combined q-value, size effect of pM, size effect of pE, number of mutated samples (in the non-coding regulatory space), number of high-CADD-mutated samples (in the non-coding regulatory space), number of mutated samples with RNA-seq (in the non-coding regulatory space) , number of all candidate driver non-coding regulatory mutations, number of high-CADD candidate driver non-coding regulatory mutations, percentage of candidate driver non-coding regulatory mutations that are in the promoter of this gene (up to 250 bp from the TSS), percentage of candidate driver non-coding regulatory mutations in distance over 20 kb from this TSS, percentage of candidate driver non-coding regulatory mutations closer to a TSS of different gene compared to this gene, is CDG (in CGC or PCAWG), is CGC oncogene, is CGC tumour-suppressor gene (TSG), prognostic (F = favourable; U = unfavourable; ***p<0.001; **p< 0.01; *p< 0.05), prognostic p-value, level of tissue-matched literature evidence as oncogene (1 = very weak evidence; 2 = weak evidence; 3 = strong evidence; 4 = very strong evidence), level of tissue-matched literature evidence as TSG, average tissue-matched DepMap dependency score, average tissue-matched DepMap dependency score, percentage tissue-matched dependent cell-lines in DepMap, percentage of mutated samples creating a TFBS break, percentage of mutated samples creating a TFBS gain.

Supplementary Table 3. | Literature evidence for tissue-matched oncogenes and TSG. Level of evidence as oncogene or TSG in the unbiased literature search for the 48 driver-upregulated and 4 driver-downregulated genes in solid cancers. Level of evidence: 1 = very weak/indirect tissue-specific evidence, often supported by stronger experimental evidence from several other tissues. 2 = weak tissue-specific evidence, based on data on expression, survival, and generally dry-lab studies. 3 = substantial tissue-specific evidence, supported by wet-lab experiments *in vitro* and/or *in vivo*. 4 = very strong tissue-specific evidence, active research/use of the gene as a drug target.

Supplementary Table 4. | Candidate driver regulatory high-CADD mutations and their differentially expressed target genes. The list of the 216 mutation-gene pairs in solid cancers as in Fig. 7.

Supplementary Table 5. | Used datasets. For each used dataset, we provide the name, link, and additional information.

Supplementary Note 1: Code Dependencies

MATLAB (2022a) and bash have been used in this study.

The following MATLAB toolboxes have been used:

- Statistics and Machine Learning Toolbox (version 12.3)
- Bioinformatics Toolbox (version 4.16)

The following MATLAB libraries have been used:

- ViolinGit: Bechtold and Bastian (2016). Violin Plots for Matlab, Github Project https://github.com/bastibe/Violinplot-Matlab, DOI: 10.5281/zenodo.4559847
- lbmap: Robert Bemis (2022). Light Bartlein Color Maps (https://www.mathworks.com/matlabcentral/fileexchange/17555-light-bartlein-color-maps), MATLAB Central File Exchange. Retrieved June 23, 2022.
- myBinomTest: Matthew Nelson (2022). myBinomTest(s,n,p,Sided) (https://www.mathworks.com/matlabcentral/fileexchange/24813-mybinomtest-s-n-p-sided), MATLAB Central File Exchange. Retrieved June 23, 2022.
- linspecer: Jonathan C. Lansey (2022). Beautiful and distinguishable line colors + colormap (https://www.mathworks.com/matlabcentral/fileexchange/42673-beautiful-and-distinguishable-line-colors-colormap), MATLAB Central File Exchange. Retrieved June 23, 2022.
- EmpiricalBrownsMethod: William Poole, Theo Knijnenburg, David L Gibbs (2016). CombiningDependentPvaluesUsingEBM (https://github.com/IlyaLab/CombiningDependentPvaluesUsingEBM/blob/master/Matlab/EmpiricalBrownsMethod.m), Retrieved June 23, 2022.

The other software used:

- bedtools (version 2.27.0)

The code developed in this study is available at https://github.com/tomkovam/Dr.NOD.
