# Supplementary material for: Dr.Nod: computational framework for discovery of regulatory non-coding drivers in tissue-matched distal regulatory elements
Source: Nucleic Acids Res. 2023 Jan 10;51(4):e23. doi: 10.1093/nar/gkac1251 (PMC9976879; doi:10.1093/nar/gkac1251)
Supplement: gkac1251_Supplemental_Files [file gkac1251_supplemental_files.zip › SupplementaryTable3.pdf]

Supplementary Table 3. | Literature evidence for tissue-matched oncogenes and TSG. Level of evidence as oncogene or TSG in the unbiased literature search for the 48 driver-upregulated and 4 driver-downregulated genes in solid cancers.

Direction: U = upregulated, D = downregulated (in samples with mutation in its regulatory regions)

CGC = Cancer Gene Census, O = oncogene, T = tumour-suppressor gene, F = fusion

Level of evidence as oncogene or TSG = tumour-suppressor gene:

1. very weak/indirect tissue-specific evidence, often supported by stronger experimental evidence from several other tissues
2. weak tissue-specific evidence, based on data on expression, survival, and generally dry-lab studies
3. substantial tissue-specific evidence, supported by wet-lab experiments *in vitro* and/or *in vivo*
4. very strong tissue-specific evidence, active research/use of the gene as a drug target

Tissue	Direction	Symbol	Aliases	Evidence Summary	Oncogene	TSG	CGC	Survival
Brain	U	ARL14EP	ADP Ribosylation Factor Like GTPase 14 Effector Protein| ARF7EP | C11orf46	ARL14EP (C11orf46) is located in a neurodevelopmental and WAGR syndrome (Wilms Tumour, Aniridia, Genitourinary Abnormalities, intellectual disability) risk locus, it is important in neuronal development, and it is a binding partner of the SETDB1 protein complex [1], an important oncogene in many cancers, including brain cancers.[2,3]	1			
Brain	U	CDON	Cell Adhesion Associated, Oncogene Regulated | CDO	CDON was shown to have tumour-suppressive and oncogenic roles in various cancers[4–8], both linked to the Hedgehog (HH) signalling, an important pathway in brain cancers[9,10]. We have not found any direct literature on the role of CDON in brain cancer.				
Brain	D	MED12	Mediator Complex Subunit 12	MED12 is annotated as tumour-suppressor gene in CGC[2], it is implicated in brain development and neuropsychiatric disorders[11], and it is frequently mutated in medulloblastoma[12]. In a variety of cancer cell types, the loss of MED12 elicits chemoresistance by activating the TGF-β pathway[13–15], a pathway that in brain cancers promotes oncogenic development and progression to the more malignant state[16]. High MED12 expression is weakly predictive of favourable prognosis in brain cancer in the Protein Atlas ($p = 0.006$).[17]		2	T	F**
Brain	U	MRTO4	MRT4 Homolog, Ribosome Maturation Factor | MRT4	We have not found any literature on the role of MRTO4 in brain cancer.				

Brain	U	RPL10A	Ribosomal Protein L10a	Oncogenic roles of RPL10A have been suggested in a variety of cancer types[18]. RPL10A was one of the top eight genes important in early brain development and predictive of poor brain cancer progression.[19]	2			F*
Breast	U	CKS1B	CDC28 Protein Kinase Regulatory Subunit 1B | CKS1	CKS1B is frequently overexpressed in breast cancers, its overexpression is associated with lymph node metastasis and poor prognosis, its knockdown by RNA inference inhibits growth, promotes apoptosis, and decreases cell migration and invasion ability, while overexpression inhibits apoptosis of breast cancer cells.[20] CKS1B acts as oncogene and drug-resistance gene also in many other cancer types.[21]	3			U*
Breast	U	DUSP12	Dual Specificity Phosphatase 12 | YVH1	DUSP12 was suggested to act as a candidate oncogene in other cancer types[22,23], other DUSP proteins have been implicated in breast cancer[24–26], and DUSP12 was identified as one of the most overexpressed genes in metastatic breast cancers.[27]	1			U*
Breast	U	HUS1	HUS1 Checkpoint Clamp Component | HHUS1	Together with checkpoint proteins RAD9 and RAD1, HUS1 is a component of genotoxin-activated checkpoint complex that is involved in the cell cycle arrest in response to DNA damage.[28] Hus1 is upregulated in tumour-initiating cells from p53-null mouse mammary tumours[29], Hus1 loss sensitizes p53-deficient cells to apoptosis *in vivo*[30], Hus1 is required for the survival and proliferation of mammary epithelium[30], and HUS1's partner, RAD9, is also oncogenic in breast cancer.[31] $\mathit{Hus1}^{neo/\Delta 1}$ mice show hypersensitivity to agents that cause replication stress.[32]	3			
Breast	U	MRRF	Mitochondrial Ribosome Recycling Factor | RRF | MTRRF	MRRF is a component of the mitochondrial translational machinery.[28] We have not found any literature on the role of MRRF in breast cancer.				
Breast	U	NAA16	N-Alpha-Acetyltransferase 16, NatA Auxiliary Subunit | NARG1L	We have not found any literature on the role of NAA16 in breast cancer.				U*
Breast	U	PPM1D	Protein Phosphatase, Mg2+/Mn2+ Dependent 1D | PP2C-DELTA | WIP1	Ample evidence exists on the oncogenic roles of PPM1D in breast cancer.[33] For example, PPM1D silencing by RNA interference inhibits proliferation and induces apoptosis in breast cancer cells[34], overexpression of PPM1D promotes malignant progression of breast cancer by inactivating wild type p38 MAPK, p53, and p16,[35] gain-of-function mosaic mutations in PPM1D predispose to breast and ovarian cancers[36], inactivation of PPM1D reduces the proliferation rate of breast cancer cell lines and enhances growth inhibition caused by doxorubicin, and *in vivo*, administration of PPM1D inhibitors decreases proliferation of xenograph tumours and tumours developed in transgenic mice[37]. Therapeutic potential of PPM1D inhibition is being actively researched in pre-clinical studies, showing promising results so far.[33,37,38]	4		O	

Breast	U	PRKACA	Protein Kinase CAMP-Activated Catalytic Subunit Alpha | PKA C-Alpha	PRKACA mediates resistance to HER2-targeted therapy in breast cancer cells and restores anti-apoptotic signalling[39], PRKACA contributes to oestrogen-induced proliferation and endocrine resistance of breast cancer cells[40], as well as being an oncogene also in other tissues[2].	3		O	
Breast	D	SF3B4	Splicing Factor 3b Subunit 4 | SAP49	SF3B4 has been proposed to act as tumour-suppressor gene due to the presence of inactivating mutations in breast cancers.[41] In other tissues, SF3B4 seems to have mixed roles.[42,43]		1		
Breast	U	SLC20A1	Solute Carrier Family 20 Member 1 | Sodium-Dependent Phosphate Transporter 1 | PiT-1	SLC20A1 knockdown suppresses the viability and tumour-sphere formation of breast cancer cells and high levels of SLC20A1 are predictive of poor outcome in several subtypes of breast cancer patients.[44,45] SLC20A1 has oncogenic properties and promotes cell proliferation also in other cancer/cell types.[46–48]	3			
Breast	U	TOB1	Transducer Of ERBB2, 1 | TOB | TROB1	Both TSG and oncogenic roles of TOB1 have been documented in breast cancer. On one hand, TOB1 can act as TSG in breast cancer cells[49] and its overexpression can sensitise the cells to radiatiotherapy[50,51]. On the other hand, TOB1 is linked to poor prognosis in node-negative breast cancer[52], TOB1 amplified and/or overexpressed in invasive breast cancers, depletion of TOB1 selectively sensitizes oestrogen-independent cells to clinically important inhibitors of AKT and mTOR, and TOB1 inhibition induces G1/S arrest, supporting its proliferative survival roles in oestrogen-independent cells[53]. It has been shown that the switch from growth inhibitory to proliferative and pro-survival action is mediated by the phosphorylation of TOB1 and occurs also in other cancer types.[52–55]	3	3		
Breast	U	TP53RK	TP53 Regulating Kinase | P53-Related Protein Kinase | C20orf64 | TPRKB | PRPK	TP53RK is a kinase that interacts with, phosphorylates, and activates TP53.[56] TP53RK has been identified as a common essential gene in cancer cell lines by DepMap.[57] TP53RK were observed to be overexpressed in both lobular and ductal breast cancer versus normal tissue.[58] TP53RK was identified as the strongest hit in a kinase-enriched small interfering RNA chemosensitization screen of genes restraining apoptosis after mitotic stress in a variety of cell lines.[58] Oncogenic properties of TP53RK have been shown also in other cancer types.[59–61] Finally, TP53RK stabilises a TP53RK-binding protein (TPRKB), which was identified as the top hit in a shRNA screen of vulnerable genes in *TP53*-mutated cancers.[56] Knockout of both TP53RK and even more so of TPRKB reduced proliferation in *TP53*-mutated but not TP53-wild type cell lines (including breast cancer cell line).[56]	3			
Breast	U	TRIM41	Tripartite Motif Containing 41 | E3 Ubiquitin-Protein Ligase TRIM41 | RING-Finger Protein That	TRIM41 (RINCK) is an E3 ligase of Protein kinase C (PKC)[62] and enables inhibition of Doxorubicin-Induced Senescence in breast cancer cells[63].	1			

			Interacts With C Kinase | RINCK					
Breast	U	VPS28	VPS28 Subunit Of ESCRT-I	Knockdown of VPS28 leads to suppression of cell migration, proliferation, and invasion and enhancement of apoptosis in breast cancer cell lines[64]. VPS28 expression is elevated in breast cancer tissue, especially in the luminal B subtype, and high expression of VPS28 is predictive of poor survival.[64,65]	3			U*
Breast	U	ZFHX4	Zinc Finger Homeobox 4 | ZFH4	Oncogenic properties of ZFHX4 have been observed in several cancer types.[66–69] In breast cancer, ZFHX4 was proposed to mediate chemoresistance[70], and showed decreased methylation in non-responders to neoadjuvant chemotherapy in triple-negative breast cancer.[71] We have not found any literature with direct experimental evidence on the role of ZFHX4 in breast cancer.	1			
Breast	U	ZFP62	ZFP62 Zinc Finger Protein | ZNF755	We have not found any literature on the role of ZFP62 in breast cancer.				U*
Colorectal	D	CBLB	Cbl Proto-Oncogene B Cas-Br-M (Murine) Ecotropic Retroviral Transforming Sequence B | E3 Ubiquitin-Protein Ligase CBL-B | Cbl-B | RNF56	CBLB (Cbl-b) is annotated as a CGC tumour-suppressor gene.[2] CBLB, as well as its paralog CBL, cause degradation of an important oncogene EGFR (epidermal growth factor receptor).[72,73] In lung and colorectal cancer, CBLB and CBL were shown to downregulate PD-L1, an important immune suppressive protein.[74,75] A role of CBLB in tumour-suppression and/or sensitivity has been demonstrated in breast[76–79], lung[80] and gastric[77,79,81] cancers, while oncogenic properties have been described in T-cells, making it a promising target for CAR T-cell therapy (also for colorectal tumours)[82,83]. While we have not found literature on experimental evidence of TSG role of CBLB specifically in colorectal cancer, it has been well documented for its paralog CBL[75,84,85].		1	T	F*
Colorectal	U	IER3	Immediate Early Response 3 | PRG1 | IEX-1L | IEX-1 | IEX1	IER3 functions in the protection of cells from Fas- or tumour necrosis factor type alpha-induced apoptosis.[28] IER knockout mice treated with carcinogens demonstrated substantially reduced tumour formation, colitis, and inflammatory responses.[86] IER3 showed increased expression in colorectal cancer compared to normal tissue in cancer patients and mouse colon adenoma tumours. [87–89] but not in 10 early-stage colorectal cancer patients[90]. IER3 expression was also detected in circulating tumour cells (CTCs) of rectal cancer patients, and the number of IER3-positive CTCs correlated with tumour size.[91]	3			
Colorectal	U	IKBKB	Inhibitor Of Nuclear Factor Kappa B Kinase Subunit Beta | IKK-Beta | IKKβ | NFKBIKB | IKK2 | IKKB	IKBKB (IKKβ) plays an important role in activation of the oncogenic NF-kappa-B pathway.[28] It is annotated as CGC oncogene[2], and plays a role in many biological processes including proliferation, cell survival, migration, metastasis, DNA damage response, metabolism, inflammation, and immunity[2,92]. Depending on the context, IKBKB can play both pro-tumorigenic and anti-tumorigenic roles even in the same cancer type.[92] Apart from mixed observations in mesenchymal cells[92–94], numerous evidence from mouse models demonstrates the oncogenic role of IKBKB in	3	1	O	

				colorectal cancer. Cre-mediated *Ikbkb* deletion in intestinal epithelial cells resulted in lower colon cancer incidence by enhanced apoptosis during tumour promotion[95]. Cre-mediated *Ikbkb* deletion in myeloid cells resulted in lower colon cancer incidence and size mediated by a decrease in expression of proinflammatory mediators[95]. Expression of a constitutively active IKKβ in intestinal epithelial cells of transgenic mice induced spontaneous intestinal tumour formation, as well as enhanced tumorigenesis in models of carcinogen- or mutation-induced colorectal cancer[96,97], mediated by activation of Wnt signalling and production of pro-inflammatory intestinal microenvironment[96] or induction of oxidative/nitrosative DNA damage[97].				
Colorectal	U	LINC00493		LINC00493 is a widely expressed long noncoding RNA and contains a small open reading frames that is translated into a small protein SMIM26.[98] siRNA knockdown reduced cell viability in HEK293T and A375 cell lines, while the opposite effect was observed in MDA-MB-231.[98] We have not found any literature on the role of LINC00493 in colorectal cancer.				
Colorectal	U	SNRPD1	Small Nuclear Ribonucleoprotein D1 Polypeptide | Sm-D1 | HsT2456	SNRPD1 is a spliceosome-associated protein. SNRPD1 was identified as one of the top three differentially expressed genes encoding molecules involved in proliferation in intestinal cells.[99] SNRPD1 was also elevated in intestinal cells treated with a gram-negative bacterium *Akkermansia mucinipila*, that enhances proliferation *in vitro* and promotes colorectal cancer *in vivo*.[100] Oncogenic properties of SNRPD1 have been also demonstrated in other cancer types.[101–103]	2			
Colorectal	U	ZNF521	Zinc Finger Protein 521 | Early Hematopoietic Zinc Finger Protein | EHZF | Evi3 | LIP3	ZNF521 is annotated as a CGC oncogene[2], and its oncogenic role has been demonstrated in several cancer types[104–106]. We found only one study with direct experimental evidence on the role of ZNF521 in colorectal cancer; however, the study is not published in English.[107] In there, siRNA knockdown of ZNF521 inhibited the proliferation, migration, and invasion of colon cancer cells, and promoted apoptosis of colon cancer cells.[107]	2		O	
Liver	U	CHCHD7	Coiled-Coil-Helix-Coiled-Coil-Helix Domain Containing 7 | COX23	CHCHD7 is annotated as fusion CGC driver gene with a translocation partner PLAG1. We have not found any literature on the role of CHCHD7 in liver cancer.			F	
Liver	U	EZH2	Enhancer Of Zeste 2 Polycomb Repressive Complex 2 Subunit | KMT6 | ENX-1	EZH2 is an important epigenetic regulator, with numerous roles in cancer, including tumour initiation, metastasis, immunity, metabolism, drug resistance, and others.[108] Many drugs targeting/inhibiting EZH2 are being evaluated in pre-clinical studies and trials of all phases.[108] EZH2 promotes hepatocellular carcinoma (HCC) progression and metastasis by suppression of tumour-suppressor miRNA genes[109,110] and other mechanisms[111]. EZH2 is highly upregulated in liver cancer compared to normal tissue[112], its expression levels correlate with	4		O T	U***

				immunosuppression and increase with stage and grade[112] and are highly predictive of poor survival[17,112] ($p = 2\times10^{-6}$ in Protein Atlas). Inhibition of *EZH2* is an actively researched therapeutic strategy in liver and other cancers[108,113], helping to overcome the resistance to sorafenib[114,115], or in combination with immunotherapy[113,116].				
Liver	U	FOSB	FosB Proto-Oncogene, AP-1 Transcription Factor Subunit | G0S3 | GOSB | GOS3 | AP-1	FOSB is one of the components of the transcription factor complex AP-1, a regulator of cell proliferation, differentiation, and transformation. Activation of AP-1 is an early event in HCC and induction of AP-1 by YAP/TAZ contributes to cell proliferation and organ growth.[117,118] The role of FOSB itself in liver cancer is complex. FOSB levels are decreased in liver cancer compared to normal tissue and high FOSB expression is predictive of favourable prognosis.[119] FOSB exists in two isoforms, and the longer isoform promotes programmed cell death triggered by TGF-β, whereas the shorter isoform counteracts this and increases resistance to apoptosis of HCC cells[120], similarly as in breast cancer cells[121].	3	3		F**
Liver	U	MYNN	Myoneurin | Zinc Finger And BTB Domain-Containing Protein 31 | ZBTB31 | SBBIZ1 | ZNF902	We have not found any literature on the role of MYNN in liver cancer.				U*
Liver	U	SCARB1	Scavenger Receptor Class B Member 1 | CLA-1 | SR-BI | SRB1 | SR-B1	SCARB1 (SR-B1) is a high-density lipoprotein receptor that facilitates the uptake of cholesterol esters from circulating lipoproteins, it is consistently overexpressed by most tumours, it has an important role in cholesterol metabolism, signalling, motility, and proliferation of cancer cells, and it has been researched as a potential gateway for the delivery of therapeutic agents[122], also in liver cancer[123–125]. SCARB1 is a key host factor for an entry of Hepatitis C virus (HCV)[126,127], the infection of which can develop into hepatocellular carcinoma (HCC). Oncogenic properties of SCARB1 have been described in several cancer types.[122,128–131]. In liver cancer, SCARB1 is strongly prognostic of poor survival[17,132] ($p = 2\times10^{-5}$ in Protein Atlas), is increased in cancer versus normal tissue[132], and has oncogenic properties in its circular RNA form[133].	2			U***
Liver	U	STOML2	Stomatin Like 2 | Stomatin-Like Protein 2, Mitochondrial | SLP-2 | HSPC108	STOML2 potentiates metastasis of hepatocellular carcinoma by promoting PINK1-mediated mitophagy and decreases sensitivity to lenvatinib *in vitro* and *in vivo*.[134] Upregulation of STOML2 accelerated colony formation, migration, and invasion in HCC cells.[134] siRNA knockdown of STOML2 significantly repressed the viability, migration, and invasion of HCC cells via suppressing the NF-κB pathway.[135] STOML2 is upregulated in liver cancer compared to normal tissue[134,135], the upregulation is correlated with tumour size, histologic grade, metastasis, and higher probability of recurrence after hepatectomy[134,135], and high STOML2 expression is strongly	3			U***

				predictive of poor survival[17,134] (p = 0.0005 in Protein Atlas). Oncogenic properties of STOML2 have been demonstrated also in other cancers[136–140], including a pancreatic cancer, where STOML2 promoted liver metastasis *in vivo*[140].				
Liver	U	TRIP11	Thyroid Hormone Receptor Interactor 11 | GMAP-210 | CEV14 | Trip230	TRIP11 was identified based on its interaction with thyroid hormone receptor beta.[28] TRIP11 is a coactivator of transcription mediated by thyroid hormone receptor (THR) and hypoxia induced factor (HIF).[141] TRIP11 was suggested to contribute to renal cancer progression, where the expression of TRIP11 was observed to correlate with tumour grade and predict poor prognosis.[141] In HCC liver cancer, it was observed that thyroid hormone can promote cell invasion, proliferation, migration, angiogenesis, drug resistance, but in some cases also growth inhibition.[142,143] We have not found any direct experimental evidence in the literature on the role of TRIP11 in liver cancer, but it's high expression is weakly associated with poor prognosis in liver cancer in the Protein Atlas (p = 0.004).	1		F	U**
Lung	U	ACD	ACD Shelterin Complex Subunit And Telomerase Recruitment Factor | Adrenocortical Dysplasia Protein Homolog | TINT1 | PIP1 | TPP1 | PTOP	ACD gene encodes a protein called TPP1, one of the 6 members of the telomerase-maintenance complex shelterin.[28,144] TPP1 regulates recruitment of telomerase to telomeres and stimulating telomerase processivity.[144] Inhibition of the recruitment of telomerase by TPP1 leads to suppressed cell proliferation and tumour growth through shortening telomeres and inducing cell apoptosis in lung cancer cells *in vitro* and *in vivo*.[145] TPP1 overexpression in lung of mice increases telomere capping and leads to lengthened telomeres and expansion of AEC2 stem cell population.[146] Telomerase capping allows cancer cells to continue proliferating despite chromosome aberrations.[147] TPP1 is overexpressed in multiple cancers, including cervical cancer, where it is thought to positively regulate hTERT[148], and colorectal cancer, where it confers resistance to radiation therapy.[149]	3			U*
Lung	U	ALOXE3	Arachidonate Lipoxygenase 3 | Hydroperoxy Icosatetraenoate Dehydratase | ELOX3 | E-LOX	ALOXE3 predicts poor prognosis in lung cancer in the Protein Atlas (p = 0.0004)[17] and DNA methylation in ALOXE3 promoter is inversely associated with lung and prostate cancer incidence.[150] We have not found any direct evidence on the role of ALOXE3 in lung cancer.	1			U**
Lung	U	C14orf1	Ergosterol Biosynthesis 28 Homolog | ERG28 | NET51 | Chromosome 14 Open Reading Frame 1 | C14orf1	We have not found any literature on the role of C14orf1 (ERG28) in lung cancer.				
Lung	U	CCND1	Cyclin D1	CCND1 is an established oncogene, dysregulated in many cancers, commonly overexpressed by copy number alteration.[151] It activates cyclin-dependent kinases	4		O	U*

				(CDKs), and both CCND1 and CDKs are actively researched therapeutic targets, evaluated in many clinical trials.[151] Ample evidence exists on the key oncogenic role of CCND1 in lung cancers.[152,153] For example, CCND1 overexpression enhanced lung cancer cell proliferation, invasion, and migration, and arrested the cell cycle at the S phase *in vitro*, overexpression of CCND1 promoted lung cancer growth and metastasis *in vivo*.[154]				
Lung	D	CLTC	Clathrin Heavy Chain | CLTCL2	CLTC is annotated as TSG and fusion cancer driver gene in CGC.[2] Fusions of CLTC and ALK are occasionally detected also in lung cancer: in tissue biopsies[155], as well as circulating tumour DNA from liquid biopsies[156]. Apart from that, we have not found any literature on the oncogenic/TSG role of CLTC in lung cancer.			T F	
Lung	U	CPOX	Coproporphyrinogen Oxidase | CPX | HCP | CPO | COX	CPOX is the sixth enzyme of the haem biosynthetic pathway.[28] We have not found any literature on the role of CPOX in lung cancer.				
Lung	U	HES7	Hes Family BHLH Transcription Factor | Class B Basic Helix-Loop-Helix Protein 37 | Hairy And Enhancer Of Split 7 | BHLHb37 | BHLH Factor Hes7	HES7 is a target of Notch signalling[157], an important pathway in lung cancer.[158] HES7 is an early-stage marker on lung adenocarcinoma[159] and is weakly predictive of poor prognosis of lung adenocarcinoma (but not lung squamous cell carcinoma) in Protein Atlas ($p = 0.0058$)[17].	2			
Lung	U	LINC01023	Long Intergenic Non-Protein Coding RNA 1023	We have not found any literature on the role of LINC01023 in lung cancer. Interestingly, oncogenic role of LINC01023 has been described in liver and brain cancers. In hepatoblastoma cells, LINC01023 silencing attenuated cell proliferation, colony formation and increased cell apoptosis, whereas LINC01023 upregulation results in significant increase in cell proliferation, and colony formation, and xenograft animal tumorgenicity test confirmed the *in vivo* tumorigenesis potential of LINC01203 (preprint).[160] In gliomas, LINC01203 depletion inhibited glioma cell proliferation, migration, and invasion by regulating IGF1R/AKT pathway *in vitro* and *in vivo*.[161]				
Lung	U	PLAU	Plasminogen Activator, Urokinase | UPA | URK	*PLAU*, which is a key component of the long-known oncogenic plasminogen activator system that plays an important role in tumour progression, proliferation, and metastasis via degradation of the extracellular matrix[162,163], is tested as a drug target[162,164], and is a strong predictor of poor survival[165] ($p = 0.0004$ in Protein Atlas), all in/including lung cancer.	4			U***
Lung	U	WWTR1	WW Domain Containing Transcription Regulator 1 | TAZ	A Hippo pathway gene WWTR1 (TAZ) is a CGC oncogene and its oncogenic role in lung cancer has been widely demonstrated, as reviewed by Sardo et al.[166] WWTR1 is overexpressed in lung and other cancers, it promotes proliferation and inhibits apoptosis in lung cancer cells[167], shRNA knockdown of WWTR1 suppresses	4		O	

				metastases *in vivo*[168], constitutively active WWTR1 is a driver for lung tumorigenesis *in vivo*[169], WWTR1 induced PD-L1 upregulation in lung cancer cells is sufficient to inhibit T-cell function[170], WWTR1 promotes aerobic glycolysis and immune escape in lung cancer[171], WWTR1 was suggested to play a role in resistance to therapy[166,172], and play other roles in lung tumorigenesis[166]. Interestingly, WWTR1 is rarely mutated in tumours[172], and its dysregulation in cancer is likely to be driven by other mechanisms, with upregulation of WWTR1 regulated by non-coding mutations being an interesting novel potential mechanism.				
Ovary	U	BCAR1	BCAR1 Scaffold Protein, Cas Family Member | CASS1 | CAS | Cas Scaffolding Protein Family Member | p130Cas	BCAR1 (p130Cas) acts as an oncogene in ovarian and other cancers.[173] High p130cas expression is associated with more advanced ovarian cancer stage and poorer prognosis.[174] Liposomes carrying p130 siRNA reduced growth and increased apoptosis in tumour xenografts.[174] Lysophosphatidic acid stimulates the tyrosine phosphorylation of BCAR1 to promote tumour cell migration.[175] BCAR1 also plays a role in p130Cas inducing tumour cell adhesion to the extracellular matrix and invasion in ovarian cells.[176] Finally, BCAR1 confers resistance to anti-angiogenesis therapy in ovarian cancer.[177]	3			U*
Ovary	U	HCG15	HLA Complex Group 15	HCG15 is a hypoxia-responsive lncRNA that has been shown to facilitate hepatocellular carcinoma cell proliferation and invasion[178]. HCG15 was selected as one of 7 lncRNA markers in a study of ovarian cancer cell line treated with anti-parasite drug ivermectine.[179] We have not found any other literature on the role of HCG15 in ovarian cancer.				
Ovary	U	ID3	Inhibitor Of DNA Binding 3, HLH Protein | BHLHb25 | Inhibitor Of Differentiation | HEIR-1	While being annotated as a TSG in CGC, ID proteins are essential components of oncogenic pathways and are activated transcriptionally and post-transcriptionally by oncogenic factors, ID proteins are overexpressed in many human cancers and deregulation of ID proteins has a direct role in cancer initiation, maintenance, progression, and drug resistance.[180] In ovarian cancer, high ID3 expression is predictive of poor prognosis, being the most significant predictor in a model that accounts for other clinicopathological predictors[181], and in Protein Atlas ($p = 0.006$). [17] Both decreased[182] and increased[183] expression of ID3 has been observed in ovarian cancer compared to normal tissue. Targeting ID3 reduced proliferation and induced cell-cycle arrest and apoptosis in ovarian cancer cells and these effects were counteracted by ectopically overexpressed ID3.[180]	3		T	U**
Ovary	U	MCCC2	Methylcrotonyl-CoA Carboxylase Subunit | MCCB	MCCC2 is a small subunit of 3-methylcrotonyl-CoA carboxylase.[28] Oncogenic properties of MCCC2 have been documented in breast[184], liver[185], colorectal[186], and prostate[187] cancer. MCCC2 was observed to be upregulated in ovarian cancer in a proteomics study.[188]	1			

Ovary	U	PROCA1	Protein Interacting With Cyclin A1	We have not found any literature on the role of PROCA1 in ovarian cancer.				
Ovary	U	ZNF37BP	Zinc Finger Protein 37B, Pseudogene | KOX21	We have not found any literature on the role of ZNF37BP in ovarian cancer.				
Pancreas	U	CCNB1IP1	Cyclin B1 Interacting Protein 1 | Human Enhancer Of Invasion 10 | HEI10 | C14orf18	CCNB1IP1 (HEI10) is an E3 ubiquitin ligase family and functions in progression of the cell cycle through G(2)/M. It is annotated as a CGC TSG, based on expression and survival data in lung cancer[189] and its ability to negatively regulate cell invasion and migration[190]. However, the same study showed that CCNB1IP1 is required for cell proliferation in U20S or MCF7 cells[190] and another study showed that elevated CCNB1IP1 expression correlated with aggressive melanomas[191], suggesting that CCNB1IP1 may have both tumour-suppressive, as oncogenic roles in cancer. Interestingly, non-coding mutations in a cis-regulatory element of CCNB1IP1 have been previously identified, associated with decreased expression of CCNB1IP1, and validated in HEK293 cells that deletion of the elements leads to reduced mRNA abundance and decreased proliferation[192], in line with its potential oncogenic properties. We have not found any literature on the role of CCNB1IP1 in pancreatic cancer, only about the oncogenic role of its interacting protein CCNB1[193].			T	F*
Pancreas	U	OXLD1	Oxidoreductase Like Domain Containing | C17orf90	We have not found any literature on the role of OXLD1 in pancreatic cancer.				F*
Pancreas	U	PARP2	Poly(ADP-Ribose) Polymerase 2 | ARTD2	PARP2 is an important anticancer drug target, with PARP inhibitors being approved and used for patients with BRCA1/2 mutation in a wide range of cancer types, including pancreatic cancer [194–196].	3			F*

References

1. Peter, C. J. *et al.* In vivo epigenetic editing of Sema6a promoter reverses transcallosal dysconnectivity caused by C11orf46/Arl14ep risk gene. *Nature Communications 2019 10:1* **10**, 1–14 (2019).

2. Sondka, Z. *et al.* The COSMIC Cancer Gene Census: describing genetic dysfunction across all human cancers. *Nature Reviews Cancer* vol. 18 696–705 (2018).

3. Strepkos, D., Markouli, M., Klonou, A., Papavassiliou, A. G. & Piperi, C. Histone Methyltransferase SETDB1: A Common Denominator of Tumorigenesis with Therapeutic Potential. *Cancer Research* **81**, 525–534 (2021).

4. Hayashi, T. *et al.* Identification of Transmembrane Protein in Prostate Cancer by the Escherichia coli Ampicillin Secretion Trap: Expression of CDON Is Involved in Tumor Cell Growth and Invasion. *Pathobiology* **78**, 277–284 (2011).

5. Leem, Y. E., Ha, H. L., Bae, J. H., Baek, K. H. & Kang, J. S. CDO, an Hh-Coreceptor, Mediates Lung Cancer Cell Proliferation and Tumorigenicity through Hedgehog Signaling. *PLOS ONE* **9**, e111701 (2014).

6. Mathew, E. *et al.* Dosage-dependent regulation of pancreatic cancer growth and angiogenesis by Hedgehog signaling. *Cell Reports* **9**, 484–494 (2014).

7. Gibert, B. *et al.* Regulation by miR181 Family of the Dependence Receptor CDON Tumor Suppressive Activity in Neuroblastoma. *JNCI: Journal of the National Cancer Institute* **106**, (2014).

8. Uluca, B., Lektemur Esen, C., Saritas Erdogan, S. & Kumbasar, A. NFI transcriptionally represses CDON and is required for SH-SY5Y cell survival. *Biochimica et Biophysica Acta (BBA) - Gene Regulatory Mechanisms* **1865**, 194798 (2022).

9. Rush, S. Z., Abel, T. W., Valadez, J. G., Pearson, M. & Cooper, M. K. Activation of the Hedgehog pathway in pilocytic astrocytomas. *Neuro-Oncology* **12**, 790 (2010).

10. Raleigh, D. R. *et al.* Hedgehog signaling drives medulloblastoma growth via CDK6. *J Clin Invest* **128**, 120–124 (2018).

11. Kim, N. H., Livi, C. B., Yew, P. R. & Boyer, T. G. Mediator subunit Med12 contributes to the maintenance of neural stem cell identity. *BMC Developmental Biology* **16**, 1–15 (2016).

12. Wong, G. C. H. *et al.* Clinical and mutational profiles of adult medulloblastoma groups. *Acta Neuropathologica Communications* **8**, 1–14 (2020).

13. Clark, A. D., Oldenbroek, M. & Boyer, T. G. Mediator kinase module and human tumorigenesis. *Critical Reviews in Biochemistry and Molecular Biology* **50**, 393–426 (2015).

14. Jackson, L. M. *et al.* Loss of MED12 activates the TGFβ pathway to promote chemoresistance and replication fork stability in BRCA-deficient cells. *Nucleic Acids Research* **49**, 12855–12869 (2021).

15. Huang, S. *et al.* MED12 controls the response to multiple cancer drugs through regulation of TGF-β receptor signaling. *Cell* **151**, 937–950 (2012).

16. Caja, L., Bellomo, C. & Moustakas, A. Transforming growth factor β and bone morphogenetic protein actions in brain tumors. *FEBS Letters* **589**, 1588–1597 (2015).

17. Uhlén, M. *et al.* Tissue-based map of the human proteome. *Science (1979)* **347**, (2015).

18. Pollutri, D. & Penzo, M. Ribosomal Protein L10: From Function to Dysfunction. *Cells 2020, Vol. 9, Page 2503* **9**, 2503 (2020).

19. Yu, X. *et al.* Quantitative proteomics reveals the novel co-expression signatures in early brain development for prognosis of glioblastoma multiforme. *Oncotarget* **7**, 14161–14171 (2016).

20. Wang, X. C. *et al.* Role of Cks1 amplification and overexpression in breast cancer. *Biochem Biophys Res Commun* **379**, 1107–1113 (2009).

21. Shi, W. *et al.* CKS1B as Drug Resistance-Inducing Gene—A Potential Target to Improve Cancer Therapy. *Frontiers in Oncology* **10**, 1978 (2020).

22. Ju, G. *et al.* DUSP12 regulates the tumorigenesis and prognosis of hepatocellular carcinoma. *PeerJ* **9**, (2021).

23. Cain, E. L., Braun, S. E. & Beeser, A. Characterization of a Human Cell Line Stably Over-Expressing the Candidate Oncogene, Dual Specificity Phosphatase 12. *PLoS ONE* **6**, (2011).

24. Zandi, Z. *et al.* Dual-specificity phosphatases: therapeutic targets in cancer therapy resistance. *J Cancer Res Clin Oncol* **148**, 57–70 (2022).

25. Boulding, T. *et al.* Differential Roles for DUSP Family Members in Epithelial-to-Mesenchymal Transition and Cancer Stem Cell Regulation in Breast Cancer. *PLoS One* **11**, (2016).

26. Buiga, P., Elson, A., Tabernero, L. & Schwartz, J. M. Kinetic Modeling of DUSP Regulation in Herceptin-Resistant HER2-Positive Breast Cancer. *Genes (Basel)* **10**, (2019).

27. Chang, P.-H. *et al.* Interplay between desmoglein2 and hypoxia controls metastasis in breast cancer. **118**, (2021).

28. Safran, M. *et al.* The GeneCards Suite. *Practical Guide to Life Science Databases* 27–56 (2021) doi:10.1007/978-981-16-5812-9_2.

29. Zhang, M. *et al.* Identification of Tumor-initiating Cells in a p53 Null Mouse Model of Breast Cancer. *Cancer Res* **68**, 4674 (2008).

30. Yazinski, S. A. *et al.* Dual inactivation of Hus1 and p53 in the mouse mammary gland results in accumulation of damaged cells and impaired tissue regeneration. *Proc Natl Acad Sci U S A* **106**, 21282 (2009).

31. Cheng, C. K., Chow, L. W. C., Loo, W. T. Y., Chan, T. K. & Chan, V. The Cell Cycle Checkpoint Gene Rad9 Is a Novel Oncogene Activated by 11q13 Amplification and DNA Methylation in Breast Cancer. *Cancer Research* **65**, 8646–8654 (2005).

32. Balmus, G. *et al.* HUS1 regulates in vivo responses to genotoxic chemotherapies. *Oncogene 2016 35:5* **35**, 662–669 (2015).

33. Deng, W. *et al.* The Role of PPM1D in Cancer and Advances in Studies of Its Inhibitors. *Biomedicine & pharmacotherapy = Biomedecine & pharmacotherapie* **125**, 109956 (2020).

34. Pärssinen, J., Alarmo, E. L., Karhu, R. & Kallioniemi, A. PPM1D silencing by RNA interference inhibits proliferation and induces apoptosis in breast cancer cell lines with wild-type p53. *Cancer Genet Cytogenet* **182**, 33–39 (2008).

35. Yu, E. *et al.* Overexpression of the wip1 gene abrogates the p38 MAPK/p53/Wip1 pathway and silences p16 expression in human breast cancers. *Breast Cancer Res Treat* **101**, 269–278 (2007).

36. Ruark, E. *et al.* Mosaic PPM1D mutations are associated with predisposition to breast and ovarian cancer. *Nature* **493**, 406–410 (2013).

37. Belova, G. I., Demidov, O. N., Fornace, A. J. & Bulavin, D. v. Chemical inhibition of Wip1 phosphatase contributes to suppression of tumorigenesis. *Cancer Biol Ther* **4**, 1154–1158 (2005).

38. Liu, Y. *et al.* Targeting 17q23 amplicon to overcome the resistance to anti-HER2 therapy in HER2+ breast cancer. *Nat Commun* **9**, (2018).

39. Moody, S. E. *et al.* PRKACA mediates resistance to HER2-targeted therapy in breast cancer cells and restores anti-apoptotic signaling. *Oncogene* **34**, 2061–2071 (2015).

40. Yang, F. *et al.* Stabilization of MORC2 by estrogen and antiestrogens through GPER1- PRKACA-CMA pathway contributes to estrogen-induced proliferation and endocrine resistance of breast cancer cells. *Autophagy* **16**, 1061–1076 (2020).

41. Denu, R. A. & Burkard, M. E. Synchronous Bilateral Breast Cancer in a Patient With Nager Syndrome. *Clin Breast Cancer* **17**, e151–e153 (2017).

42. Xiong, F. & Li, S. SF3b4: A Versatile Player in Eukaryotic Cells. *Front Cell Dev Biol* **8**, (2020).

43. Zhou, W. *et al.* SF3B4 is decreased in pancreatic cancer and inhibits the growth and migration of cancer cells. *Tumour Biol* **39**, (2017).

44. Sato, K. & Akimoto, K. Expression Levels of KMT2C and SLC20A1 Identified by Information-theoretical Analysis Are Powerful Prognostic Biomarkers in Estrogen Receptor-positive Breast Cancer. *Clin Breast Cancer* **17**, e135–e142 (2017).

45. ONAGA, C. *et al.* High SLC20A1 Expression Is Associated With Poor Prognoses in Claudin-low and Basal-like Breast Cancers. *Anticancer Res* **41**, 43–54 (2021).

46. Beck, L. *et al.* Identification of a novel function of PiT1 critical for cell proliferation and independent of its phosphate transport activity. *J Biol Chem* **284**, 31363–31374 (2009).

47. Byskov, K. *et al.* Regulation of cell proliferation and cell density by the inorganic phosphate transporter PiT1. *Cell Div* **7**, (2012).

48. Li, J. *et al.* Impact of SLC20A1 on the Wnt/β-catenin signaling pathway in somatotroph adenomas. *Mol Med Rep* **20**, 3276–3284 (2019).

49. O'Malley, S. *et al.* TOB suppresses breast cancer tumorigenesis. *Int J Cancer* **125**, 1805–1813 (2009).

50. Jiao, Y. *et al.* Adenovirus-mediated expression of Tob1 sensitizes breast cancer cells to ionizing radiation. *Acta Pharmacol Sin* **28**, 1628–1636 (2007).

51. Wu, D. *et al.* Tob1 enhances radiosensitivity of breast cancer cells involving the JNK and p38 pathways. *Cell Biol Int* **39**, 1425–1430 (2015).

52. Helms, M. W. *et al.* TOB1 is regulated by EGF-dependent HER2 and EGFR signaling, is highly phosphorylated, and indicates poor prognosis in node-negative breast cancer. *Cancer Res* **69**, 5049–5056 (2009).

53. Zhang, Y. W. *et al.* Acquisition of estrogen independence induces TOB1-related mechanisms supporting breast cancer cell proliferation. *Oncogene* **35**, 1643–1656 (2016).

54. Suzuki, T. *et al.* Phosphorylation of three regulatory serines of Tob by Erk1 and Erk2 is required for Ras-mediated cell proliferation and transformation. *Genes & Development* **16**, 1356 (2002).

55. Phosphorylation of TOB1 at T172 and S320 is critical for gastric cancer proliferation and progression - PubMed. https://pubmed.ncbi.nlm.nih.gov/31497236/.

56. Goswami, M. T. *et al.* Identification of TP53RK-Binding Protein (TPRKB) Dependency in TP53-Deficient Cancers. *Mol Cancer Res* **17**, 1652–1664 (2019).

57. Dempster, J. M. *et al.* Extracting Biological Insights from the Project Achilles Genome-Scale CRISPR Screens in Cancer Cell Lines. *bioRxiv* 720243 (2019) doi:10.1101/720243.

58. Peterson, D. *et al.* A chemosensitization screen identifies TP53RK, a kinase that restrains apoptosis after mitotic stress. *Cancer Res* **70**, 6325–6335 (2010).

59. Hideshima, T. *et al.* p53-related protein kinase confers poor prognosis and represents a novel therapeutic target in multiple myeloma. *Blood* **129**, 1308–1319 (2017).

60. Roh, E. *et al.* Targeting PRPK and TOPK for skin cancer prevention and therapy. *Oncogene* **37**, 5633–5647 (2018).

61. Zykova, T. *et al.* Targeting PRPK Function Blocks Colon Cancer Metastasis. *Mol Cancer Ther* **17**, 1101–1113 (2018).

62. Chen, D. *et al.* Amplitude control of protein kinase C by RINCK, a novel E3 ubiquitin ligase. *J Biol Chem* **282**, 33776–33787 (2007).

63. Lee, S. L. O. *et al.* p34SEI-1 inhibits doxorubicin-induced senescence through a pathway mediated by protein kinase C-delta and c-Jun-NH2-kinase 1 activation in human breast cancer MCF7 cells. *Mol Cancer Res* **7**, 1845–1853 (2009).

64. Shi, W. *et al.* Deciphering the Oncogenic Role of VPS28 Modulated by miR-491-5p in Breast Cancer Cells Using In Silico and Functional Analysis. *Front Mol Biosci* **8**, (2021).

65. Shi, W., Hu, D., Lin, S. & Zhuo, R. Five-mRNA Signature for the Prognosis of Breast Cancer Based on the ceRNA Network. *Biomed Res Int* **2020**, (2020).

66. Oyama, Y. *et al.* 220 Functional analysis of ZFHX4 as a novel therapeutic target in ovarian cancer. *International Journal of Gynecologic Cancer* **30**, A91.2-A91 (2020).

67. Qing, T. *et al.* Somatic mutations in ZFHX4 gene are associated with poor overall survival of Chinese esophageal squamous cell carcinoma patients. *Sci Rep* **7**, (2017).

68. Chudnovsky, Y. *et al.* ZFHX4 interacts with the NuRD core member CHD4 and regulates the glioblastoma tumor-initiating cell state. *Cell Rep* **6**, 313–324 (2014).

69. Millstein, J. *et al.* Prognostic gene expression signature for high-grade serous ovarian cancer. *Ann Oncol* **31**, 1240–1250 (2020).

70. al Amri, W. S. *et al.* Identification of candidate mediators of chemoresponse in breast cancer through therapy-driven selection of somatic variants. *Breast Cancer Res Treat* **183**, 607–616 (2020).

71. Pineda, B. *et al.* A two-gene epigenetic signature for the prediction of response to neoadjuvant chemotherapy in triple-negative breast cancer patients. *Clin Epigenetics* **11**, (2019).

72. Ettenberg, S. A. *et al.* cbl-b inhibits epidermal growth factor receptor signaling. *Oncogene* **18**, 1855–1866 (1999).

73. Pennock, S. & Wang, Z. A Tale of Two Cbls: Interplay of c-Cbl and Cbl-b in Epidermal Growth Factor Receptor Downregulation. *Molecular and Cellular Biology* **28**, 3020 (2008).

74. Wang, S. *et al.* E3 ubiquitin ligases Cbl-b and c-Cbl downregulate PD-L1 in EGFR wild-type non-small cell lung cancer. *FEBS Lett* **592**, 621–630 (2018).

75. Lyle, C. *et al.* c-Cbl targets PD-1 in immune cells for proteasomal degradation and modulates colorectal tumor growth. *Sci Rep* **9**, (2019).

76. Vennin, C. *et al.* H19 non coding RNA-derived miR-675 enhances tumorigenesis and metastasis of breast cancer cells by downregulating c-Cbl and Cbl-b. *Oncotarget* **6**, 29209–29223 (2015).

77. Che, X. *et al.* The E3 ubiquitin ligase Cbl-b inhibits tumor growth in multidrug-resistant gastric and breast cancer cells. *Neoplasma* **64**, 887–892 (2017).

78. Zhang, L. *et al.* The E3 ubiquitin ligase Cbl-b improves the prognosis of RANK positive breast cancer patients by inhibiting RANKL-induced cell migration and metastasis. *Oncotarget* **6**, 22918–22933 (2015).

79. Xu, L. *et al.* E3 Ubiquitin Ligase Cbl-b Prevents Tumor Metastasis by Maintaining the Epithelial Phenotype in Multiple Drug-Resistant Gastric and Breast Cancer Cells. *Neoplasia* **19**, 374 (2017).

80. Zhang, T. *et al.* Suppressed expression of Cbl-b by NF-κB mediates icotinib resistance in EGFR-mutant non-small-cell lung cancer. *Cell Biol Int* **43**, 98–107 (2019).

81. Zhang, G. *et al.* Cbl-b-dependent degradation of FLIPL is involved in ATO-induced autophagy in leukemic K562 and gastric cancer cells. *FEBS Letters* **586**, 3104–3110 (2012).

82. Thell, K. *et al.* Master checkpoint Cbl-b inhibition: Anti-tumour efficacy in a murine colorectal cancer model following siRNA-based cell therapy. *Annals of Oncology* **30**, v503–v504 (2019).

83. Kumar, J. *et al.* Deletion of Cbl-b inhibits CD8 + T-cell exhaustion and promotes CAR T-cell function. *J Immunother Cancer* **9**, (2021).

84. Kumaradevan, S. *et al.* c-Cbl Expression Correlates with Human Colorectal Cancer Survival and Its Wnt/β-Catenin Suppressor Function Is Regulated by Tyr371 Phosphorylation. *Am J Pathol* **188**, 1921–1933 (2018).

85. Shashar, M. *et al.* c-Cbl mediates the degradation of tumorigenic nuclear β-catenin contributing to the heterogeneity in Wnt activity in colorectal tumors. *Oncotarget* **7**, 71136–71150 (2016).

86. Ustyugova, I. v., Zhi, L., Abramowitz, J., Birnbaumer, L. & Wu, M. X. IEX-1 deficiency protects against colonic cancer. *Mol Cancer Res* **10**, 760–767 (2012).

87. Oh, B. Y. *et al.* Exome and transcriptome sequencing identifies loss of PDLIM2 in metastatic colorectal cancers. *Cancer Manag Res* **9**, 581–589 (2017).

88. Segditsas, S. *et al.* Putative direct and indirect Wnt targets identified through consistent gene expression changes in APC-mutant intestinal adenomas from humans and mice. *Hum Mol Genet* **17**, 3864–3875 (2008).

89. Differential expression in normal-adenoma-carcinoma sequence suggests complex molecular carcinogenesis in colon - PubMed. https://pubmed.ncbi.nlm.nih.gov/16969489/.

90. Nambiar, P. R. *et al.* Genetic signatures of high- and low-risk aberrant crypt foci in a mouse model of sporadic colon cancer. *Cancer Res* **64**, 6394–6401 (2004).

91. Yin, W. *et al.* Clinical significance of perioperative EMT-CTC in rectal cancer patients receiving open/laparoscopic surgery. *Neoplasma* **67**, 1131–1138 (2020).

92. Page, A., Navarro, M., Suárez-Cabrera, C., Bravo, A. & Ramirez, A. Context-Dependent Role of IKKβ in Cancer. *Genes (Basel)* **8**, 376 (2017).

93. Pallangyo, C. K., Ziegler, P. K. & Greten, F. R. IKKβ acts as a tumor suppressor in cancer-associated fibroblasts during intestinal tumorigenesis. *J Exp Med* **212**, 2253–2266 (2015).

94. Koliaraki, V., Pasparakis, M. & Kollias, G. IKKβ in intestinal mesenchymal cells promotes initiation of colitis-associated cancer. *J Exp Med* **212**, 2235–2251 (2015).

95. Greten, F. R. *et al.* IKKbeta links inflammation and tumorigenesis in a mouse model of colitis-associated cancer. *Cell* **118**, 285–296 (2004).

96. Vlantis, K. *et al.* Constitutive IKK2 activation in intestinal epithelial cells induces intestinal tumors in mice. *J Clin Invest* **121**, 2781–2793 (2011).

97. Shaked, H. *et al.* Chronic epithelial NF-κB activation accelerates APC loss and intestinal tumor initiation through iNOS up-regulation. *Proc Natl Acad Sci U S A* **109**, 14007–14012 (2012).

98. Konina, D., Sparber, P., Viakhireva, I., Filatova, A. & Skoblov, M. Investigation of LINC00493/SMIM26 Gene Suggests Its Dual Functioning at mRNA and Protein Level. *Int J Mol Sci* **22**, (2021).

99. Man, S. M. *et al.* Critical Role for the DNA Sensor AIM2 in Stem Cell Proliferation and Cancer. *Cell* **162**, 45–58 (2015).

100. Wang, F. *et al.* Akkermansia muciniphila administration exacerbated the development of colitis-associated colorectal cancer in mice. *J Cancer* **13**, 124–133 (2022).

101. Dai, X., Yu, L., Chen, X. & Zhang, J. SNRPD1 confers diagnostic and therapeutic values on breast cancers through cell cycle regulation. *Cancer Cell Int* **21**, (2021).

102. Quidville, V. *et al.* Targeting the deregulated spliceosome core machinery in cancer cells triggers mTOR blockade and autophagy. *Cancer Res* **73**, 2247–2258 (2013).

103. Wang, H. *et al.* The diagnostic and prognostic significance of small nuclear ribonucleoprotein Sm D1 aberrantly high expression in hepatocellular carcinoma. *J Cancer* **13**, 184–201 (2022).

104. Hiratsuka, T. *et al.* ZFP521 contributes to pre-B-cell lymphomagenesis through modulation of the pre-B-cell receptor signaling pathway. *Oncogene* **35**, 3227–3238 (2016).

105. Huan, C., Xiaoxu, C. & Xifang, R. Zinc Finger Protein 521, Negatively Regulated by MicroRNA-204-5p, Promotes Proliferation, Motility and Invasion of Gastric Cancer Cells. *Technol Cancer Res Treat* **18**, 1–10 (2019).

106. Spina, R. *et al.* Critical role of zinc finger protein 521 in the control of growth, clonogenicity and tumorigenic potential of medulloblastoma cells. *Oncotarget* **4**, 1280–1292 (2013).

107. Du, J., Chem, M., Wang, D. & Chen, Y. ZNF521 promotes the progression of colon cancer and is targetedly regulated by miRNA-211-5p. *Journal of Clinical and Pathological Research* **41**, 1237247–1231247 (2021).

108. Duan, R., Du, W. & Guo, W. EZH2: a novel target for cancer treatment. *J Hematol Oncol* **13**, (2020).

109. Chen, S. *et al.* EZH2 promotes hepatocellular carcinoma progression through modulating miR-22/galectin-9 axis. *Journal of Experimental & Clinical Cancer Research : CR* **37**, (2018).

110. Au, S. L. K. *et al.* Enhancer of zeste homolog 2 epigenetically silences multiple tumor suppressor microRNAs to promote liver cancer metastasis. *Hepatology* **56**, 622–631 (2012).

111. Cheng, A. S. L. *et al.* EZH2-mediated concordant repression of Wnt antagonists promotes β-catenin-dependent hepatocarcinogenesis. *Cancer Res* **71**, 4028–4039 (2011).

112. Guo, B., Tan, X. & Cen, H. EZH2 is a negative prognostic biomarker associated with immunosuppression in hepatocellular carcinoma. *PLoS One* **15**, (2020).

113. Li, C. *et al.* Finding an easy way to harmonize: a review of advances in clinical research and combination strategies of EZH2 inhibitors. *Clin Epigenetics* **13**, (2021).

114. Kusakabe, Y. *et al.* EZH1/2 inhibition augments the anti-tumor effects of sorafenib in hepatocellular carcinoma. *Sci Rep* **11**, (2021).

115. Wang, S. *et al.* Inhibition of EZH2 Attenuates Sorafenib Resistance by Targeting NOTCH1 Activation-Dependent Liver Cancer Stem Cells via NOTCH1-Related MicroRNAs in Hepatocellular Carcinoma. *Translational Oncology* **13**, 100741 (2020).

116. Xiao, G. *et al.* EZH2 negatively regulates PD-L1 expression in hepatocellular carcinoma. *J Immunother Cancer* **7**, (2019).

117. Liu, P. *et al.* Activation of NF-kappaB, AP-1 and STAT transcription factors is a frequent and early event in human hepatocellular carcinomas. *Journal of Hepatology* **37**, 63–71 (2002).

118. Koo, J. H. *et al.* Induction of AP-1 by YAP/TAZ contributes to cell proliferation and organ growth. *Genes Dev* **34**, 72–86 (2020).

119. Gao, S., Gang, J., Yu, M., Xin, G. & Tan, H. Computational analysis for identification of early diagnostic biomarkers and prognostic biomarkers of liver cancer based on GEO and TCGA databases and studies on pathways and biological functions affecting the survival time of liver cancer. *BMC Cancer* **21**, (2021).

120. Yamamura, Y., Hua, X., Bergelson, S. & Lodish, H. F. Critical role of Smads and AP-1 complex in transforming growth factor-β-dependent apoptosis. *Journal of Biological Chemistry* **275**, 36295–36302 (2000).

121. Li, H., Li, L., Zheng, H., Yao, X. & Zang, W. Regulatory effects of ΔFosB on proliferation and apoptosis of MCF-7 breast cancer cells. *Tumor Biology* **37**, 6053–6063 (2016).

122. Mooberry, L. K., Sabnis, N. A., Panchoo, M., Nagarajan, B. & Lacko, A. G. Targeting the SR-B1 receptor as a gateway for cancer therapy and imaging. *Frontiers in Pharmacology* vol. 7 (2016).

123. Liu, C. *et al.* Targeted delivery of garcinia glycosides by reconstituted high-density lipoprotein nano-complexes. *Journal of Microencapsulation* **35**, 115–120 (2018).

124. Li, M. *et al.* A dual-targeting reconstituted high density lipoprotein leveraging the synergy of sorafenib and antimiRNA21 for enhanced hepatocellular carcinoma therapy. *Acta Biomaterialia* **75**, 413–426 (2018).

125. Cruz, W. *et al.* Lipoprotein-Like Nanoparticle Carrying Small Interfering RNA Against Spalt-Like Transcription Factor 4 Effectively Targets Hepatocellular Carcinoma Cells and Decreases Tumor Burden. *Hepatology Communications* **4**, 769–782 (2020).

126. Colpitts, C. C. & Baumert, T. F. SCARB1 variants and HCV infection: Host susceptibility is lost in translation. *Journal of Hepatology* vol. 67 211–213 (2017).

127. Zeisel, M. B. *et al.* Scavenger receptor class B type I is a key host factor for hepatitis C virus infection required for an entry step closely linked to CD81. *Hepatology* **46**, 1722–1731 (2007).

128. Danilo, C. *et al.* Scavenger receptor class B type I regulates cellular cholesterol metabolism and cell signaling associated with breast cancer development. *Breast Cancer Research* **15**, (2013).

129. Gordon, J. A. *et al.* Upregulation of scavenger receptor B1 is required for steroidogenic and nonsteroidogenic cholesterol metabolism in prostate cancer. *Cancer Research* **79**, 3320–3331 (2019).

130. Xu, G. hua *et al.* Up-regulation of SR-BI promotes progression and serves as a prognostic biomarker in clear cell renal cell carcinoma. *BMC Cancer* **18**, (2018).

131. Feng, H. *et al.* High scavenger receptor class B type I expression is related to tumor aggressiveness and poor prognosis in lung adenocarcinoma. *Medicine (United States)* **97**, (2018).

132. Bai, Y. *et al.* Identification of Seven-Gene Hypoxia Signature for Predicting Overall Survival of Hepatocellular Carcinoma. *Frontiers in Genetics* **12**, (2021).

133. S, Z. *et al.* The Maturation of Tumor Suppressor miR-497 in Hepatocellular Carcinoma is Inhibited by Oncogenic circRNA SCARB1. *Cancer Management and Research* vol. 13 5761 (2021).

134. Zheng, Y. *et al.* STOML2 potentiates metastasis of hepatocellular carcinoma by promoting PINK1-mediated mitophagy and regulates sensitivity to lenvatinib. *Journal of Hematology and Oncology* **14**, (2021).

135. Zhu, W. *et al.* Silence of stomatin-like protein 2 represses migration and invasion ability of human liver cancer cells via inhibiting the nuclear factor kappa B (NF-κB) pathway. *Medical Science Monitor* **24**, 7625–7632 (2018).

136. Qu, H., Jiang, W., Wang, Y. & Chen, P. Stoml2 as a novel prognostic biomarker modulates cell proliferation, motility and chemo-sensitivity via il6-stat3 pathway in head and neck squamous cell carcinoma. *American Journal of Translational Research* **11**, 683–695 (2019).

137. Zhou, C. *et al.* Enhanced SLP-2 promotes invasion and metastasis by regulating Wnt/β-catenin signal pathway in colorectal cancer and predicts poor prognosis. *Pathology Research and Practice* **215**, 57–67 (2019).

138. Zhang, H., Wu, G., Feng, J., Lu, X. & Liu, P. Expression of STOML2 promotes proliferation and glycolysis of multiple myeloma cells via upregulating PAI-1. *Journal of Orthopaedic Surgery and Research* **16**, (2021).

139. Ma, W. *et al.* STOML2 interacts with PHB through activating MAPK signaling pathway to promote colorectal Cancer proliferation. *Journal of Experimental and Clinical Cancer Research* **40**, (2021).

140. Chao, D. *et al.* Stomatin-like protein 2 induces metastasis by regulating the expression of a rate-limiting enzyme of the hexosamine biosynthetic pathway in pancreatic cancer. *Oncology Reports* **45**, (2021).

141. Popławski, P., Piekiełko-Witkowska, A. & Nauman, A. The significance of TRIP11 and T3 signalling pathway in renal cancer progression and survival of patients. *Endokrynol Pol* **68**, 631–641 (2017).

142. Lin, Y. H., Lin, K. H. & Yeh, C. T. Thyroid Hormone in Hepatocellular Carcinoma: Cancer Risk, Growth Regulation, and Anticancer Drug Resistance. *Frontiers in Medicine* vol. 7 (2020).

143. Chen, R. N. *et al.* Thyroid hormone promotes cell invasion through activation of furin expression in human hepatoma cell lines. *Endocrinology* **149**, 3817–3831 (2008).

144. Fan, H. C. *et al.* Telomeres and Cancer. *Life* vol. 11 (2021).

145. Zhu, J. *et al.* TPP1 OB-fold domain protein suppresses cell proliferation and induces cell apoptosis by inhibiting telomerase recruitment to telomeres in human lung cancer cells. *Journal of Cancer Research and Clinical Oncology* **145**, 1509–1519 (2019).

146. Wang, L. *et al.* FBW7 Mediates Senescence and Pulmonary Fibrosis through Telomere Uncapping. *Cell Metabolism* **32**, 860-877.e9 (2020).

147. Chan, S. W.-L. & Blackburn, E. H. New ways not to make ends meet: telomerase, DNA damage proteins and heterochromatin. *Oncogene* **21**, 553–563 (2002).

148. Wang, Q., Yang, H., Zhou, F., Wu, Q. & Zhou, Y. TPP1 Regulates hTERT Expression and Predicts Early Malignant Event and Prognosis of Cervical Cancer. (2021) doi:10.21203/rs.3.rs-794147/v1.

149. Yang, L. *et al.* Telomere-binding protein TPP1 modulates telomere homeostasis and confers radioresistance to human colorectal cancer cells. *PLoS ONE* **8**, (2013).

150. Gào, X. *et al.* The associations of DNA methylation alterations in oxidative stress-related genes with cancer incidence and mortality outcomes: a population-based cohort study. *Clin Epigenetics* **11**, (2019).

151. Musgrove, E. A., Caldon, C. E., Barraclough, J., Stone, A. & Sutherland, R. L. Cyclin D as a therapeutic target in cancer. *Nature Reviews Cancer 2011 11:8* **11**, 558–572 (2011).

152. Gautschi, O., Ratschiller, D., Gugger, M., Betticher, D. C. & Heighway, J. Cyclin D1 in non-small cell lung cancer: a key driver of malignant transformation. *Lung Cancer* **55**, 1–14 (2007).

153. Montalto, F. I. & de Amicis, F. Cyclin D1 in Cancer: A Molecular Connection for Cell Cycle Control, Adhesion and Invasion in Tumor and Stroma. *Cells* **9**, (2020).

154. Wang, X., Liu, X., Yang, Y. & Yang, D. Cyclin D1 mediated by the nuclear translocation of nuclear factor kappa B exerts an oncogenic role in lung cancer. *Bioengineered* **13**, 6866–6879 (2022).

155. Wang, B. *et al.* Identification of novel ALK fusions using DNA/RNA sequencing in immunohistochemistry / RT-PCR discordant NSCLC patients. *Hum Pathol* **114**, 90–98 (2021).

156. Mondaca, S. *et al.* Clinical utility of next-generation sequencing-based ctDNA testing for common and novel ALK fusions. *Lung Cancer* **159**, 66–73 (2021).

157. Bessho, Y., Miyoshi, G., Sakata, R. & Kageyama, R. Hes7: a bHLH-type repressor gene regulated by Notch and expressed in the presomitic mesoderm. *Genes Cells* **6**, 175–185 (2001).

158. Yuan, X. *et al.* Notch signaling and EMT in non-small cell lung cancer: biological significance and therapeutic application. *J Hematol Oncol* **7**, (2014).

159. Song, J. *et al.* Epithelial-mesenchymal transition markers screened in a cell-based model and validated in lung adenocarcinoma. *BMC Cancer* **19**, (2019).

160. Bhandari, R., Shaikh, I. I., Bhandari, R. & Chapagain, S. LINC01023 Promotes the Hepatoblastoma Tumorigenesis via miR-378a-5p/WNT3 Axis. (2022) doi:10.21203/rs.3.rs-1283178/v1.

161. Yu, M. *et al.* Knockdown of linc01023 restrains glioma proliferation, migration and invasion by regulating IGF-1R/AKT pathway. *J Cancer* **10**, 2961–2968 (2019).

162. Mahmood, N., Mihalcioiu, C. & Rabbani, S. A. Multifaceted role of the urokinase-type plasminogen activator (uPA) and its receptor (uPAR): Diagnostic, prognostic, and therapeutic applications. *Frontiers in Oncology* vol. 8 24 (2018).

163. Provost, J. J. *et al.* Urokinase plasminogen activator receptor induced non-small cell lung cancer invasion and metastasis requires NHE1 transporter expression and transport activity. *Cell Oncol (Dordr)* **35**, 95–110 (2012).

164. Huang, J. *et al.* Intracerebral infusion of the bispecific targeted toxin DTATEGF in a mouse xenograft model of a human metastatic non-small cell lung cancer. *Journal of Neuro-Oncology* **109**, 229–238 (2012).

165. Maynard, A. *et al.* Therapy-Induced Evolution of Human Lung Cancer Revealed by Single-Cell RNA Sequencing. *Cell* **182**, 1232-1251.e22 (2020).

166. Sardo, F. lo, Strano, S. & Blandino, G. YAP and TAZ in lung cancer: Oncogenic role and clinical targeting. *Cancers* vol. 10 (2018).

167. Wang, L. *et al.* WWTR1 promotes cell proliferation and inhibits apoptosis through cyclin A and CTGF regulation in non-small cell lung cancer. *Tumor Biology* **35**, 463–468 (2014).

168. Lau, A. N. *et al.* Tumor-propagating cells and Yap/Taz activity contribute to lung tumor progression and metastasis. *EMBO J* **33**, 468–481 (2014).

169. Yu, J. *et al.* TAZ induces lung cancer stem cell properties and tumorigenesis by up-regulating ALDH1A1. *Oncotarget* **8**, 38426–38443 (2017).

170. Helena, J. J. van R. *et al.* The Hippo Pathway Component TAZ Promotes Immune Evasion in Human Cancer through PD-L1. *Cancer Res* **78**, 1457–1470 (2018).

171. Xie, M., Fu, X. ge & Jiang, K. Notch1/TAZ axis promotes aerobic glycolysis and immune escape in lung cancer. *Cell Death Dis* **12**, (2021).

172. Shreberk-Shaked, M. *et al.* A Division of Labor between YAP and TAZ in Non-Small Cell Lung Cancer. *Cancer Res* **80**, 4145–4157 (2020).

173. Camacho Leal, M. del P. *et al.* p130Cas/BCAR1 scaffold protein in tissue homeostasis and pathogenesis. *Gene* **562**, 1 (2015).

174. Nick, A. M. *et al.* Silencing of p130cas in ovarian carcinoma: a novel mechanism for tumor cell death. *J Natl Cancer Inst* **103**, 1596–1612 (2011).

175. Ward, J. D. & Dhanasekaran, D. N. LPA Stimulates the Phosphorylation of p130Cas via Gαi2 in Ovarian Cancer Cells. *Genes Cancer* **3**, 578–591 (2012).

176. Rea, K. *et al.* Novel Axl-driven signaling pathway and molecular signature characterize high-grade ovarian cancer patients with poor clinical outcome. *Oncotarget* **6**, 30859–30875 (2015).

177. Wen, Y. *et al.* Endothelial p130cas confers resistance to anti-angiogenesis therapy. *Cell Rep* **38**, (2022).

178. Yan, H., He, N. & He, S. HCG15 is a hypoxia-responsive lncRNA and facilitates hepatocellular carcinoma cell proliferation and invasion by enhancing ZNF641 transcription. *Biochem Biophys Res Commun* **608**, 170–176 (2022).

179. Li, N. & Zhan, X. Anti-parasite drug ivermectin can suppress ovarian cancer by regulating lncRNA-EIF4A3-mRNA axes. *EPMA J* **11**, 289–309 (2020).

180. Lasorella, A., Benezra, R. & Iavarone, A. The ID proteins: master regulators of cancer stem cells and tumour aggressiveness. *Nature Reviews Cancer 2014 14:2* **14**, 77–91 (2014).

181. Svoboda, M. *et al.* AID/APOBEC-network reconstruction identifies pathways associated with survival in ovarian cancer. *BMC Genomics* **17**, (2016).

182. Arnold, J. M., Mok, S. C., Purdie, D. & Chenevix-Trench, G. Decreased expression of the Id3 gene at 1p36.1 in ovarian adenocarcinomas. *Br J Cancer* **84**, 352–359 (2001).

183. Shepherd, T. G. & Nachtigal, M. W. Identification of a Putative Autocrine Bone Morphogenetic Protein-Signaling Pathway in Human Ovarian Surface Epithelium and Ovarian Cancer Cells. *Endocrinology* **144**, 3306–3314 (2003).

184. Liu, Y., Yuan, Z. & Song, C. Methylcrotonoyl-CoA carboxylase 2 overexpression predicts an unfavorable prognosis and promotes cell proliferation in breast cancer. *Biomark Med* **13**, 427–436 (2019).

185. Chen, Y. Y. *et al.* MCCC2 promotes HCC development by supporting leucine oncogenic function. *Cancer Cell Int* **21**, (2021).

186. Dai, W., Feng, H. & Lee, D. MCCC2 overexpression predicts poorer prognosis and promotes cell proliferation in colorectal cancer. *Exp Mol Pathol* **115**, (2020).

187. He, J. *et al.* Methylcrotonoyl-CoA Carboxylase 2 Promotes Proliferation, Migration and Invasion and Inhibits Apoptosis of Prostate Cancer Cells Through Regulating GLUD1-P38 MAPK Signaling Pathway. *Onco Targets Ther* **13**, 7317–7327 (2020).

188. Toyama, A. *et al.* Proteomic characterization of ovarian cancers identifying annexin-A4, phosphoserine aminotransferase, cellular retinoic acid-binding protein 2, and serpin B5 as histology-specific biomarkers. *Cancer Sci* **103**, 747–755 (2012).

189. Confalonieri, S. *et al.* Alterations of ubiquitin ligases in human cancer and their association with the natural history of the tumor. *Oncogene* **28**, 2959–2968 (2009).

190. Singh, M. K. *et al.* HEI10 negatively regulates cell invasion by inhibiting cyclin B/Cdk1 and other promotility proteins. *Oncogene* **26**, 4825–4832 (2007).

191. Smith, A. P., Weeraratna, A. T., Spears, J. R., Meltzer, P. S. & Becker, D. SAGE identification and fluorescence imaging analysis of genes and transcripts in melanomas and precursor lesions. *Cancer Biol Ther* **3**, 104–109 (2004).

192. Zhu, H. *et al.* Candidate Cancer Driver Mutations in Distal Regulatory Elements and Long-Range Chromatin Interaction Networks. *Molecular Cell* **77**, 1307-1321.e10 (2020).

193. Zhang, H. *et al.* Effect of CCNB1 silencing on cell cycle, senescence, and apoptosis through the p53 signaling pathway in pancreatic cancer. *J Cell Physiol* **234**, 619–631 (2018).

194. Zhu, H. *et al.* PARP inhibitors in pancreatic cancer: Molecular mechanisms and clinical applications. *Molecular Cancer* vol. 19 1–15 (2020).

195. Tuli, R. *et al.* A phase 1 study of veliparib, a PARP-1/2 inhibitor, with gemcitabine and radiotherapy in locally advanced pancreatic cancer. *EBioMedicine* **40**, 375–381 (2019).

196. Karnak, D. *et al.* Combined inhibition of Wee1 and PARP1/2 for radiosensitization in pancreatic cancer. *Clin Cancer Res* **20**, 5085–5096 (2014).
